# Efficacy and Safety of Tenofovir Disoproxil Fumarate in Asian-Americans with Chronic Hepatitis B in Community Settings

**DOI:** 10.1371/journal.pone.0089789

**Published:** 2014-03-04

**Authors:** Calvin Q. Pan, Huy Trinh, Alan Yao, Ho Bae, Lillian Lou, Sing Chan, for the Study 123 Group

**Affiliations:** 1 Division of Gastroenterology, Department of Medicine, New York University Langone Medical Center, New York University School of Medicine, New York, United States of America; 2 San Jose Gastroenterology, San Jose, California, United States of America; 3 AE & LY Medical Associates, Flushing, New York, United States of America; 4 Asian Pacific Liver Center, St. Vincent Medical Center, Los Angeles, California, United States of America; 5 Nexus Development, Palo Alto, California, United States of America; 6 Sing Chan Endoscopy, Flushing, New York, United States of America; The Chinese University of Hong Kong, Hong Kong

## Abstract

**Background and aims:**

Chronic hepatitis B (CHB) disproportionately affects the Asian-American population in the USA. Tenofovir disoproxil fumarate (TDF) has demonstrated potent antiviral activity in clinical trials, but data in Asian-Americans from community studies are lacking.

**Methods:**

Adult Asian-American patients with CHB from private medical and community-based practices were prospectively enrolled and treated with open-label TDF 300 mg once daily in a single-arm study for 48 weeks. After Week 48, patients had the option to transition to commercially available CHB therapy. The primary efficacy endpoint was hepatitis B virus (HBV) DNA <400 copies/mL at Week 48. Secondary endpoints were safety and tolerability, serologic and biochemical responses, liver fibrosis by FibroTest, and the development of drug-resistant mutations.

**Results:**

Of the 90 patients enrolled, 53 (58%) were hepatitis B e antigen (HBeAg)-positive at baseline. At Week 48, 74 patients (82% overall; 70% HBeAg-positive and 100% HBeAg-negative) had HBV DNA <400 copies/mL. Six (12%) HBeAg-positive patients achieved HBeAg loss/seroconversion. The percentage of patients with alanine aminotransferase in the normal range increased from 26% at baseline to 66% at Week 48. The percentage of patients with F0 (no or minimal) fibrosis by FibroTest increased from 48% to 51%, and those with F4 (severe) fibrosis decreased from 4% to 1%. No resistance to TDF developed. Treatment was well tolerated. Most adverse events were mild in severity and considered unrelated to study drug.

**Conclusions:**

TDF is effective and well tolerated in Asian-American CHB patients in community clinic-based settings, consistent with larger registration trials. Improvement in liver fibrosis was seen in a proportion of patients. No resistance to TDF developed through 48 weeks of treatment.

**Trial Registration:**

Clinicaltrial.gov identifier NCT00736190

## Introduction

Chronic hepatitis B infection (CHB) is a serious public health challenge among Asians and Pacific Islanders residing in the United States. Although comprising only 4.3% of the overall population, Asian-Americans make up half of the estimated 2 million Americans living with CHB, [1] with a reported prevalence of up to 15% depending on the sample population [2]–[4]. CHB-related hepatocellular carcinoma (HCC) has become the most important cancer health disparity affecting Asian-Americans and similar statistics are increasingly being reported in other Western countries [5]. The incidence of HCC among Asian-Americans is more than four times higher than that of Caucasians (11.0 vs. 2.6 per 100,000/year) [6], and HCC is the second highest cause of cancer mortality in Asian-American men, with a 5-year survival rate below 10% [4].

The goal of therapy for CHB is to maintain suppression of viral replication to prevent the emergence of complications, such as cirrhosis, decompensated liver disease, and HCC [7], [8]. Effective suppression of viral replication alters the course of CHB, reducing the histological activity, lessening the risk of progressive liver disease and the HCC incidence [9]–[11]. However, suppression of hepatitis B virus (HBV) replication must be sustained, and clinical benefit is lost if viral replication resumes as a result of insufficient efficacy or the emergence of resistant mutations.

Tenofovir disoproxil fumarate (TDF) is a potent, once-daily nucleotide analog anti-HBV medication recommended as a first-line therapy in CHB [8], [12], [13]. It is also recommended for patients who have developed resistance to lamivudine, entecavir or telbivudine [8]. Good efficacy and safety have been demonstrated in treatment-naïve and -experienced patients in randomised controlled trials of TDF for the treatment of CHB [14]–[17]. Data out to 5 years have shown no development of resistance to TDF, and sustained viral suppression was associated with regression of fibrosis and cirrhosis [15]. TDF has also shown good antiviral potency and tolerability in open-label field studies in Europe, [18]–[21] which demonstrated consistency of results between randomised clinical trials and the more diverse, and potentially more complex, patient population commonly encountered in routine clinical practice. However, the predominant patient populations in the randomised controlled clinical trials and the majority of current field studies for TDF are Caucasians. Although some data have been published for Asian patients from Asia-based cohorts, including those treated in routine clinical practice [reviewed in 22], TDF has not previously been studied in Asian-Americans. . CHB infection is diverse among different racial and ethnic groups in terms of mode of transmission and HBV genotype, which may result in differences in natural history and disease progression [22], [23]. In addition, differences in the social and healthcare setting between Asia and the US may also impact on outcome in routine clinical practice. Given the high prevalence of CHB in Asia and among Asian immigrants to the USA, it is important to understand the clinical profile of TDF in Asian-American CHB patients, particularly in routine clinical practice where most of these patients are cared for. The current study was therefore designed to evaluate the safety, efficacy, and tolerability of TDF in Asian-American adults treated mostly in community clinics.

## Methods

The protocol for this trial and supporting TREND checklist are available as supporting information; see Protocol S1 and Checklist S1.

This was a phase IV, open-label, multicentre, single-arm prospective study in patients with self-reported Asian ancestry residing in the USA (Asian-Americans). Patients included were aged 18 to 75 years, with hepatitis B ‘e’ antigen (HBeAg)-positive or HBeAg-negative CHB, HBV DNA≥10^4^ copies/mL at screening, and alanine aminotransaminase (ALT)>upper limit of normal (ULN), a criterion for treatment according to EASL treatment guidelines [8], and ≤10×ULN at screening or within the 12 months prior to screening. ALT ULN was defined as 43 U/L for males and 34 U/L for females. Patients were required to be naïve to TDF, and to not have received any interferon therapy at least 6 months prior to screening; they may have received other oral anti-HBV nucleoside/nucleotide therapy for fewer than 12 weeks, with the last dose taken at least 16 weeks prior to screening. Patients were excluded if there was history of decompensated liver disease, significant bone disease or renal disease, or evidence of HCC (α-fetoprotein >50 ng/mL, or evidence of HCC on hepatic ultrasound or CT scan).

Patients were enrolled from 19 community-based study sites (13 private medical practices and 6 community-based clinics) in the USA and received TDF 300 mg oral tablets once daily for 48 weeks. Clinical, laboratory and adverse event (AE) assessments were carried out at baseline, Weeks 4, 8 and every 8 weeks thereafter until Week 48. Liver fibrosis was assessed by FibroTest [24] at baseline and at Week 48. All patients were followed up to 48 weeks on treatment as per protocol. The study duration was selected as a fixed time interval to assess response and is in line with other clinical trials [14]. After completion of the trial, subjects were returned to the standard of care provided by the site investigator, and could be transitioned to a commercially available antiviral agent (not provided by the study) or enrolled in a patient assistance program Any patient who permanently withdrew from treatment after receiving at least one dose of study drug was followed for 24 weeks off-treatment or up to initiation of active treatment, whichever occurred first.

### Ethics Statement

The study protocol was approved by the relevant Institutional Review Board (IRB) at each study site; St. Vincent Medical Center IRB (Institutional Review Board), Copernicus Group Independent Review Board, Maimonides Medical Center Research Committee, Palo Alto Medical Foundation Research Institute IRB and the IRB, New York. Written informed consent was obtained from all patients prior to screening. The study was conducted in accordance with recognised international scientific and ethical standards, including the International Conference on Harmonisation guideline for Good Clinical Practice (ICH GCP) and the Declaration of Helsinki (ClinicalTrials.gov identifier: NCT00736190).

### Endpoints

The primary efficacy endpoint was HBV DNA <400 copies/mL (<69 IU/mL) at Week 48. HBV DNA was measured using the Roche COBAS TaqMan HBV 48 test (Roche Molecular Diagnostics, Pleasanton, CA, USA).

Secondary efficacy endpoints included HBV DNA <169 copies/mL (assay lower limit of quantitation), normal or normalised serum ALT levels, change from baseline to Week 48 in FibroTest score (BioPredictive, Paris, France), HBeAg and hepatitis B surface antigen (HBsAg) loss and seroconversion, and the development of drug resistance mutations. FibroTest scores were determined based on a predetermined algorithm and a formula that included the subject's age and sex and five laboratory parameters (α2 macroglobulin, haptoglobin, gamma-glutamyl transferase, bilirubin, and apolipoprotein A1) [24], [25].

Safety and tolerability were evaluated by the occurrence of AEs, serious AEs (SAEs), laboratory abnormalities, discontinuation of the study drug due to AEs, or death. Hepatic flares were defined as elevation of ALT >2× baseline and 10× ULN, or ALT elevation of one grade or twice a previous value that was associated with abnormal laboratory parameters suggestive of worsening of hepatic function. Specific markers of renal abnormalities included confirmed (defined as two consecutive visits) increase in serum creatinine of at least 0.5 mg/dL above baseline value, serum phosphorus values <2 mg/dL, and creatinine clearance <50 mL/min.

HBV genotype (A–H) and resistant mutations within the HBV polymerase/reverse transcriptase (pol/rt) domain were assessed for all enrolled patients at baseline by di-deoxy sequencing and direct hybridization (INNO-LiPA HBV DR versions 2 and 3, Innogenetics, Ghent, Belgium). Di-deoxy sequencing of the pol/rt region was performed in patients with either persistent viremia and who were still viremic at Week 48, at premature treatment discontinuation, or at any time point during the study when virologic breakthrough occurred (defined as a confirmed 1 log_10_ copies/mL increase in HBV DNA from nadir or confirmed HBV DNA >400 copies/mL after having been <400 copies/mL). Amino acid substitutions at conversed sites of the pol/rt were further evaluated phenotypically with *in vitro* cell-culture assays to measure susceptibility to TDF. Detailed procedures have been published elsewhere [26].

### Sample size

Based on previous studies [14], a response rate of 84% was assumed for subjects receiving TDF. Based on the 95% confidence interval for this response rate, from a sample size of 90, 68–83 subjects were expected to achieve virologic response at Week 48. For the safety analysis, 90 patients was considered to have a high probability of capturing the most commonly reported AEs in the pivotal Phase 3 TDF trials (nausea (9%); abdominal pain, diarrhoea, headache, dizziness, fatigue, nasopharyngitis, back pain and skin rash (≥5%)).

### Statistical methods

The primary efficacy and safety analysis set included all patients who received at least one dose of study drug. All categorical endpoints, including the primary efficacy endpoint, were summarised by number and percentage of patients meeting the endpoint. All continuous endpoints were summarised using descriptive statistics. For the primary efficacy analysis, missing data were considered failures of virologic response. Subgroup analyses of the primary efficacy endpoint included analyses by baseline HBeAg status.

## Results

### Study population

**Figure 1:**
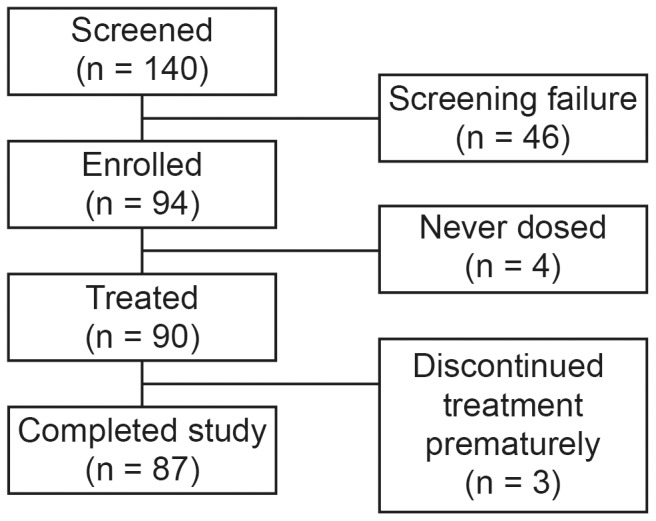
Disposition of study patients.

Patient disposition is shown in Figure 1. Of 140 patients screened, a total of 90 patients received at least one dose of study medication. Three patients discontinued the study early (positive pregnancy test at Week 16, (n = 1) withdrew consent at Week 32, (n = 1) lost to follow-up, unrelated to study drug, (n = 1). Adherence to study medication (based on pill counts) was 98% overall (82–102% in individual subjects).

Baseline characteristics of the patients are shown in Table 1. A majority of patients (64%) were of Chinese origin; 47 (52%) were male and 52 (58%) were HBeAg positive. Only two HBV genotypes were detected, B and C, and all patients had wild-type HBV at baseline. Overall, 26% of patients had ALT≤ULN at baseline, despite having had ALT>ULN at screening, reflecting the natural fluctuation in ALT levels during HBV infection [8].

**Table 1:** Baseline characteristics.

Characteristic	N = 90
Median age, (range)	37 (18–62)
Ethnicity, n (%)	
Chinese	58 (64)
Vietnamese	19 (21)
Korean	12 (13)
Cambodian	1 (1)
Male sex, n (%)	47 (52)
HBV DNA, log_10_ copies/mL, mean (SD)	7.5 (1.8)
HBeAg-positive, n (%)	52 (58)*
ALT, n (%)†	
>ULN	67 (74)
≤ULN	23 (26)
Prior treatment history, n (%)	
Lamivudine	3 (3)
Adefovir	6 (7)
Interferon	5 (6)
Genotype, n (%)	
B	43 (48)
C	47 (52)
FibroTest score, %	
F0	48
F1–F2	46
F3	2
F4	4

*At baseline 52 patients were HBeAg-positive; one patient who was borderline at baseline was HBeAg positive at Week 48.

† Normal range ALT: <34 U/L female; <43 U/L male.

ALT, alanine aminotransferase; HBeAg, hepatitis B ‘e’ antigen; HBV, hepatitis B virus; SD, standard deviation; ULN, upper limit of normal.

### Efficacy

**Figure 2:**
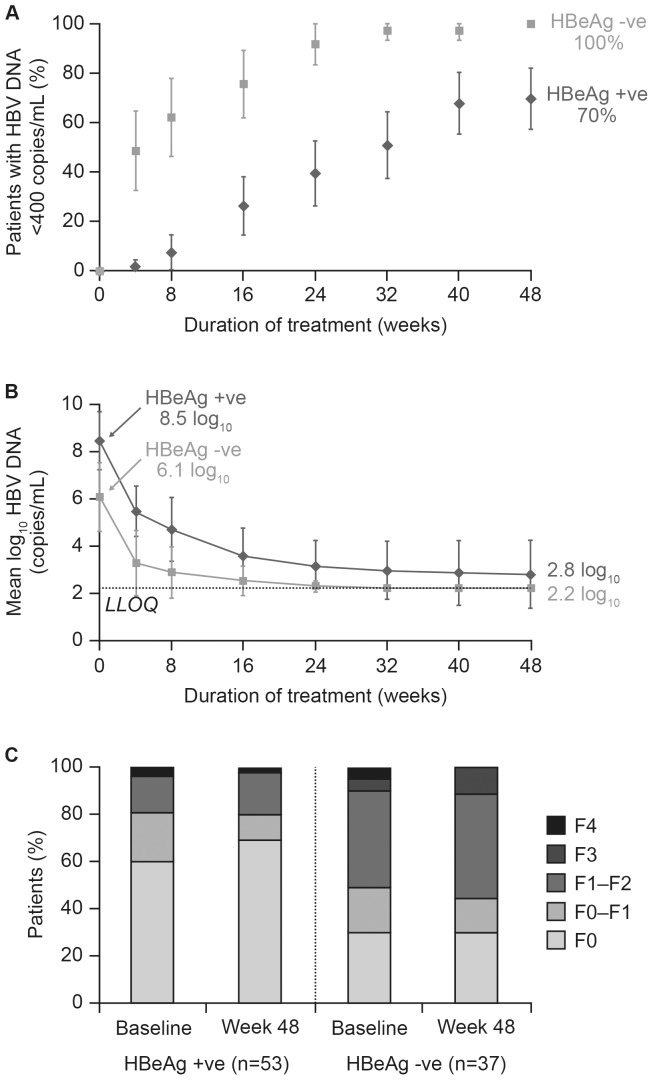
Efficacy of tenofovir in Asian-Americans. (**A**) **Proportion of HBeAg-positive and HBeAg-negative patients with HBV DNA <400 copies/mL by treatment week;** (**B**) **Mean change from baseline in HBV DNA in HBeAg-positive and HBeAg-negative patients by treatment week;** (**C**) **Fibrotest score categories at baseline and Week 48 in HBeAg-positive and HBeAg-negative patients.** HBeAg, hepatitis B ‘e’ antigen; LLOQ, lower limit of quantitation.

The results of the efficacy analyses are shown in Table 2. Overall, 21% of patients achieved HBV DNA <400 copies/mL by Week 4, 61% by Week 24, and 82% at Week 48 following treatment with TDF. Virologic response was achieved by 100% of HBeAg-negative and 70% of HBeAg-positive patients at Week 48 (Figure 2A). The higher percentage of virologic response at Week 48 in HBeAg-negative patients was associated with markedly lower HBV DNA at baseline in these patients (mean ± standard deviation (SD) 6.1±1.4 log_10_ copies/mL) compared with HBeAg-positive patients (8.5±1.2 log_10_ copies/mL). The initial rate of HBV DNA decline was similar between HBeAg-negative and -positive patients, with a decline from baseline to Week 4 of 2.8±0.8 and 3.0±0.7, respectively (Figure 2B). The change in HBV DNA from baseline to Week 48 was 4.9±1.8 log_10_ copies/mL overall and was similar between male and female patients (4.8±1.8 and 5.1±1.8 log_10_ copies/mL, respectively).

**Table 2:** Summary of efficacy results at Week 48.

Endpoint	HBeAg-positive (n = 53)	HBeAg-negative (n = 37)	Overall (n = 90)
HBV DNA <400 copies/mL, n (%), (95% CI)	37 (70)	37 (100)	74 (82)
	(57.5, 82.2)	(100, 100)	(74.3, 90.1)
HBV DNA <169 copies/mL, n (%), (95% CI)	36 (68)	38 (100)	73 (81)
	(55.4, 80.5)	(100, 100)	(73.0, 89.2)
Normal ALT, n (%), (95% CI)	32 (60)	27 (73)	59 (66)
	(47.2, 73.5)	(58.7, 87.3)	(55.7, 75.4)
HBV DNA <400 copies/mL+ALT≤ULN, n (%), (95% CI)	28 (53)	27 (73)	55 (61)
	(39.4, 66.3)	(58.7, 87.3)	(51.0, 71.2)
HBeAg loss and seroconversion, n (%), (95% CI)	6 (12)	-	6 (12)
	(2.8, 19.9)		(1.5, 11.5)

ALT, alanine aminotransferase; CI, confidence interval; HBeAg, hepatitis B ‘e’ antigen; HBV, hepatitis B virus; ULN, upper limit of normal.

The percentage of patients with ALT in the normal range increased from 26% at baseline to 66% at Week 48; 60% (59% male; 61% female) of those patients with elevated ALT at baseline had normalized ALT at Week 48. Overall mean ALT level at baseline was 103.56±149.6 U/L and change from baseline to Week 48 was −68.4±152.8 resulting in a level of 35.7±17.9 U/L at Week 48. In all, 61% of patients met the composite virologic and biochemical endpoint criteria (HBV DNA <400 copies/mL and ALT≤ULN) at Week 48 (Table 2). Of 52 patients who were HBeAg positive at baseline, 6 (12%) lost HBeAg and seroconverted to anti-HBe by Week 48. No patient had HBsAg loss during this 48-week study.

### FibroTest

The mean baseline Fibrotest value was 0.266. The mean value at week 48 was 0.262. The proportion of patients with F0 (no or minimal) fibrosis increased from 48% to 51% during the study, and the percentage of patients with F4 (severe) fibrosis decreased from 4% to 1% (Figure 2C). Among HBeAg-positive patients, the percentage with F0 (no) fibrosis increased from baseline to Week 48 (from 60% to 67%), and the percentage with F4 (severe) fibrosis decreased (from 4% to 2%). Among HBeAg-negative patients, the percentage of patients with F0 fibrosis decreased slightly from baseline to Week 48 (from 30% to 29%; difference of one patient), and the percentage of patients with F4 fibrosis decreased from 5% to 0%. Two patients had F3 fibrosis at baseline and both improved to F2 at Week 48. Four patients had F4 fibrosis at baseline and three had improvement to F3 or F1–F2 at Week 48. Two patients with F1–F2 or F2 at baseline worsened to F3 at Week 48. No patient had worsening to F4.

### Safety

Overall, 50 patients (56%) had at least one AE that occurred during the study. AEs that occurred during the study in ≥5% of patients are shown in Table 3. The majority of AEs were mild (Grade 1) in severity and considered unrelated to study drug. Two (2%) patients had a Grade 3–4 AE (elevations of ALT and aspartate transaminase (AST) in one patient, and cirrhosis, hepatic neoplasm malignant, and esophageal varices in one patient who discontinued from study prematurely and subsequently was lost to follow up); these were considered by the investigator to be unrelated to study drug. A total of nine patients (10%) had at least one AE considered related to study drug by the investigator, of which only nausea and renal creatinine clearance decreased were reported in more than one patient (Table 3).

**Table 3:** Adverse events.

AEs	N = 90
AEs occurring during the study in ≥5% patients, n (%)	
Headache	6 (7)
Nasopharyngitis	6 (7)
Nausea	5 (6)
Weight decrease	5 (6)
Rash	5 (6)
Study drug-related adverse events occurring in >1 patient	
Nausea	3 (3)
Creatinine renal clearance decreased	2 (2)
Serious adverse events	
HCC (not related to study drug)	1 (1)

AE, adverse event; HCC, hepatocellular carcinoma.

**Figure 3:**
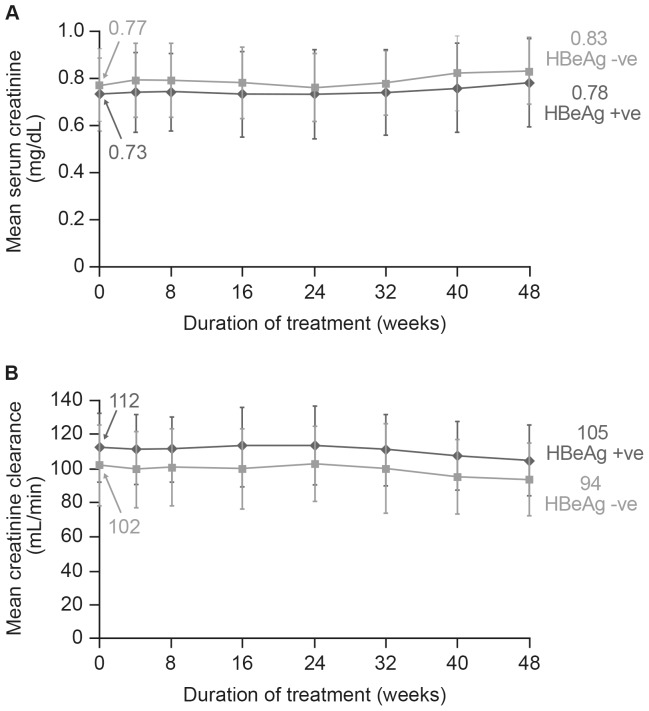
Mean change from baseline in serum creatinine level (A) and clearance rate (B) in HBeAg-positive and HBeAg-negative patients by treatment week. HBeAg, hepatitis B ‘e’ antigen.

The overall mean ± SD serum creatinine value was 0.75±0.16 mg/dL at baseline and 0.80±0.17 mg/dL at Week 48 (Figure 3A). Overall mean ± SD calculated creatinine clearance was 118.2±25.3 mL/min at baseline and 107.1±23.0 mL/min at Week 48 (Figure 3B). No patient had confirmed ≥0.5 mg/dL increase in serum creatinine, or confirmed creatinine clearance <50 mL/min. One patient had a confirmed serum phosphorus <2.0 mg/dL at the final visit (not associated with creatinine increase). No renal function SAEs were reported and there were no renal AEs resulting in dose modification, interruption, or discontinuation of study drug treatment. No hepatic AEs (including hepatic flares) considered to be treatment-related were reported.

### Resistance surveillance

Of the 90 patients included in the study, 14 (16%) had HBV DNA≥400 copies/mL at Week 48 (n = 12) or at premature treatment discontinuation (n = 2). No genotypic substitutions in HBV pol/rt associated with decreased sensitivity to TDF were detected in sera from these patients. None of the patients in this study developed resistance to TDF.

## Discussion

This study is the first to report data from community clinic-based settings for TDF in Asian-Americans with CHB treated in routine clinical practice in a large number of US centers. The efficacy and safety of TDF in this population was comparable to that reported in mostly non-Asians in pivotal trials and field studies in Europe. TDF demonstrated potent viral suppression, was well tolerated and no resistance developed through 48 weeks of treatment.

At Week 48 in the pivotal phase III trials of TDF in CHB, which included predominantly Caucasian populations, 93% of HBeAg-negative and 76% of HBeAg-positive patients had HBV DNA <400 copies/mL, with 68% and 76%, respectively, achieving normalization of ALT levels and 21% achieving HBeAg seroconversion [14]. In the current study, the virological response at Week 48, as measured by the percentage of patients who achieved HBV DNA <400 copies/mL, was lower in HBeAg-positive compared with HBeAg-negative patients. The proportion of patients with undetectable HBV DNA following short-term (one year) therapy with nucleos(t)ide analogues is lower than that seen in HBeAg-negative patients [27]. Among Asian patients, the most common mode of HBV transmission is vertical, from mother to child, and features a long immune tolerance phase with high levels of HBV DNA and persistent HBeAg positivity [22]. Serum HBV DNA levels are significantly higher in HBeAg-positive compared with HBeAg-negative CHB [28]. In keeping with this, HBeAg-positive patients in our study had a mean baseline HBV DNA of 8.5 log copies/mL vs. 6.1 log copies/mL for HBeAg-negative patients. Studies have shown that both high baseline HBV DNA and HBeAg positivity are associated delayed on-treatment reduction in HBV DNA [29] and significantly slower clearance of HBV virions and infected cells [30]. HBeAg-positive patients with higher levels of viraemia may subsequently require a longer duration of antiviral treatment to achieve undetectable HBV DNA levels. The 48 week treatment duration specified in the current study may therefore have contributed to the lower proportion of HBeAg-positive patients with undetectable HBV at the end of the study compared to HBeAg-negative patients. Long term data have shown response rates of 99% in HBeAg-positive patients who remained on treatment with TDF for 5 years [15].

A subgroup analysis of the 189 (94 HBeAg-negative; 95 HBeAg-positive) Asian patients included in these trials demonstrated similar efficacy and safety to the overall population (85% HBV DNA <400 copies/mL; 72% normalised ALT), but also lower rates of HBeAg seroconversion (16%) at Week 48 [31] which was similar to the findings of the current study. Following up to 5 years of long-term TDF treatment in patients included in the pivotal phase III trials, virologic response to TDF was durable, with associated increases in the rate of both HBeAg and HBsAg loss and seroconversion over time, [15] and in the Asian subgroup, the rate of HBeAg seroconversion rose to 40%, similar to 41% of the non-Asians [28]. No patient in the current study cleared HBsAg, consistent with findings in the Asian subgroup of the TDF phase III trials, where no Asian patient showed HBsAg loss during up to 5 years of treatment [32]. Overall, Asian patients appear to be less likely than Caucasians to lose HBsAg following antiviral therapy [23], [33]. The reasons for this are not fully elucidated, but HBV infection in Asians is associated with a number of linked factors, including the predominance of HBV genotype B or C and of vertical or perinatal transmission.

The goal of treatment for CHB is suppression of viral replication to prevent progression to fibrosis and ultimately sequelae such as hepatic decompensation and HCC. An increasing body of evidence shows that fibrosis, previously considered to be irreversible, can regress if the underlying cause of the liver disease is removed [34], [35]. In the phase III trials of TDF, continuous suppression of HBV replication by TDF for 5 years has been shown to be associated with regression of fibrosis and reversal of cirrhosis in a majority of patients [15]. In the subgroup of Asian patients, 90.3% of 72 patients showed no worsening or histological improvement in fibrosis [28]. Of 18 Asian patients with cirrhosis at baseline, 16 (89%) were no longer cirrhotic (Ishak fibrosis score <5) at Year 5 [32]. In the current study, almost half of patients had no/mild fibrosis and 4% had severe fibrosis as assessed by FibroTest, compared with 0% and 20%, respectively in the phase III TDF trials (assessed by liver biopsy) [14]. The fact that most patients were in the earlier stages of liver disease in our cohort likely reflects a tendency to refer patients with more advanced disease to specialist centres such as those who participated in the pivotal TDF study rather than manage them in the community setting. While it may also reflect differences in the method of assessing fibrosis, this is unlikely to be significant. While liver biopsy has generally been considered the ‘gold standard’, non-invasive testing for the serial assessment of fibrosis during therapy is particularly relevant to routine clinical practice. FibroTest is a non-invasive marker of liver fibrosis widely used in Europe, which determines a fibrosis score using the combination of five laboratory tests together with age and sex in a predefined algorithm [36]. This test has been evaluated in a variety of liver diseases, including CHB, [24], [37], [38] and has been validated in an Asian population [34]. The diagnostic value of FibroTest is best for extreme stages (i.e. no fibrosis vs. severe fibrosis), and is lower for discriminating intermediate fibrosis stages [24]. In the current study, there was an increase in the proportion of patients with a FibroTest Stage score of 0 (no fibrosis) and a corresponding decrease in the proportion of patients with a FibroTest Stage score of 4 (severe fibrosis) between baseline and Week 48 in both HBeAg-positive and -negative patients. As regression of fibrosis is likely to be a long-term process, and given the 5-year results of fibrosis regression from the phase III TDF trials, the data at Week 48 from the current study are encouraging.

The safety profile of TDF in this study was similar to that reported in clinical trials and other field studies in the wider patient population. There were no unexpected AEs, no evidence of renal or hepatic toxicity, and treatment was well-tolerated with 97% of patients remaining on-treatment. No genotypic substitutions associated with decreased sensitivity to TDF arose in the current study, and no patient developed resistance to TDF, including those patients with continued viremia or who experienced virologic breakthrough, which, in the opinion of the investigators, was largely contributed to by lack of adherence to medication. These findings are in line with other studies in which no resistance development was detected following up to 5 years of therapy in both treatment-naïve and -experienced patients [15], [39]. Compared with patients with HBV DNA <400 copies/mL at week 48, patients with continued viremia without virologic breakthrough had higher mean baseline HBV DNA levels. All of these patients had decreases in HBV DNA during the study, with a mean decline at Week 48 of 6.2 log_10_ copies/mL. These data suggest that longer treatment with TDF may result in a further reduction in HBV DNA levels in these patients.

This was a prospective study, carried out in Asian-American patients managed in a number of community practices throughout the USA, and therefore has greater validity compared with retrospective or single-center field studies. The enrolment of patients in the community setting means that the study included a range of patients reflecting those encountered in routine clinical practice, including patients who might not be eligible for clinical trials, for example due to comorbidities. The focus on patients who received care in the community setting provides confirmation of the efficacy and safety of TDF treatment in patients receiving less structured care and healthcare professional support than would be seen in clinical trials or in specialist referral centres. In addition, patients managed in referral centres may represent a different population compared with those in the community setting, as they may be specifically selected by referring physicians. The limitations of this study relate to its open-label nature and relatively short (1-year) duration.

In summary, the clinic-based data in Asian-Americans provided by the current study support the efficacy and safety of TDF reported in clinical trials. TDF provides potent suppression of HBV DNA in Asian-Americans with either HBeAg-positive or HBeAg-negative CHB with a tolerability and efficacy profile largely consistent with that in other CHB patient populations. Treatment with TDF for 48 weeks resulted in improvement in liver fibrosis in a proportion of patients as demonstrated by non-invasive assessment. No resistance to TDF developed over the 48-week treatment period. Further studies are required to confirm the longer term efficacy of TDF in Asian-American patients.

## Supporting Information

Checklist S1 (PDF)

Protocol S1 (PDF)
