# Efficacy and Safety of Tenofovir Disoproxil Fumarate in Asian-Americans with Chronic Hepatitis B in Community Setting

**DOI:** 10.1371/journal.pone.0098723

**Published:** 2014-05-23

**Authors:** The PLOS ONE Staff

Dr. Sing Chan is incorrectly listed as corresponding author. The correct corresponding author is Calvin Q. Pan (cpan100@gmail.com). The publisher apologizes for this error.
