# Supplementary material for: Efficacy and Safety of Tenofovir Disoproxil Fumarate in Asian-Americans with Chronic Hepatitis B in Community Settings
Source: PLoS One. 2014 Mar 4;9(3):e89789. doi: 10.1371/journal.pone.0089789 (PMC3942404; doi:10.1371/journal.pone.0089789)
Supplement: Protocol S1 — (PDF) [file pone.0089789.s002.pdf]

CLINICAL STUDY PROTOCOL

**Study Title:**	A Phase IV Study to Evaluate the Efficacy, Safety and Tolerability of Tenofovir DF in Asian-American Adults with Chronic Hepatitis B Infection
**Sponsor:**	Gilead Sciences, Inc.<br>4 University Place<br>4611 University Drive<br>Durham, NC 27707
**IND Number:**<br>**EudraCT Number:**	71,576<br>Not Applicable
**Indication:**	Chronic Hepatitis B
**Protocol ID:**	GS-US-174-0123
**Gilead Sciences Medical Monitor:**	Name: Elizabeth Fagan, MD<br>Telephone: (650) 522-4286<br>Cell Phone: (650) 504-6703<br>Fax: (650) 522-5473
**Protocol Version/Date:**	Original: 14 April 2008<br>Administrative Amendment 1: 26 August 2008

CONFIDENTIALITY STATEMENT

The information contained in this document, particularly unpublished data, is the property or under control of Gilead Sciences, Inc., and is provided to you in confidence as an investigator, potential investigator, or consultant, for review by you, your staff, and an applicable Institutional Review Board or Independent Ethics Committee. The information is only to be used by you in connection with authorized clinical studies of the investigational drug described in the protocol. You will not disclose any of the information to others without written authorization from Gilead Sciences, Inc., except to the extent necessary to obtain informed consent from those persons to whom the drug may be administered.

TABLE OF CONTENTS

1. INTRODUCTION 13
1.1. Background 13
1.2. Tenofovir Disoproxil Fumarate (Tenofovir DF) 14
1.2.1. General Information 14
1.2.2. Pre-Clinical Pharmacology and Toxicology 15
1.2.3. Clinical Trials of Tenofovir DF 17
1.2.4. Summary of Safety of Tenofovir DF in HIV-1 Mono-Infected and HBV and HIV-1 Co-Infected Subjects 23
1.3. Rationale for the Current Study and Dose Selection 25
1.3.1. Rationale for Tenofovir DF Dose Selection 25
1.3.2. Rationale for FibroTest 26
2. OBJECTIVES 27
3. STUDY DESIGN 28
4. SUBJECT POPULATION 30
4.1. Number of Subjects and Subject Selection 30
4.2. Inclusion Criteria 30
4.3. Exclusion Criteria 30
4.4. Study-Specific Tolerance and Inclusion/Exclusion Criteria 32
5. STUDY DRUGS 33
5.1. Treatment Assignment 33
5.2. Description and Handling of Study Drug 33
5.2.1. Formulation 33
5.2.2. Packaging and Labeling 33
5.2.3. Storage and Handling 34
5.3. Dosage and Administration of Study Drugs 34
5.4. Prior and Concomitant Medications 34
5.4.1. Prior to Study Entry 34
5.4.2. During the Study 35
6. STUDY PROCEDURES 36
6.1. Subject Enrollment and Treatment Assignment 36
6.1.1. Screening Visit 36
6.1.2. Baseline Assessments 37
6.2. Treatment Assessments 38
6.2.1. Week 4, 8, 16, 24, 32 and 40 Assessments 38
6.2.2. Week 48 Assessments 39
6.3. Post-Treatment/Treatment-Free Follow-up Assessments 40
6.3.1. Six-Month Post-Treatment Follow-Up Assessments 40
6.3.2. Assessments for Premature Discontinuation from the Study (Early Study Drug Discontinuation Visit) 41
6.4. Tenofovir Plasma Concentration Determination 41
6.5. Serum and Plasma for Storage 41
6.6. Resistance Surveillance 41
6.7. Criteria for Discontinuation of Study Treatment 42
7. ADVERSE EVENTS AND TOXICITY MANAGEMENT 43
7.1. Adverse Events 43

7.2. Assessment of Adverse Events 43
7.3. Serious Adverse Events 44
7.3.1. Overdose 46
7.4. Serious Adverse Event Reporting Requirements 46
7.4.1. All Serious Adverse Events....46
7.4.2. Investigator and Sponsor Reporting Requirements for SAEs 47
7.4.3. Post-Study Reporting Requirements 47
7.5. Clinical Laboratory Abnormalities and Other Abnormal Assessments as Adverse Events or Serious Adverse Events 48
7.6. Toxicity Management (Other Than On-Treatment ALT Flare, Post-Treatment Exacerbation of Hepatitis, or Serum Creatinine Elevation/Decreased Creatinine Clearance) 48
7.6.1. Grades 1 and 2 Laboratory Abnormality or Clinical Event....49
7.6.2. Grade 3 Laboratory Abnormality or Clinical Event....49
7.6.3. Grade 4 Laboratory Abnormality or Clinical Event....49
7.6.4. Management of Elevated Serum Creatinine and Decreased Creatinine Clearance....50
7.6.5. On-Treatment ALT Flare and Post-Treatment Exacerbation of Hepatitis Management 50
7.7. Risks for Women of Childbearing Potential or During Pregnancy 53
8. STATISTICAL CONSIDERATIONS....55
8.1. Analysis Objectives....55
8.2. Primary Endpoint 55
8.3. Secondary Endpoints....55
8.4. Methods of Analysis 56
8.4.1. Analysis Sets 56
8.4.2. Data Handling Conventions 56
8.5. Demographic Data and Baseline Characteristics 57
8.6. Efficacy Analysis 57
8.6.1. Primary Analysis....57
8.6.2. Secondary Analyses 57
8.7. Safety Analysis 58
8.7.1. Extent of Exposure....58
8.7.2. Adverse Events 58
8.7.3. Laboratory Evaluations 59
8.8. Pharmacokinetic Analysis....59
8.9. Sample Size....59
9. RESPONSIBILITIES 60
9.1. Investigator Responsibilities 60
9.1.1. Good Clinical Practice 60
9.1.2. Institutional Review Board/Independent Ethics Committee Approval 60
9.1.3. Informed Consent....61
9.1.4. Confidentiality 61
9.1.5. Study Files and Retention of Records 61
9.1.6. Case Report Forms....62
9.1.7. Drug Accountability....62
9.1.8. Inspections 63
9.1.9. Protocol Compliance 63
9.2. Sponsor Responsibilities....63
9.2.1. Protocol Modifications....63
9.2.2. Study Report and Publications 63

9.3. Joint Investigator/Sponsor Responsibilities 64
9.3.1. Access to Information for Monitoring 64
9.3.2. Access to Information for Auditing or Inspections 64
9.3.3. Study Discontinuation 64

10. REFERENCES 65

11. APPENDICES 74

Appendix 1. Investigator Signature Page 75
Appendix 2. Study Procedures Table 76
Appendix 3. Clinical Laboratory Assessments 78
Appendix 4. Blood Sample Collection and Processing for Determination of Tenofovir Concentrations in Plasma 79
Appendix 5. Management of Clinical and Laboratory Adverse Events 81
Appendix 6. GSI Grading Scale for Severity of Adverse Events and Laboratory Abnormalities 82
Appendix 7. Summary of Anti-Viral Activity of Tenofovir DF in Chronic Hepatitis B Patients* or Chronic Hepatitis B Patients Co-infected with HIV** 93
Appendix 8. Lactic Acidosis Guidelines 113

PROTOCOL SYNOPSIS

**Gilead Sciences, Inc.
4 University Place
4611 University Drive
Durham, NC 27707**

**Study Title:**	A Phase IV Study to Evaluate the Efficacy, Safety and Tolerability of Tenofovir DF in Asian-American Adults with Chronic Hepatitis B Infection
**IND Number:**	71,576
**EudraCT Number:**	Not Applicable
**Study Centers Planned:**	Up to 40 centers in the United States

Objectives:

The primary objective of this study is:

- To evaluate the antiviral efficacy of tenofovir DF 300 mg once daily in Asian-American adults with chronic hepatitis B (CHB) infection

Secondary objectives are:

- To evaluate the safety and tolerability of tenofovir DF in Asian-American adults with CHB infection
- To evaluate the biochemical and serological responses to tenofovir DF in Asian-American adults with CHB infection
- To evaluate the incidence of drug resistance mutations
- To evaluate change from baseline to Week 48 in FibroTest, a non-invasive biomarker of liver fibrosis

**Study Design:**	This is a Phase IV, open-label, single-arm 48-week study evaluating the antiviral efficacy, safety, and tolerability of tenofovir DF in HBV mono-infected Asian-American adults. Ninety adults aged 18–75 with chronic HBV infection (HBV DNA $\geq 10^4$ copies/mL; ALT > ULN and ≤ 10 × ULN at screening or within the past 12 months, as documented in the subject's medical record) will receive treatment with open-label tenofovir DF 300 mg PO once daily.<br><br>Subjects must be naive to tenofovir DF, but may have taken < 12 weeks of oral anti-HBV nucleoside/nucleotide therapy, with the last dose ≥ 16 weeks prior to screening. Subjects must have discontinued interferon ≥ 6 months prior to screening.<br><br>Enrollment will be capped at 45 males to ensure adequate participation by Asian-American females. No more than 10 subjects may be enrolled at a single site without prior written approval from Gilead Sciences.<br><br>The participation of each subject will end after 48 weeks of treatment, at which time subjects may be transitioned to commercially-available HBV drugs (not provided by the study) or enrolled in a patient assistance program. Subjects who discontinue tenofovir DF at the end of 48 weeks and do not immediately initiate commercially-available HBV treatment (via prescription or patient assistance program) must be followed for an additional 6 months or until they initiate commercially-available HBV treatment, whichever occurs first.
Number of Subjects Planned:	90 eligible subjects will receive open-label tenofovir DF treatment
Target Population:	Adult Asian-American subjects (aged 18–75) with chronic HBV infection, who are naïve to tenofovir DF treatment
Duration of Treatment:	Subjects will be treated with open-label tenofovir DF for 48 weeks, at which time they will transition to commercially-available HBV therapy. The study duration for a given subject is at least 54 weeks (including a screening period of up to 6 weeks). Subjects who discontinue tenofovir DF at the end of 48 weeks and do not initiate commercially-available HBV treatment must be followed for an additional 6 months or until they initiate commercially-available HBV treatment, whichever occurs first.

Diagnosis and Main Eligibility Criteria:	At screening, adult Asian-American subjects (18–75 years of age) with chronic HBV infection (HBsAg positive for at least 6 months), with HBV DNA ≥ 10^4 copies/mL, ALT > ULN and ≤ 10 × ULN at screening or within the past 12 months (as documented in the subject's medical record), creatinine clearance ≥ 60 mL/min/1.73 m^2 and normal platelet count will be eligible for the study. Subjects must be naive to tenofovir DF therapy, but could have taken < 12 weeks of oral anti-HBV nucleoside/nucleotide therapy, with the last dose ≥ 16 weeks prior to screening. Subjects must have discontinued interferon ≥ 6 months prior to screening. Subjects must be without serological evidence of co-infection with HIV, HCV or HDV. Subjects with a history of significant bone disease, decompensated liver disease, evidence of hepatocellular carcinoma (i.e., α-fetoprotein > 50 ng/mL), and pregnant or breast-feeding females will not be eligible for the study. Subjects > 40 years of age with α-fetoprotein ≤ 50 ng/mL must undergo a hepatic ultrasound or computed tomography (CT) scan to rule out hepatocellular carcinoma.
Study Procedures/ Frequency:	Plasma HBV DNA levels, laboratory analyses (serum chemistry, liver tests, hematology, and urinalysis), pregnancy test (for females of child bearing potential), vital signs, weight, adverse events and concomitant medications will be measured or assessed at Screening, Baseline, and Weeks 4, 8, 16, 24, 32, 40, and 48. Height will be measured at the Screening and Baseline Visits. HBV serology (HBsAg, HBeAg, and reflex HBeAb and HBsAb if Ag negative) will be conducted at Screening, Baseline and every 16 weeks through Week 48. Complete physical examinations will be performed at Screening, Baseline, and Weeks 24 and 48; symptom-directed physical examinations will be performed at all other visits. Determination of HBV viral genotype (A-H) will be performed at Baseline for all subjects. Genotypic analysis of the HBV polymerase by di-deoxy sequencing will be conducted at Baseline for all subjects, and attempted for all viremic subjects (HBV DNA ≥ 400 copies/mL) at Week 48. In addition, the INNOLiPA assay will be performed at the Baseline Visit to evaluate the prevalence of specific known HBV drug resistance mutations. FibroTest will be performed at Baseline and at Week 48. Plasma and serum for storage will be collected at each visit for possible bioanalytical and virological analyses (including resistance surveillance, HBsAg quantification, and adherence assessment). Plasma will be collected at each visit for tenofovir pharmacokinetic analysis.

**Test Product, Dose, and Mode of Administration:**	Tenofovir disoproxil fumarate 300 mg PO once daily
**Reference Therapy, Dose, and Mode of Administration:**	None
**Criteria for Evaluation:**	
Safety:	Adverse events and clinical laboratory tests will be collected at every visit and summarized through Week 48.
Efficacy:	The primary efficacy endpoint is HBV DNA < 400 copies/mL at Week 48.<br>Secondary endpoints for Week 48 include ALT normal; composite endpoint of HBV DNA < 400 copies/mL and ALT normal; change from Baseline in FibroTest value; HBeAg/HBsAg loss and seroconversion; HBV DNA < 169 copies/mL; composite endpoint of HBV DNA < 400 copies/mL, ALT normal and HBeAg loss; composite endpoint of HBV DNA < 400 copies/mL, ALT normal and HBe seroconversion; HBsAg loss and seroconversion; and development of drug resistance mutations.

**Statistical Methods:**	The proportion of subjects meeting the primary efficacy endpoint will be analyzed after the last subject reaches Week 48.<br>All continuous endpoints will be summarized using an 8-number summary (n, mean, standard deviation, median, Q1, Q3, min and max). All categorical endpoints will be summarized by number and percentage of subjects that meet the endpoint. Week 48 FibroTest results will be analyzed as a non-invasive marker of hepatic fibrosis.<br>For the analysis of the primary endpoint and categorical secondary endpoints, subjects discontinuing therapy prior to Week 48 for non-administrative reasons will be considered failures at (and all times after) the time of discontinuation of therapy. Subjects discontinuing therapy due to administrative reasons prior to Week 48 with plasma HBV DNA <400 copies/mL and no ongoing adverse event will be censored at the time of discontinuation.<br>**Sample Size:** Based on previous studies, a response rate of 84% is assumed for subjects receiving tenofovir DF.<br>The 95% confidence interval for this response rate with a sample size of 90 indicates that between 68 to 83 subjects are expected to achieve virologic response at Week 48.

This study will be conducted in accordance with the guidelines of Good Clinical Practices (GCPs) including archiving of essential documents.

GLOSSARY OF ABBREVIATIONS AND DEFINITION OF TERMS

°C	degrees Celsius
°F	degrees Fahrenheit
ACTG	AIDS Clinical Trials Group
AE	adverse event
ALT	alanine aminotransferase
ANC	absolute neutrophil count
AST	aspartate aminotransferase
β-HCG	beta-human chorionic gonadotropin
BMD	bone mineral density
CBC	complete blood count
CC_{50}	median concentration curve
CPK	creatine phosphokinase
CRF	Case Report Form(s)
CRO	Contract Research Organization
d4T	stavudine, Zerit®
dATP	deoxyadenosine triphosphate
$DAVG_{48}$	time-weighted average change from baseline through Week 48
dL	deciliter
DNA	deoxyribonucleic acid
DSPH	Drug Safety and Public Health
EC_{50}	median effective concentration
EEG	electroencephalogram
EFV	efavirenz
EMEA	European Medicines Evaluation Agency
ETV	entecavir
EU	European Union
EudraCT	European clinical trials database
FDA	(U.S.) Food and Drug Administration
FTC	emtricitabine, Emtriva™
g	gram(s)
GCP	Good Clinical Practice (Guidelines)
GGT	gamma-glutamyl transferase
HAV	hepatitis A virus
HBeAb	hepatitis B early antibody
HBeAg	hepatitis B early antigen
HBsAb	hepatitis B surface antibody

GLOSSARY OF ABBREVIATIONS AND DEFINITIONS OF TERMS (CONTINUED)

HBsAg	hepatitis B surface antigen
HBV	hepatitis B virus
HCC	hepatocellular carcinoma
HCV	hepatitis C virus
HDV	hepatitis D virus
HLGT	high level group term
HLT	high level term
HIV	human immunodeficiency virus
IC_{50}	median inhibitory concentration
ICH	International Conference on Harmonization
IEC	Independent Ethics Committee
IL-2	interleukin-2
IND	Investigational New Drug (Application)
INR	international normalized ratio
IRB	Institutional Review Board
IV	intravenous
kg	kilogram(s)
K_i	inhibition constant
L	liter
LAM	lamivudine, Epivir®, Zeffix®, 3TC
LAM^R	lamivudine resistant
LDH	lactate dehydrogenase
LFT	liver function test
LLN	lower limit of normal
LLOQ	lower limit of quantification
LLT	lower level term
LOD	limit of detection
m^2	square meter(s)
MedDRA	Medical Dictionary for Regulatory Activities
mEq	mlliequivalent(s)
mg	milligram(s)
min	minute(s)
mL	milliliter(s)
mm^3	cubic millimeter(s)
mmol	millimole(s)
ng	nanogram(s)

GLOSSARY OF ABBREVIATIONS AND DEFINITIONS OF TERMS (CONTINUED)

OBR	optimized background (antiretroviral) regimen
OLT	orthotopic liver transplant
PCR	polymerase chain reaction
PK	pharmacokinetic
PO	by mouth (per os)
PT	prothrombin time
PTH	parathyroid hormone
RBC	red blood cell
RNA	ribonucleic acid
SAE	serious adverse event
SCr	serum creatinine
SOC	system organ class
TDF	tenofovir DF, tenofovir disoproxil fumarate, Viread®
TFV-DP	tenofovir diphosphate
µg	microgram
µM	micromolar
ULN	upper limit of the normal range
WBC	white blood cell
WHV	woodchuck hepatitis virus

1. INTRODUCTION

1.1. Background

Chronic hepatitis B is a serious global health care problem and a major cause of chronic liver disease, cirrhosis, and hepatocellular carcinoma (HCC). Worldwide, approximately 350–400 million people have developed chronic hepatitis B, including approximately 1.2 million in the United States {10952}. Globally, approximately 1 million people die annually of complications of chronic hepatitis B {2331}, {10952}. In the United States, an estimated 5,000 people die each year of complications of HBV infection {3092}.

Despite the availability of HBV vaccine programs in many countries, new hepatitis B infections are still common, even in areas of low endemnicity. For example, approximately 70,000 people in the United States become acutely infected each year, according to estimates from the US Centers for Disease Control and Prevention {10690}. Following acute hepatitis B infection, approximately 5% of adults and up to 90% of children fail to produce an immune response adequate to clear the infection; these individuals become chronic carriers of the virus {3273}, {10665}.

Chronic hepatitis B has a broad clinical spectrum, ranging from asymptomatic, slowly progressive illness to severe, more rapidly progressive liver disease. Chronic hepatitis B may remain quiescent for many years. However, 15–40% of patients with chronic hepatitis B will ultimately develop serious liver disease and complications, such as cirrhosis, hepatic decompensation, and HCC {10952}. In patients with chronic hepatitis B, the annual probability of developing cirrhosis varies from 0.1% to 1.0%, depending on the duration of HBV replication, the severity of disease, and the presence of concomitant risk factors {2820}. Patients with chronic viral replication are at increased risk of complications and poor outcomes {10684}. Effective suppression of viral replication alters the course of the disease and decreases morbidity. However, the clinical benefit is lost if viral replication resumes as a result of emerging resistance mutations.

Chronic HBV infection disproportionately affects the Asian and Pacific Islander communities in the United States (‘Asian Americans’). These populations are more than twice as likely to be infected with HBV, as compared to Caucasian Americans, with a reported prevalence of at least 10% {12039}. One of the greatest health disparities is the incidence of HCC, with Asian Americans being 2.7 times more likely to develop HCC and 2.4 times more likely to die from liver cancer than Caucasian Americans {12038}, {12036}. Although liver cancer is relatively uncommon in the United States, it is the second highest cause of cancer mortality in Asian-American men, and the five-year survival rate is below 10% {12038}. A large proportion of Asian Americans who acquire HBV infection, particularly those infected early in life, are at high risk of developing chronic liver diseases, including liver cancer; up to 25% will ultimately die of liver disease.

Approximately 70% of Asians living in the US were born outside the US, most commonly in areas of high HBV endemnicity {12038}. The most common modes of HBV transmission in the Asian population are perinatal and early child-to-child transmission. Childhood and adult vaccination rates are often poor, and up to two-thirds of Asian Americans are unaware that they are infected with HBV, due to suboptimal screening programs {12039}. Those who are aware of the disease and/or are aware that they or a family member are infected with HBV are often unaware of the long-term risks associated with HBV infection and, subsequently, go untreated or occasionally seek out traditional, unproven therapies.

The goal of therapy for chronic hepatitis B infection in all populations is to maintain suppression of viral replication to prevent the emergence of complications, such as cirrhosis, decompensated liver disease, and HCC. Chen et al reported that sustained suppression of HBV replication by anti-HBV treatment reduces the risk of cirrhosis and liver cancer {10684}. Achieving durable viral suppression in chronic HBV infection usually requires long-term therapy, preferably utilizing potent regimens that limit the development of resistance.

Tenofovir disoproxil fumarate (tenofovir DF), an oral, once-daily nucleotide analog, has antiviral activity against both HBV and HIV and is approved for use in combination with other antiretroviral agents for the treatment of HIV infection in adults. The anti-HBV activity of tenofovir DF is equipotent to that of adefovir dipivoxil; however, the safety and tolerability profile of tenofovir DF allows the administration of higher doses {10665}. In an ongoing international comparative trial in adults (GS-US-174-0102), tenofovir DF was shown to be superior to adefovir dipivoxil for the treatment of HBeAg-negative/anti-HBe-positive (presumed precore mutant) chronic HBV infection. At 48 weeks, 70.8% of subjects in the tenofovir DF arm (n = 250) had a complete response (serum HBV DNA < 400 copies/mL and histologic improvement characterized by ≥ 2 point reduction in the Knodell necroinflammatory score), compared to 48.8% of subjects in the adefovir dipivoxil arm (n = 125; p < 0.001) {11363}. Both agents were well tolerated. A large ongoing international trial of similar design (GS-US-174-0103) is evaluating the efficacy and safety of tenofovir DF vs. adefovir dipivoxil in adults with HBeAg-positive chronic HBV infection. At 48 weeks, 66.5% of patients in the tenofovir DF arm (n = 176) had a complete response, compared to 12.2% in the adefovir dipivoxil arm (n = 90; p < 0.001) {11362}. A total of 75 Asian Americans received treatment with tenofovir DF in studies GS-US-174-0102 and GS-US-174-0103. Given the high rates of HBV-related morbidity and mortality in Asian Americans, it is important to collect additional efficacy, safety/tolerability and pharmacokinetic data in this patient population.

1.2. Tenofovir Disoproxil Fumarate (Tenofovir DF)

1.2.1. General Information

Tenofovir disoproxil fumarate (TDF; 9-[(R)-2-[[bis[[(isopropoxycarbonyl) oxy]methoxy]phosphi-nyl] methoxy]propyl] adenine fumarate (1:1); GS4331-05) is an oral prodrug (bisPOC-PMPA) of tenofovir (PMPA), an acyclic nucleoside phosphonate

(nucleotide) analogue of adenosine 5'-monophosphate. Tenofovir DF has activity against HBV and HIV and is indicated for use in combination with other antiretroviral agents in the treatment of HIV-1 infection in adults.

Tenofovir DF (300 mg once daily) was approved by the US FDA in October 2001, the European Commission in February 2002, and other markets worldwide for use in combination with other antiretroviral agents for the treatment of HIV-1 infection. Cumulative patient exposure to tenofovir DF since first marketing approval in the US on 26 October 2001 is estimated to be more than 1.5 million patient-years of treatment.

Tenofovir DF is being developed for the treatment of CHB in adults. Viread was approved in 2008 for the treatment of adults with CHB by regulatory authorities in the United States, Turkey, New Zealand, Australia and the European Union. The marketing application for Viread for the treatment of adults with CHB in Canada was submitted in October 2007 and is currently under review by the competent authority in Canada.

Further information is available in the Investigator's Brochure for tenofovir DF for HBV and the Package Insert for Viread®.

1.2.2. Pre-Clinical Pharmacology and Toxicology

1.2.2.1. Mechanism of Action

Tenofovir disoproxil fumarate (DF) is an oral prodrug of tenofovir (PMPA), an acyclic nucleotide (nucleoside monophosphate) analogue. Tenofovir DF is converted to tenofovir by serum esterases. Intracellularly, tenofovir is then converted through two phosphorylation reactions to its active phosphorylated anabolite, tenofovir diphosphate (TFV-DP), an obligate chain terminator {1574}. Tenofovir is efficiently anabolized to TFV-DP in human hepatic cells, and has a half-life of 95 hours in primary human hepatocytes {9266}. TFV-DP inhibits recombinant HBV polymerase with a K_i of 0.18 µM {9266}. TFV-DP inhibits viral polymerases by direct binding competition with the natural deoxyribonucleotide substrate (deoxyadenosine triphosphate - dATP) and, after incorporation into DNA, by DNA chain termination {1131}. TFV-DP is a very weak inhibitor of mammalian DNA polymerases α, β, and mitochondrial DNA polymerase γ {2516}, {1131}.

1.2.2.2. Anti-Hepatitis B Virus Activity

Tenofovir is a potent and selective inhibitor of HBV in vitro. The in vitro antiviral activity of tenofovir against an HBV laboratory strain was assessed in the HepG2 2.2.15 cell line. The EC_{50} values (50% effective concentration) for tenofovir were in the range of 0.14 to 1.5 µM, with CC_{50} (50% cytotoxicity concentration) values > 100 µM. In a parallel analysis, tenofovir inhibited various wild-type HBV clinical isolates with a comparable activity (Report No. PC-174-2005). It also inhibited replication of duck HBV (DHBV) in primary duck hepatocytes with an EC_{50} of 0.11 µM {10}. As observed with anti-HIV-1 activity, the

prodrug tenofovir DF had a 55-fold increased in vitro potency against HBV in comparison with tenofovir {9266}.

Tenofovir demonstrated anti-HBV activity similar to adefovir in the same cell lines. TFV-DP and adefovir diphosphate also showed comparable inhibition constants for HBV polymerase in enzyme assays. The EC_{50} values of lamivudine for HBV are approximately 10-fold lower than that of tenofovir in same cell lines. However, the inhibition constants of TFV-DP and lamivudine triphosphate for HBV polymerase were similar, suggesting that the intrinsic anti-HBV potency of tenofovir is similar to that of lamivudine. The higher EC_{50} values of tenofovir versus lamivudine in cell culture may be attributed to the lower cell permeability of the negatively-charged molecule of tenofovir.

Using HBV-expressing cell lines (Report Nos. PC-174-2006, PC-164-2004) {6870}, tenofovir demonstrated additive to slightly synergistic anti-HBV activity when tested in combination with nucleoside anti-HBV reverse transcriptase inhibitors. Specifically, tenofovir in combination with lamivudine, telbivudine, entecavir and adefovir, each produced additive anti-HBV activity (Report Nos. PC-174-2006, PC-164-2004) {6870}. The combination of tenofovir and emtricitabine demonstrated additive to slightly synergistic anti-HBV activity (Report No. PC-174-2006).

The anti-viral activity of tenofovir was also evaluated in woodchucks chronically infected with WHV in short-term (4 weeks) {8176} and long-term (48 weeks) antiviral efficacy studies (Report No. PC-174-2004). Oral administration of tenofovir DF at 0.5, 1.5, and 5.0 mg/kg of body weight/day for 4 weeks reduced serum viral load significantly, at 0.2 ($p < 0.01$), 1.1 ($p < 0.01$), and 1.5 $\log_{10}$ ($p < 0.05$) from the pretreatment levels {8176}. Oral administration of tenofovir DF at 15 mg/kg for 48 weeks resulted in a mean serum viral load reduction of 2.9 $\log_{10}$ copies/mL of WHV DNA. Administration of tenofovir DF in combination with lamivudine or emtricitabine for 48 weeks resulted in a mean serum viral load reduction of 5.8 and 6.1 $\log_{10}$, respectively. Over the 48-week dosing period, there was no evidence of toxicity in woodchucks treated with tenofovir DF, either alone or in combination (Report No. PC-174-2004).

1.2.2.3. Resistance

Hepatitis B virus resistance to tenofovir is yet to be identified. Recently, an HBV rtA194T mutation was reported to emerge in two patients receiving antiretroviral treatments including tenofovir DF, and an in vitro susceptibility assay indicated reduced susceptibility to tenofovir {8380}. The patients developed this mutation in the background of the rtL180M+rtM204V lamivudine-associated mutations, and its development was not clearly associated with HBV viral load rebound. To further characterize the rtA194T mutation, HBV with the rtA194T mutation alone or in combination with the rtL180M+rtM204V lamivudine resistance mutations were created and their in vitro tenofovir susceptibilities were tested in parallel with the parent wild-type and lamivudine-resistant strains. The data showed that rtA194T mutation alone had no significant effect on tenofovir susceptibility (1.5-fold increase in tenofovir EC_{50}). The rtA194T mutation in combination with the rtL180M+rtM204V

mutations led to a 2.4-fold increase in tenofovir EC_{50}, which was not significantly different than the 2.1-fold increase observed against virus containing the rtL180M+rtM204V mutations alone (Report No. PC-104-2012) {9266}. Various clinical studies have shown potent inhibition of lamivudine-resistant HBV by tenofovir DF, suggesting that a 2- to 3- fold change in in vitro susceptibility to tenofovir is not clinically relevant. Other mutations have not been reported to date.

Four major patterns of lamivudine resistance mutations, rtL180M/M204V, rtV173L/L180M/M204V, rtL180M/M204I, and rtM204I have been identified in patients who failed lamivudine therapy {6868}. In cell-based assays, HBV laboratory strains {6868}, {8381}, {8016}, {9266}, as well as those identified in clinical isolates {10916}, {10426}, {7060} expressing the rtV173L, rtL180M, and rtM204I/V mutations, showed a susceptibility to tenofovir ranging from 0.7 to 3.4-fold that of wild-type virus.

Resistance to adefovir dipivoxil is associated with development of the rtA181V and rtN236T mutations in the HBV reverse transcriptase. These mutations resulted in a 2.9- to 4.5-fold, respectively, reduced sensitivity to tenofovir in vitro {10926}, {9266}, {8016}, {7788}. The effects on susceptibility to tenofovir by other adefovir resistance associated mutations or combinations of mutations (rtA181T, rtA181V/rtN236T and rtA181T/rtN236T) were also evaluated using stably transfected cell lines. The results demonstrated a reduction in susceptibility to tenofovir of 1.5-, 10.0- and 3.0-fold each, respectively {10926}. The clinical significance of these in vitro results is currently unknown.

The mutations conferring resistance to entecavir have been identified as changes at rtI169T, rtT184G, rtS202I/G and rtM250V in combination with the pre-existing lamivudine resistance mutations {8683}, {10466}, {10804}. In vitro phenotypic analysis showed that the tested entecavir resistance mutations resulted in increased EC_{50} values for tenofovir ranging from 0.6- to 6.9-fold (Report No. PC-174-2003), {10946}, {10916}.

Virus harboring combinations of mutations resulting in resistance to both lamivudine and adefovir (rtV173L/L180M/A181V/N236T) were observed to emerge in a patient receiving combination therapy with these drugs. Virus obtained from this patient remained susceptible to inhibition by tenofovir in vitro {10425}, {10916}. Virus containing the rtL180M+rtM204V+rtN236T mutations created in a laboratory strain of HBV had greatly impaired replication capacity. These mutations resulted in a 4.4-fold increase in the tenofovir EC_{50} in vitro {8016}.

1.2.3. Clinical Trials of Tenofovir DF

1.2.3.1. Gilead Sciences Sponsored Clinical Trials of Tenofovir DF in HBV Mono-infected Subjects

There are currently six ongoing Gilead Sciences sponsored clinical trials that are designed to study the safety and antiviral activity of tenofovir DF as monotherapy or in combination with emtricitabine in subjects mono-infected with HBV.

1.2.3.1.1. Study GS-US-174-0102: A Randomized, Double-Blind, Controlled Evaluation of Tenofovir DF versus Adefovir Dipivoxil for the Treatment of Presumed Pre-core Mutant Chronic Hepatitis B

Study GS-US-174-0102 is a double-blind, randomized, Phase 3 study evaluating the safety and antiviral activity of tenofovir DF versus adefovir dipivoxil in subjects infected with presumed pre-core mutant chronic HBV. Key eligibility criteria for study entry include HBsAg positive for at least 6 months; HBeAg negative and anti-HBe positive; seronegative for HIV, HDV and HCV; adefovir dipivoxil and tenofovir DF naïve; and a Knodell necroinflammatory score of ≥ 3. Up to 40% of the subjects were allowed into the study with cirrhosis, i.e., a Knodell fibrosis score of 4, and/or with prior exposure to lamivudine. The study is currently ongoing; enrollment was completed in May 2006 with 375 subjects enrolled into the study. As of April 2007, all subjects completed the 48-week double-blind phase of the study. At 48 weeks, tenofovir DF was shown to be superior to adefovir dipivoxil, as 70.8% of subjects in the tenofovir DF arm (n = 250) had a complete response (serum HBV DNA < 400 copies/mL and histologic improvement characterized by ≥ 2 point reduction in the Knodell necroinflammatory score), compared to 48.8% of subjects in the adefovir dipivoxil arm (n = 125; p < 0.001) {11363}. Tenofovir DF treatment produced superior viral suppression compared with adefovir dipivoxil as indicated by the significantly greater percentage of subjects with HBV DNA below 400 copies/mL at Week 48 (93% in the tenofovir DF group vs. 63% in the adefovir dipivoxil group) (p < 0.001). Both drugs produced similar results with regard to histologic improvement (72% in the tenofovir DF group vs. 69% in the adefovir dipivoxil group) and normalization of ALT (76% vs. 77%, respectively) at the end of blinded treatment. No subject developed mutations associated with tenofovir resistance. At study entry, 17.2% of tenofovir DF-treated subjects had received more than 12 weeks of prior treatment with lamivudine or emtricitabine, and 16.8% had received prior treatment with interferon. At Week 48, all subjects were switched to open-label tenofovir DF, and the study has been extended to 5 years. Beginning at Week 72, subjects with a confirmed HBV DNA ≥ 400 copies/mL are eligible to receive emtricitabine/tenofovir DF combination therapy.

Tenofovir DF was well tolerated, with a safety profile comparable to adefovir dipivoxil and consistent with the known safety profile of tenofovir DF in patients with HIV infection. The most common AEs in both treatment groups were headache, nasopharyngitis, and back pain. Among the AEs observed in ≥ 5% of subjects, all except mild arthralgia (more common in the tenofovir DF group) were similarly frequent in the two treatment groups, and all except procedural pain (related to liver biopsies) were observed at a similar frequency in at least one of the randomized clinical studies in subjects with HIV infection described in the current prescribing information. The proportion of subjects experiencing Grade 2–4 AEs were similar between treatment groups.

The frequency of SAEs was similar in the two treatment groups, overall (4.8% in the tenofovir DF group and 5.6% in the adefovir dipivoxil group) and for each individual SAE. All clinical SAEs reported in the tenofovir DF group were either attributable to advanced

CHB disease or were observed in only 1 subject. Five tenofovir DF-treated subjects permanently discontinued treatment because of AEs (anorexia, bladder neoplasm, cervical carcinoma, fatigue, and feeling hot), while 2 adefovir dipivoxil-treated subjects permanently discontinued treatment because of AEs (liposarcoma and toxic myopathy).

There was no evidence of compromised renal function or bone events due to tenofovir DF. No subject had a confirmed 0.5-mg/dL increase from baseline in creatinine or a confirmed creatinine clearance below 50 mL/min. No fractures were pathological, and none were considered to be related to study medication.

The frequency of on-treatment ALT flares (> 10 × ULN and > 2 × baseline value) was low and similar in the two treatment groups (1% of subjects in each group). Treatment-associated ALT flares are best described as a result of increased immune response and cytotoxic activity associated with a rapid decline in viral replication. Flares were limited to increases in ALT and/or AST, and no subject experienced associated symptoms or decompensation.

No deaths were reported during the study period.

1.2.3.1.2. Study GS-US-174-0103: A Randomized, Double-Blind, Controlled Evaluation of Tenofovir DF versus Adefovir Dipivoxil for the Treatment of HBeAg Positive Chronic Hepatitis B

Study GS-US-174-0103 is a double-blind, randomized, Phase 3 study evaluating the safety and antiviral activity of tenofovir DF versus adefovir dipivoxil in subjects infected with HBeAg positive chronic HBV. Key eligibility criteria for study entry include HBsAg positive for at least 6 months; HBeAg positive; seronegative for HIV, HDV and HCV; adefovir dipivoxil and tenofovir DF naïve; and a Knodell necroinflammatory score of ≥ 3. Up to 40% of the subjects were allowed into the study with cirrhosis, i.e., a Knodell fibrosis score of 4. The study is currently ongoing; enrollment was completed in June 2006 with a total of 267 subjects enrolled. As of May 2007, all subjects completed the 48-week double-blind phase of the study. At 48 weeks, tenofovir DF was shown to be superior to adefovir dipivoxil, as 66.5% of patients in the tenofovir DF arm (n = 176) had a combined histologic and virologic response, compared to 12.2% in the adefovir dipivoxil arm (n = 90; p < 0.001) {11362}. A significantly greater proportion of subjects in the tenofovir DF group than in the adefovir dipivoxil group achieved HBV DNA below 400 copies/mL (76% vs. 13%, respectively) (p < 0.001).

Both drugs produced similar results for histologic response at Week 48. Tenofovir DF treatment produced a better biochemical effect as demonstrated by a significantly greater proportion of subjects with normal or normalized ALT at Week 48 (p = 0.018 and p = 0.032 respectively). A significantly greater proportion of tenofovir DF-treated subjects than adefovir dipivoxil-treated subjects had achieved HBsAg loss at Week 48 (p = 0.018). No subject developed mutations associated with tenofovir resistance. At study entry, 4.5% of tenofovir DF-treated subjects had received more than 12 weeks of prior treatment with lamivudine or emtricitabine, and 17.0% had received prior treatment with interferon. At

Week 48, all subjects were switched to open-label tenofovir DF, and the study has been extended to 5 years. Beginning at Week 72, subjects with a confirmed HBV DNA ≥ 400 copies/mL are eligible to receive emtricitabine/tenofovir DF combination therapy.

Tenofovir DF was well tolerated, with a safety profile consistent with the known safety profile of tenofovir DF as observed in patients with HIV-1 infection. The most frequent AEs were nausea, headache, nasopharyngitis, and fatigue. With the exception of mild nausea (more common in the tenofovir DF group), all AEs occurred at similar frequencies in the two treatment groups. Among the AEs observed at Grade 2 or greater severity in ≥ 3% of subjects, all were similarly frequent in the two treatment groups, and all except ALT flares were observed at a similar frequency in at least one of the randomized clinical studies in subjects with HIV infection.

The frequency of SAEs was similar in the two treatment groups, overall (8.5% in the tenofovir DF group and 7.8% in the adefovir dipivoxil group) and for each individual SAE. All clinical SAEs reported in the tenofovir DF group were either attributable to CHB disease or were observed in only 1 subject. No subject in the tenofovir DF group discontinued due to an AE.

There was no evidence of compromised renal function or bone events due to tenofovir DF. No subject had a confirmed 0.5-mg/dL increase from baseline in creatinine or a confirmed creatinine clearance below 50 mL/min. No cases of renal failure or severe renal impairment and no renal toxicity suggestive of proximal tubular dysfunction (Fanconi syndrome) were observed. No fractures were pathological, and none were considered to be related to study medication.

On-treatment ALT flares (> 10 × ULN and > 2 × baseline value) occurred at a similar frequency in the two treatment groups (< 5% of subjects in each group). Most flares occurred early in treatment accompanied by continued and profound decreases in HBV DNA, and were limited to increases in ALT and/or AST, and no subject experienced associated symptoms or decompensation. Five of the 8 tenofovir DF-treated subjects and 1 of the 3 adefovir dipivoxil-treated subjects with ALT flare seroconverted to anti-HBe.

No deaths were reported during the study period.

1.2.3.1.3. Study GS-US-174-0106: A Phase 2, Randomized, Double-Blind Study Exploring the Efficacy, Safety and Tolerability of Tenofovir Disoproxil Fumarate (DF) Monotherapy Versus Emtricitabine plus Tenofovir DF Fixed-Dose Combination Therapy in Subjects Currently Being Treated with Adefovir Dipivoxil for Chronic Hepatitis B and having Persistent Viral Replication

Study GS-US-174-0106 is a randomized, double-blind study evaluating the antiviral activity and safety of tenofovir DF monotherapy versus emtricitabine/tenofovir DF combination therapy in subjects infected with chronic HBV who are undergoing treatment with adefovir

dipivoxil and who have persistent viral replication. Key eligibility criteria for study entry include HBsAg positive for at least 6 months; current receipt of adefovir dipivoxil treatment (for ≥ 24 weeks but ≤ 96 weeks) with HBV DNA > 10^3 copies/mL; seronegative for HIV, HDV and HCV; and tenofovir DF- and emtricitabine/tenofovir DF-naïve. The study is 168 weeks in duration, and subjects with confirmed HBV DNA ≥ 400 copies/mL on or at any time after 24 weeks on study drug are required to switch to open-label emtricitibine/tenofovir DF combination or discontinue from the trial and begin commercially available therapy.The study is fully enrolled (105 subjects randomized and treated) and ongoing.

1.2.3.1.4. Study GS-US-174-0108: A Phase 2, Double-Blind, Multi-center, Randomized Study Comparing Tenofovir Disoproxil Fumarate, Emtricitabine Plus Tenofovir Disoproxil Fumarate, and Entecavir in the Treatment of Chronic Hepatitis B Subjects with Decompensated Liver Disease and in the Prevention of Hepatitis B Recurrence Post-Transplantation

Study GS-US-174-0108 is a double-blind, multi-center, randomized, Phase 2 study to compare the safety and antiviral activity of tenofovir DF, emtricitabine/tenofovir DF, and entecavir in the treatment of chronic HBV in subjects with decompensated liver disease. Subjects will be randomized 2:2:1 to tenofovir DF:emtricitabine/tenofovir DF:entecavir. Key eligibility criteria for study entry include a Child-Pugh-Turcotte score of 7–12 (inclusive); HBV DNA ≥ 10^3 copies/mL; serum ALT < 10 × ULN; seronegative for HIV, HCV, and HDV; and no history of variceal bleeding, hepatorenal syndrome, Grade 3 or Grade 4 hepatic encephalopathy, or spontaneous bacterial peritonitis within 60 days of the screening visit. The study is 168 weeks in duration. Open-label emtricitabine/tenofovir DF combination therapy will be offered to subjects who experience (a) a decrease in HBV DNA from baseline of < 2 $\log_{10}$ copies/mL and HBV DNA > 10,000 copies/mL (or > 1000 copies/mL if entered with HBV DNA < 10,000 copies/mL) at Week 8 or (b) a virologic breakthrough (≥ 1 $\log_{10}$ copies/mL increase from nadir in HBV DNA on 2 consecutive determinations) or HBV DNA > 400 copies/mL after Week 24. The study is currently ongoing; enrollment has been completed with a total of 112 subjects enrolled.

1.2.3.1.5. Study GS-US-203-0101: A Randomized, Double-Blind Study Evaluating Tenofovir Disoproxil Fumarate (DF) Monotherapy Versus the Combination of Emtricitabine and Tenofovir DF for the Treatment of Chronic Hepatitis B

Study GS-US-203-0101 is a double-blind, multi-center, randomized study to compare the safety and antiviral activity of tenofovir DF versus emtricitabine/tenofovir DF in the treatment of chronic HBV in 100 subjects with high viral load (HBV DNA ≥ 10^8 copies/mL) and normal ALT values. Subjects will be randomized 1:1 to tenofovir DF or emtricitabine/tenofovir DF. Key eligibility criteria for study entry include HBV DNA ≥ 10^8 copies/mL; serum ALT ≤ ULN; and seronegative for HIV, HCV, and HDV. The study is 96 weeks in duration. The study is currently ongoing.

1.2.3.1.6. Study GS-US-203-0107: A Phase 2, Open-Label Randomized Study to Evaluate the Efficacy and Safety of the Combination Product, Emtricitabine/Tenofovir Disoproxil Fumarate in the Presence or Absence of Hepatitis B Immunoglobulin (HBIG) in Preventing Recurrence of Chronic Hepatitis B (CHB) Post-Orthotopic Liver Transplant (OLT)

Study GS-US-203-107 is a Phase 2, open-label, randomized study to evaluate the safety and efficacy of the fixed-dose combination emtricitabine/tenofovir DF in the presence or absence of hepatitis B immunoglobulin in preventing recurrence of CHB after orthotopic liver transplantation. The study is underway, with a planned sample size of 25 subjects in each of the two treatment groups.

1.2.3.2. Investigator-Sponsored Reports of Tenofovir DF in HBV Mono-Infected and HBV and HIV-1 Co-infected Subjects

Summary data derived from a number of investigator-sponsored studies are outlined in Appendix 7; these reports provide evidence for the anti-HBV activity of tenofovir DF either as monotherapy or in combination with either lamivudine or emtricitabine. Anti-HBV activity of tenofovir DF has been reported in subjects with chronic hepatitis B mono-infection, including orthotopic liver transplant (OLT) recipients, and subjects with renal allografts. Published data reported for HIV-1 and HBV co-infected subjects with HIV and HBV given tenofovir DF monotherapy or tenofovir DF plus lamivudine (or emtricitabine) combination therapy consistently show a decrease in serum HBV DNA in the range of $\geq 4 \log_{10}$ copies/mL. A large number of subjects, both with and without comorbidities, harbored lamivudine-refractory HBV, as indicated by genotypic identification of YMDD mutations at study entry or HBV viremia while on lamivudine treatment. The magnitude of the HBV DNA response to tenofovir DF treatment appeared to be independent of the presence or absence of mutations in the YMDD motif.

Although the majority of studies of tenofovir DF did not include a comparator, there were two controlled studies, Study ACTG 5127 and a study by van Bommel and colleagues. Both favorably demonstrated the anti-HBV activity of tenofovir DF, as compared to adefovir dipivoxil. Study ACTG 5127 evaluated HBV/HIV co-infected subjects on antiretroviral therapy, with or without resistance to lamivudine, who were randomized to double-blind tenofovir DF 300 mg daily (n = 27) or adefovir dipivoxil 10 mg daily (n = 25) {10408}. The time-weighted average change in serum HBV DNA from baseline after 48 weeks was –4.44 $\log_{10}$ copies/mL for subjects on tenofovir DF and –3.21 $\log_{10}$ copies/mL for subjects on adefovir dipivoxil ($p < 0.05$; Roche Amplicor Cobas PCR assay, lower limit of quantification [LLOQ] 200 copies/mL). The study by van Bommel and colleagues evaluated 53 consecutive subjects with lamivudine-resistant chronic HBV infection who were treated either with tenofovir DF (n = 35) or adefovir dipivoxil (n = 18). While all subjects on tenofovir DF were suppressed < 400 copies/mL at Week 48, suppression below LLOQ occurred in 44% of subjects on adefovir dipivoxil ($p = 0.001$) (mean HBV DNA decrease from baseline was –5.5

and −2.8 $\log_{10}$ copies/mL at Week 48 in the tenofovir DF and adefovir dipivoxil arms, respectively [$p < 0.001$]) {7213}.

There have also been published reports in subjects who, after having undergone OLT, received tenofovir DF either as monotherapy or in combination with lamivudine {7833}, {6842}, {7877}, {7735}. In most cases, tenofovir DF was added post transplantation following chronic HBV recurrence, i.e., breakthrough of HBV DNA. No adverse events associated with tenofovir DF were noted, and subjects generally responded favorably, with a decrease in HBV DNA and ALT. Norris and colleagues reported favorable outcomes in two HIV-1/HBV co-infected subjects who after undergoing OLT, immediately received tenofovir DF in combination with lamivudine and hepatitis B immune globulin (HBIG) to prevent chronic HBV recurrence. Subjects were reported as alive without HBV recurrence at 6 and 25 months post transplant {7833}.

1.2.4. Summary of Safety of Tenofovir DF in HIV-1 Mono-Infected and HBV and HIV-1 Co-Infected Subjects

Overall, tenofovir DF 300 mg has been shown to be well tolerated in subjects with HIV-1 infection, with no correlation between dose and the overall incidence of AEs, serious adverse events (SAEs), laboratory toxicities, or the frequency of any specific AE or laboratory toxicity (dose ranging study GS-98-902).

Safety data reported for subjects co-infected with HBV and HIV are derived from substudies of HIV clinical trials. In controlled clinical trials (GS-98-902, GS-99-903, GS-99-907, and GS-99-910), subjects co-infected with HBV and HIV did not exhibit any unexpected AEs or laboratory abnormalities, compared either to the overall HIV population (GS-99-910) or compared to placebo or an active control (GS-98-908, GS-99-907, GS-98-903). Additionally, although the data are limited, investigator-initiated studies have not reported any suspect AEs and, in fact, have shown tenofovir DF 300 mg to be well tolerated in both liver and kidney transplant subjects either co-infected with HIV/HBV or mono-infected with chronic hepatitis B.

Consistent with other antivirals in the treatment of chronic HBV, the primary safety concern with tenofovir DF is the risk of post-treatment exacerbation of hepatitis (“flares”) once the drug is discontinued. Subjects should be closely monitored with both clinical and laboratory follow-up for at least several months after stopping tenofovir DF treatment. There is insufficient experience to determine whether re-initiation of tenofovir DF alters the course of the exacerbation of hepatitis. In patients with advanced liver disease or cirrhosis, treatment discontinuation is not recommended since post-treatment exacerbation of hepatitis may lead to hepatic decompensation.

Other precautions noted with the use of tenofovir DF in subjects infected with HIV-1 include lactic acidosis, renal impairment and changes in bone mineral density.

The preclinical and clinical data suggest that the risk of occurrence of lactic acidosis, a class effect of nucleoside analogues, is low for tenofovir DF. However, as tenofovir is structurally related to nucleoside analogues, this risk cannot be excluded. Lactic acidosis and severe hepatomegaly with steatosis, including fatal cases, have been reported with the use of nucleoside analogues, including tenofovir DF, in combination with other antiretrovirals. Treatment with tenofovir DF should be suspended in any subject who develops clinical or laboratory findings suggestive of lactic acidosis or pronounced hepatotoxicity.

Renal impairment, including cases of acute renal failure and Fanconi syndrome, has been reported in association with the use of tenofovir DF. In clinical trials of HIV-infected subjects, the incidence of clinically relevant renal events was low and generally mild (Grade 1) in treatment-naïve subjects treated with a regimen containing tenofovir DF for up to 144 weeks and in treatment-experienced subjects treated for up to 4 years. Elevated creatinine, renal insufficiency, renal failure, hypophosphatemia and Fanconi syndrome have been reported with the use of tenofovir DF in clinical practice. The majority of these cases occurred in patients with underlying renal disease or tenofovir DF in combination with nephrotoxic agents. There was no evidence of compromised renal function in pivotal studies of tenofovir DF in patients with chronic hepatitis B. No cases of renal failure or severe renal impairment and no renal toxicity suggestive of proximal tubular dysfunction (Fanconi syndrome) were observed. It is recommended that creatinine clearance be calculated in all patients prior to initiating therapy and as clinically appropriate during therapy with tenofovir DF. Routine monitoring of calculated creatinine clearance and serum phosphorus should be performed in patients at risk for renal impairment. Tenofovir DF should be avoided with concurrent or recent use of a nephrotoxic agent.

Bone safety has been assessed during prolonged administration of tenofovir DF in long-term HIV clinical trials. The clinical relevance of the changes in surrogate bone biomarkers and bone mineral density (BMD) in treatment-naive subjects is not known; changes in BMD in Study GS-99-903 were non-progressive after Week 48. Clinically relevant bone abnormalities have not been observed in long-term clinical studies (> 3 years). Osteomalacia in association with proximal renal tubulopathy has been identified during postmarketing surveillance, manifesting as bone pain and infrequently contributing to fractures. In pivotal studies of tenofovir DF in patients with chronic hepatitis B, there was no evidence of bone events due to treatment with tenofovir DF. No fractures were considered pathological, and none were considered to be related to study medication. Bone monitoring should be considered for patients who have a history of pathologic bone fracture or are at risk for osteopenia. Although the effect of supplementation with calcium and vitamin D was not studied, such supplementation may be beneficial for all patients. If bone abnormalities are suspected, then appropriate consultation should be obtained.

There is now extensive postmarking exposure to tenofovir DF (alone and in combination) in patients with HIV-1 infection, with a cumulative estimate of more than 1.5 million patient-years of exposure since first marketing approval in the US on 26 October 2001. Postmarketing experience is summarized in the Tenofovir DF Investigator's Brochure and in the product label.

1.3. Rationale for the Current Study and Dose Selection

The aim of treatment for chronic hepatitis B infection is to maintain suppression of viral replication to prevent the development of complications. Achieving this goal requires potent, durable, well-tolerated, patient-friendly antiviral regimens. Clinical data in adults with HBeAg-positive and HBeAg-negative/anti-HBe-positive chronic HBV infection demonstrate the efficacy and safety of tenofovir DF 300 mg daily for the treatment of chronic HBV disease. Tenofovir DF is a promising agent for use in Asian-American subjects with chronic HBV because of its favorable efficacy, safety and tolerability profiles. The pharmacokinetic profile of tenofovir DF allows convenient, once-daily dosing, which can facilitate adherence. Tenofovir DF was approved in 2008 for the treatment of CHB in adults in the United States, Turkey, New Zealand, Australia and the European Union.

It is important to further study the efficacy, safety and tolerability of anti-HBV treatments in Asian Americans, given the disproportionately high rate of HBV infection and associated morbidity and mortality in this population. Asian Americans are twice as likely to be infected with HBV as Caucasian Americans and are 2.7 times more likely to develop HCC and 2.4 times more likely to die from liver cancer than Caucasian Americans {12038}, {12036}. A phase IV study evaluating tenofovir DF in Asian-American adults will collect additional safety and efficacy data on this agent for the treatment of CHB in this patient population. In addition, the study will help to further elucidate the pharmacokinetic and resistance profiles of tenofovir DF. Through their participation, study subjects will help generate additional information regarding the use of tenofovir DF in Asian-Americans with CHB infection.

1.3.1. Rationale for Tenofovir DF Dose Selection

The selection of the 300 mg dose for tenofovir DF for use in adults is based upon the following rationale: (1) The 300 mg dose has been demonstrated to be the optimal dose of tenofovir DF for the treatment of HIV-1 infection; the K_i of tenofovir against HIV-1 reverse transcriptase (0.02-1.6 μM) is similar to the K_i against HBV polymerase (0.18 μM); (2) The safety profile of tenofovir DF 300 mg once daily has been well characterized in patients with HIV infection, and tenofovir DF 300 mg once daily has been shown to be safe in that patient population; (3) Reducing the dose of tenofovir DF may lead to an increased risk of the emergence of resistance as documented with other antivirals; (4) In the dose-ranging study GS-98-902, the safety profile of tenofovir DF 300 mg once daily was not different from the safety profiles of lower doses of tenofovir DF (75 mg and 150 mg); and (5) The safety of the 300 mg dose is similar in HIV-infected patients with or without co-infection with chronic hepatitis B, as supported by study GS-99-910. Further, in the GS-US-174-0102 and GS-US-174-0103 studies, the 300 mg dose of tenofovir DF was shown to be an effective dose for treatment of chronic hepatitis B infection in adults with HBeAg negative/anti-HBe-positive disease and HBeAg-positive disease, respectively, and the 300 mg oral dose was approved in 2008 for the treatment of CHB in adults. All adult subjects in this substudy will receive tenofovir DF as a 300 mg tablet, administered once daily without regard to food. Monitoring of tenofovir plasma concentrations at each study visit will be useful for a pharmacokinetic analysis of tenofovir in in Asian-American adults.

1.3.2. Rationale for FibroTest

Liver biopsy is the gold standard for evaluating HBV-related inflammation and fibrosis and for measuring treatment-related effects on liver histology. Although liver biopsy is generally well tolerated, sampling error and complications may occur. This has led clinicians and researchers to search for non-invasive markers of liver fibrosis. FibroTest is a non-invasive marker of liver fibrosis that is widely used in Europe. The FibroTest value is determined via incorporation of several laboratory tests (total bilirubin, α_2-macroglobulin, apolipoprotein A_1, haptoglobin and gamma glutamyl-transferase) into a propriety algorithm that generates a score between 0 and 1 that correlates with a patient's degree of liver fibrosis {12037}. FibroTest has been evaluated in a variety of liver diseases, including chronic hepatitis B, and has been validated in an Asian population {12043}. The diagnostic value of FibroTest is best for extreme stages (no fibrosis, cirrhosis), but is lower for discriminating intermediate fibrosis stages {12037}. In the current study, change in FibroTest value from Baseline to Week 48 will be measured to evaluate treatment-related changes in fibrosis.

2. OBJECTIVES

The primary objective of this study is:

- To evaluate the antiviral efficacy of tenofovir DF 300 mg once daily in Asian-American adults with chronic hepatitis B (CHB) infection

Secondary objectives are:

- To evaluate the safety and tolerability of tenofovir DF in Asian-American adults with CHB infection
- To evaluate the biochemical and serological responses to tenofovir DF in Asian-American adults with CHB infection
- To evaluate incidence of drug resistance mutations
- To evaluate change from baseline to Week 48 in FibroTest, a non-invasive biomarker of liver fibrosis

3. STUDY DESIGN

This is a Phase IV, open-label, single-arm 48-week study to evaluate the antiviral efficacy, safety and tolerability of tenofovir DF in Asian-American adults with chronic HBV infection. Ninety (90) tenofovir DF-naïve subjects with HBV DNA ≥ 10^4 copies/mL at screening and ALT > ULN and ≤ 10 × ULN at screening or within the past 12 months (as documented in the subject's medical record) will receive treatment with open-label tenofovir DF 300 mg PO once daily.

Subjects must be naive to tenofovir DF, but could have taken < 12 weeks of oral anti-HBV nucleoside/nucleotide therapy, with the last dose ≥ 16 weeks prior to screening. Subjects must have discontinued interferon ≥ 6 months prior to screening. Enrollment will be capped at 45 males to ensure adequate participation by Asian-American females. No more than 10 subjects may be enrolled at a single site without prior written approval from Gilead Sciences. The participation of each subject will end after 48 weeks of treatment, at which time subjects may be transitioned to commercially-available HBV drugs (not provided by the study) or enrolled in a patient assistance program. Subjects who discontinue tenofovir DF at the end of 48 weeks and do not immediately initiate commercially-available HBV treatment must be followed for an additional 6 months or until they initiate commercially-available HBV treatment, whichever occurs first.

At screening, adult Asian-American subjects (18–75 years of age) with chronic HBV infection (HBsAg positive for at least 6 months) with HBV DNA ≥ 10^4 copies/mL; ALT > ULN and ≤ 10 × ULN at screening or within the past 12 months, as documented in the subject's medical record; creatinine clearance ≥ 60 mL/min/1.73 m^2 and normal platelet count will be eligible for the study. Subjects must be naive to tenofovir DF, but could have taken < 12 weeks of oral anti-HBV nucleoside/nucleotide therapy, with the last dose ≥ 16 weeks prior to screening; subjects must have discontinued interferon ≥ 6 months prior to screening. Subjects must be without serological evidence of co-infection with HIV, HCV or HDV. Subjects with a history of significant bone disease, decompensated liver disease, evidence of hepatocellular carcinoma (i.e., α-fetoprotein > 50 ng/mL), and pregnant or breast-feeding females will not be eligible for the study. Subjects > 40 years of age with α-fetoprotein ≤ 50 ng/mL must undergo a hepatic ultrasound or CT scan to rule out hepatocellular carcinoma.

Plasma HBV DNA levels, laboratory analyses (serum chemistry, liver tests, hematology, and urinalysis), pregnancy test (for females of child bearing potential), vital signs, weight, adverse events and concomitant medications will be measured or assessed at Screening, Baseline, and Weeks 4, 8, 16, 24, 32, 40, and 48. Height will be measured at the Screening and Baseline Visits. HBV serology (HBsAg, HBeAg, and reflex HBeAb and HBsAb if Ag negative) will be conducted at Screening, Baseline and every 16 weeks through Week 48. Complete physical examinations will be performed at Screening, Baseline, and Weeks 24 and 48; symptom-directed physical examinations will be performed at all other visits.

Determination of HBV viral genotype (A-H) will be performed at Baseline for all subjects. Genotypic analysis of the HBV polymerase by di-deoxy sequencing will be conducted at Baseline for all subjects, and attempted for all viremic subjects (HBV DNA ≥ 400 copies/mL) at Week 48. In addition, theINNOLiPA assay will be performed at the Baseline Visit to evaluate the prevalence of specific known HBV drug resistance mutations. FibroTest will be performed at Baseline and at Week 48. Plasma and serum for storage will be collected at each visit for possible bioanalytical and virological analyses (including resistance surveillance, HBsAg quantification, and adherence assessment). Plasma will be collected at each visit for possible tenofovir population pharmacokinetic analysis.

4. SUBJECT POPULATION

4.1. Number of Subjects and Subject Selection

A total of 90 tenofovir DF-naïve Asian-American adults with chronic HBV will receive open-label tenofovir DF 300 mg PO once daily for 48 weeks. Enrollment of male subjects will be capped at 45 males to ensure adequate representation of Asian-American females in the study. No more than 10 subjects may be enrolled at a single single without prior written authorization from Gilead Sciences.

4.2. Inclusion Criteria

Subjects must meet *all* of the following inclusion criteria to be eligible for participation in this study.

- Male or female
- Asian-American, defined as a person of self-reported Asian ancestry who is residing in the US
- 18 through 75 years of age, inclusive
- Documented chronic HBV infection, defined as positive serum HBsAg ≥ 6 months
- HBV DNA ≥ 10^4 copies/mL (PCR method)
- ALT > ULN and ≤ 10 × ULN at screening or within the past 12 months prior to screening (must be definitively documented in the subject's medical record)
- Willing and able to provide written informed consent
- Negative serum β-HCG pregnancy test (females of child-bearing potential)
- Estimated glomerular filtration rate (creatinine clearance) ≥ 60 mL/min/1.73m^2 by the Cockcroft-Gault equation:

$$\frac{\text{(140-age in years) (body weight [kg])}}{\text{(72) (serum creatinine [mg/dl])}}$$

[Note: multiply estimated rate by 0.85 for women; use actual body weight]

- Adequate hematologic function (absolute neutrophil count ≥ 1,500/mm^3; hemoglobin ≥ 10.0 g/dL)
- No prior tenofovir DF therapy; subjects may have taken < 12 weeks of oral anti-HBV therapy, with the last dose ≥ 16 weeks prior to screening; subjects may have received prior interferon, but must have discontinued interferon therapy ≥ 6 months prior to screening

4.3. Exclusion Criteria

Subjects who meet *any* of the following exclusion criteria are not to be enrolled in this study.

- Pregnant women, women who are breast feeding or who believe they may wish to become pregnant during the course of the study.
- Males and females of reproductive potential who are not willing to use an effective method of contraception during the study. For males, condoms should be used and for females, a barrier contraception method should be used in combination with one other form of contraception.
- Decompensated liver disease defined as direct (conjugated) bilirubin > 1.2 × ULN, PT > 1.2 × ULN, platelets < 150,000/mm^3, or serum albumin < 3.5 g/dL
- Prior history of clinical hepatic decompensation (e.g., ascites, jaundice, encephalopathy) or variceal hemorrhage
- Receipt of prior tenofovir DF treatment
- Receipt of ≥ 12 weeks of oral anti-HBV nucleoside/nucleotide therapy, or receipt of ANY oral anti-HBV treatment < 16 weeks prior to screening
- Receipt of interferon (pegylated or not) therapy within 6 months of the Screening Visit
- α-fetoprotein > 50 ng/mL
- Evidence of hepatocellular carcinoma (HCC)
- Co-infection with HIV, HCV, or HDV
- History of significant renal disease (e.g., nephrotic syndrome, renal dysgenesis, polycystic kidney disease, congenital nephrosis, acute tubular necrosis, other renal disease)
- History of significant bone disease (e.g., osteomalacia, chronic osteomyelitis, osteogenesis imperfecta, osteochrondroses, multiple bone fractures)
- Significant cardiovascular, pulmonary or neurological disease
- Evidence of a gastrointestinal malabsorption syndrome that may interfere with absorption of orally administered medications
- History of solid organ or bone marrow transplantation
- Ongoing therapy with any of the following:

  — Nephrotoxic agents

    - Parenteral aminoglycoside antibiotics (e.g., gentamicin, tobramycin, amikacin)
    - Cidofovir
    - Cisplatin
    - Foscarnet
    - IV amphotericin B
    - IV pentamidine
    - Oral or IV ganciclovir
    - Cyclosporine
    - Tacrolimus
    - IV vancomycin
    - Chronic daily non-steroidal anti-inflammatory drug therapy

— Competitors of renal excretion (e.g., probenecid)
— Systemic chemotherapeutic agents
— Systemic corticosteroids
— Interleukin-2 (IL-2) and other immunomodulating agents
— Investigational agents (except with the expressed approval of the Sponsor)

Administration of any of the above medications must be discontinued at least 30 days prior to the Baseline Visit and for the duration of the study period.

- Known hypersensitivity to the study drugs, the metabolites or formulation excipients
- Any other condition (including alcohol or substance abuse) or prior therapy that, in the opinion of the Investigator, would make the subject unsuitable for the study or unable to comply with dosing requirements

4.4. Study-Specific Tolerance and Inclusion/Exclusion Criteria

At the discretion of the Gilead Sciences Medical Monitor, up to a 10% variance may be allowed for eligibility criteria on a case per case basis, for eligibility time windows.

5. STUDY DRUGS

5.1. Treatment Assignment

This is an open-label, single-arm, non-randomized study. Approximately 90 tenofovir DF-naïve subjects will receive open-label tenofovir DF 300 mg PO once daily. Subjects will be assigned a screening number at the time of consent. Once eligibility has been confirmed, subjects will be assigned a subject number by Gilead Sciences after receipt and review of the Patient Eligibility Worksheet to ensure that enrollment of male subjects is capped at 45 (to allow adequate representation of females in the study). A maximum of 10 subjects may be enrolled at each study site, unless the site has received prior written approval from Gilead Sciences to enroll > 10 subjects.

All Baseline tests and procedures must be completed prior to the administration of the first dose of study drugs. Initiation of treatment with study drugs should take place within 24 hours of the Baseline Visit.

During the 48-week study, open-label tenofovir DF 300 mg will be provided to the study sites in unnumbered bottles by Gilead Sciences.

5.2. Description and Handling of Study Drug

5.2.1. Formulation

Tenofovir DF tablets are light blue, almond-shaped, film-coated tablets containing 300 mg of tenofovir DF. Each tablet contains the following inactive ingredients: microcrystalline cellulose, lactose monohydrate, pregelatinized starch, croscarmellose sodium, and magnesium stearate. The tenofovir DF tablets are film-coated to mask taste. Each tablet is film-coated with a mixture of lactose monohydrate, hypromellose (hydroxypropyl methylcellulose), glycerol triacetate, titanium dioxide, and indigo carmine aluminum lake.

5.2.2. Packaging and Labeling

The study drug (tenofovir DF) is packaged in white, high-density polyethylene (HDPE) bottles with a white child-resistant cap. There are 30 tablets per bottle. Each bottle also contains silica gel as a dessicant to protect the product from humidity and fiber packing to protect the product during handling and shipping.

During the 48-week study, subjects will be provided with sufficient supplies for 4 to 8 weeks of dosing (subjects will receive one bottle at the Baseline Visit and two bottles at subsequent visits through Week 40). At a minimum, each bottle will be labeled with a lot number, the protocol number, administration instructions, storage instructions, expiration date, and Sponsor name and address. Additional information will be included according to the requirements of the protocol and local law.

5.2.3. Storage and Handling

Tenofovir DF tablets should be stored at 25°C (77°F); excursions are permitted to 15°–30°C (59°–86°F).

To ensure product stability, study drugs should not be dispensed in a container other than the one supplied.

5.3. Dosage and Administration of Study Drugs

Subjects will receive the following study treatment in an open-label fashion:

Tenofovir DF 300 mg PO once daily

Subjects will be instructed to take one tablet by mouth daily. Study drugs can be taken without regard to food.

The participation of each subject will end after 48 weeks of treatment, at which time subjects may be transitioned to commercially-available HBV drugs (not provided by the study) or enrolled in a patient assistance program. Subjects who discontinue tenofovir DF at the end of 48 weeks and do not immediately initiate commercially-available HBV treatment must be followed for an additional 6 months or until they initiate commercially-available HBV treatment, whichever occurs first.

Active study subjects will be dispensed study drug at the Baseline Visit and through the end of the study (until each subject reaches Week 48). Subjects will be instructed to return empty containers as well as unused study medication in the original container at each study visit. The investigator will be responsible for maintaining accurate records for all study drug and study drug bottles dispensed and returned. The inventory must be available for inspection by the study monitor. Study medication supplies, including partially used or empty bottles, must be accounted for and the dispensing logs must be verified by the study monitor prior to destruction or return.

5.4. Prior and Concomitant Medications

5.4.1. Prior to Study Entry

Refer to Exclusion Criteria in Section 4.3. Subjects must not have received oral anti-HBV nucleoside/nucleotide therapy < 16 weeks prior to screening for this study. Subjects must not have received interferon within six months prior to screening for this study.

5.4.2. During the Study

Use of the following medications is prohibited while subjects are on study drug:

- Antiviral agents with anti-HBV activity, including lamivudine, emtricitabine, adefovir, entecavir, telbivudine, clevudine, or others
- Interferon and pegylated interferon
- Nephrotoxic agents such as aminoglycoside antibiotics, cidofovir, cisplain, foscarnet, IV amphotericin B, IV pentamidine, ganciclovir, cyclosporine, tacrolimus, chronic daily non-steroidal anti-inflammatory drugs, or other agents with significant nephrotoxic potential
- Hepatotoxic agents such as anabolic steroids, isoniazid, itraconazole, ketoconazole, lovastatin, rifabutin, rifampin, simvastatin, and other agents with significant hepatotoxic potential
- Competitors of renal excretion, such as probenecid
- Systemic chemotherapeutic agents
- Interleukin-2 [IL-2]) and other immunomodulating agents
- Systemic corticosteroids
- Investigational agents, except with written approval of the Sponsor

Should subjects need to start treatment with any excluded concomitant medication, the Sponsor must be consulted prior to initiation of the new medication. In instances where an excluded medication is initiated prior to discussion with the Sponsor, the Investigator must notify the Sponsor as soon as s/he is aware of the use of the excluded medication.

All concomitant medications, including vitamin supplements, herbal remedies and hormonal contraception, must be recorded in the appropriate section of the Case Report Forms.

6. STUDY PROCEDURES

Study procedures to be conducted for each subject enrolled in the study are presented in tabular form in Appendix 2 and in the text that follows. Additional information on the study procedures will be provided in written materials by the Contract Research Organization (CRO).

Any deviation from protocol procedures should be noted in the Case Report Forms (CRFs), and the Sponsor or CRO should be notified.

All protocol-specified laboratory tests on blood and urine samples must be performed at the selected central laboratory. Refer to the appropriate central laboratory instruction manuals for information on sample collection and shipment of all required study samples.

6.1. Subject Enrollment and Treatment Assignment

Each study candidate must sign an Informed Consent Form prior to the conduct of any screening procedures, in accordance with regulatory and local Ethics Committee requirements. Screening evaluations will be used to determine the eligibility of each candidate for study enrollment. Candidates who fail to meet eligibility criteria by screening evaluations may be re-screened once ≥ 30 days after the initial screen if there is a reasonable expectation that the candidate will be eligible after repeat screening.

6.1.1. Screening Visit

The following assessments and procedures will be performed and recorded on CRFs at the initial Screening Visit:

- Written informed consent from subject
- Medical history, including hepatitis B history and treatment history
- Complete physical examination (excluding breast, anorectal, and urogenital exams), vital signs (temperature, blood pressure, heart rate, respiratory rate), body weight, and height
- Blood samples for:
    - Hematology (complete blood count [CBC] with differential and platelet count)
    - Serum chemistry and liver function tests, including albumin, alkaline phosphatase, AST, ALT, total bilirubin (reflex to direct [conjugated] bilirubin if total bilirubin > 1.5 × ULN), bicarbonate, BUN, calcium, chloride, CPK, creatinine (and calculated creatinine clearance), glucose, LDH, magnesium, phosphorus, potassium, sodium, uric acid, and amylase (reflex lipase testing if total amylase is ≥ 1.5 × ULN)
    - Prothrombin time (PT), international normalized ratio (INR)
    - Hepatitis B serology (HBeAg and HBsAg; reflex HBeAb and HBsAb if Ag negative)
    - Plasma HBV DNA (PCR method)
    - HIV, HCV and HDV serology

- Serum β-HCG pregnancy test (females of child-bearing potential)
- α-fetoprotein (AFP)
- Serum and plasma storage (for potential bioanalytical and virologic assays, including resistance surveillance, HBsAg quantification and adherence assessment)

- Hepatic ultrasound or CT scan (subjects > 40 years of age with α-fetoprotein ≤ 50 ng/mL)
- Urinalysis (protein, glucose, blood)
- Review of AEs and concomitant medications
- Review of all inclusion and exclusion criteria

Subjects meeting all of the inclusion criteria and none of the exclusion criteria will return to the clinic within 6 weeks (42 days) of the initial Screening Visit for entry into the study. All candidates who meet screening requirements will be enrolled into the study until the planned enrollment of 45 male subjects and 45 female subjects has been met.

6.1.2. Baseline Assessments

The Baseline Visit should occur within 6 weeks (42 days) days of the initial Screening Visit. Once eligibility has been confirmed, subjects will be assigned a subject number by Gilead Sciences after receipt and review of the Patient Eligibility Worksheet to ensure that enrollment of male subjects is capped at 45 (to allow adequate representation of females in the study). A maximum of 10 subjects may be enrolled at each study site, unless the site has received prior written approval from Gilead Sciences to enroll > 10 subjects.

All Baseline tests and procedures must be completed prior to the receipt of the first dose of study drugs. Subjects will be dispensed study medication at the Baseline Visit. Study medication should be initiated within 24 hours of the Baseline Visit.

The following assessments and procedures will be performed and recorded on CRFs at the Baseline Visit:

- Changes in medical history since screening
- Complete physical examination (excluding breast, anorectal, and urogenital exams), vital signs, body weight and height
- Blood samples for:

  - Hematology (CBC with differential and platelet count)
  - Serum chemistry and liver function tests, including albumin, alkaline phosphatase, AST, ALT, total bilirubin (reflex to direct [conjugated] bilirubin if total bilirubin > 1.5 × ULN), bicarbonate, BUN, calcium, chloride, CPK, creatinine (and calculated creatinine clearance), glucose, LDH, magnesium, phosphorus, potassium, sodium, uric acid, and amylase (reflex lipase testing if total amylase is ≥ 1.5 × ULN)
  - Prothrombin time (PT), international normalized ratio (INR)
  - Hepatitis B serology (HBeAg and HBsAg; reflex HBeAb and HBsAb if Ag negative)

- Plasma HBV DNA (PCR method)
- Serum β-HCG pregnancy test (females of child-bearing potential)
- Serum for HBV viral genotyping (A–H) and resistance surveillance (sequence analysis of the HBV polymerase through di-deoxy sequencing)
- Serum for HBV drug resistance test (INNOLiPA assay)
- Plasma for determination of tenofovir concentration
- Serum and plasma for storage (for potential bioanalytical and virologic assays, including HBsAg quantification and adherence assessment)
- FibroTest analytes: α_2-macroglobulin, apolipoproteinA_1, haptoglobin, gamma glutamyl-transferase (total bilirubin is included in the chemistry/liver test panel)

- Urinalysis (protein, glucose, blood)
- Review of AEs and concomitant medications
- Study drug dispensing and instructions on appropriate dosing and administration

6.2. Treatment Assessments

6.2.1. Week 4, 8, 16, 24, 32 and 40 Assessments

The following evaluations will be performed at Weeks 4, 8, 16, 24, 32, and 40, unless otherwise specified. Study visits are to be completed ± 3 days of the protocol-specified visit date, based on the Baseline Visit.

The following assessments and procedures will be performed and recorded on CRFs:

- Symptom-directed physical examination, including vital signs and body weight (complete physical examination, excluding breast, anorectal, and urogenital exams, at Week 24 Visit only)
- Blood samples for:

  - Hematology (CBC with differential and platelet count)
  - Serum chemistry and liver function tests, including albumin, alkaline phosphatase, AST, ALT, total bilirubin (reflex to direct [conjugated] bilirubin if total bilirubin > 1.5 × ULN), bicarbonate, BUN, calcium, chloride, CPK, creatinine (and calculated creatinine clearance), glucose, LDH, magnesium, phosphorus, potassium, sodium, uric acid, and amylase (reflex lipase testing if total amylase is ≥ 1.5 × ULN)
  - Hepatitis B serology (HBeAg and HBsAg; reflex HBeAb and HBsAb if Ag negative; Weeks 16 and 32 only)
  - Plasma HBV DNA (PCR method)
  - Plasma for determination of tenofovir concentration
  - Serum and plasma for storage (for potential bioanalytical and virological assays, including resistance surveillance, HBsAg quantification and adherence assessment)

- Urine samples for:
  - Urinalysis (protein, glucose, blood)
  - Urine pregnancy test (females of child-bearing potential; positive urine pregnancy test will be immediately confirmed with a serum pregnancy test)
- Review of AEs and changes in concomitant medications
- Retrieval of study medication and assessment of medication adherence
- Study drug dispensing and instructions on appropriate dosing and administration

By the Week 32 Visit, a mechanism must be identified for the subject to access commercially-available anti-HBV treatment at the conclusion of the 48-week study.

6.2.2. Week 48 Assessments

Study Visit for Week 48 should be completed ± 3 days of the protocol-specified visit date, based on the Baseline Visit. The following assessments and procedures will be performed and recorded on CRFs:

- Complete physical examination (excluding breast, anorectal, and urogenital exams), vital signs and body weight
- Blood samples for:
  - Hematology (CBC with differential and platelet count)
  - Serum chemistry and liver function tests, including albumin, alkaline phosphatase, AST, ALT, total bilirubin (reflex to direct [conjugated] bilirubin if total bilirubin > 1.5 × ULN), bicarbonate, BUN, calcium, chloride, CPK, creatinine (and calculated creatinine clearance), glucose, LDH, magnesium, phosphorus, potassium, sodium, uric acid, and amylase (reflex lipase testing if total amylase is ≥ 1.5 × ULN)
  - Hepatitis B serology (HBeAg and HBsAg; reflex HBeAb and HBsAb if Ag negative)
  - Plasma HBV DNA (PCR method)
  - Serum β-HCG pregnancy test (females of child-bearing potential)
  - Serum for resistance surveillance (sequence analysis of HBV polymerase through di-deoxy sequencing)
  - Plasma for determination of tenofovir concentration
  - Serum and plasma for storage (for potential bioanalytical and virologic assays, including resistance surveillance, HBsAg quantification and adherence assessment)
  - FibroTest analytes: α_2-macroglobulin, apolipoproteinA_1, haptoglobin, gamma glutamyl-transferase (total bilirubin is included in the chemistry/liver test panel)
- Urinalysis (protein, glucose, blood)
- Review of AEs and concomitant medications
- Retrieval of study medication and assessment of medication adherence

Subjects who discontinue the study prior to the Week 48 Visit will complete all Week 48 assessments and procedures at an Early Study Drug Discontinuation Visit, to be completed within 72 hours of last dose of study drug. Subsequent off-study therapy, if any, is at the discretion of the subject/physician and will not be provided by Gilead Sciences. Subjects who have received at least one dose of study drug and permanently discontinue study drug will be followed for 24 weeks off treatment or up to initiation of active treatment, whichever occurs first.

6.3. Post-Treatment/Treatment-Free Follow-up Assessments

6.3.1. Six-Month Post-Treatment Follow-Up Assessments

Subjects who have received at least one dose of study drug and permanently discontinue study drug will be followed for 24 weeks off treatment or up to initiation of active treatment, whichever occurs first. **Subjects with known cirrhosis or bridging fibrosis should not discontinue anti-HBV treatment, due to risk of hepatic decompensation if post-treatment exacerbation occurs off medication.**

For those subjects who remain off treatment, the following evaluations are to be completed every 4 weeks through the Week 24 Follow-Up Visit (Follow-Up Weeks 4, 8, 12, 16, 20 and 24):

- Symptom-directed physical examination, including vital signs and body weight
- Blood samples for:
  - Serum chemistry and liver function tests, including albumin, alkaline phosphatase, AST, ALT, total bilirubin (reflex to direct [conjugated] bilirubin if total bilirubin $> 1.5 \times$ ULN), bicarbonate, BUN, calcium, chloride, CPK, creatinine (and calculated creatinine clearance), glucose, LDH, magnesium, phosphorus, potassium, sodium, uric acid, and amylase (reflex lipase testing if total amylase is $\geq 1.5 \times$ ULN)
  - Hepatitis B serology (HBeAg and HBsAg; reflex HBeAb and HBsAb if Ag negative) (Follow-Up Week 24 only)
  - Plasma HBV DNA (PCR method)
  - Serum and plasma for storage (for potential bioanalytical and virological assays, including resistance surveillance, and HBsAg quantification)
  - Prothrombin time (PT), international normalized ratio (INR) (reflex test in case of post-treatment exacerbation of hepatitis)
- Review of AEs and concomitant medications

Subjects experiencing post-treatment exacerbation of hepatitis during the 24-week post-treatment period should be followed weekly until their ALT levels return to Grade 2 or baseline, as described in the Toxicity Management section of the protocol (Section 7.6.)

6.3.2. Assessments for Premature Discontinuation from the Study (Early Study Drug Discontinuation Visit)

Subjects who permanently discontinue study drug prior to the end of the study will be asked to return to the clinic within 72 hours of stopping study drug for an Early Study Drug Discontinuation Visit. Subjects who discontinue the study prior to the Week 48 Visit will complete all Week 48 assessments and procedures (see Section 6.2.2. above). Subjects who have received at least one dose of study drug and permanently discontinue study drug will be followed with visits every 4 weeks for 24 weeks off treatment or up to initiation of active treatment, whichever occurs first. Subsequent off-study therapy, if any, is at the discretion of the subject/physician and will not be provided by Gilead Sciences.

Subjects experiencing post-treatment exacerbation of hepatitis during the 24-week post-treatment period should be followed weekly until their ALT levels return to Grade 2 or baseline, as described in the Toxicity Management section of the protocol (Section 7.6.)

6.4. Tenofovir Plasma Concentration Determination

For all subjects, blood (plasma) samples will be collected at each visit for analysis of concentration of tenofovir in the blood. At the time each blood sample is collected, the following information will be recorded:

- The date and time of last dose taken
- Whether last dose was taken with or without food
- The date and time of blood draw following last dose taken

All plasma samples will be sent to the central laboratory. Additional instructions regarding plasma collection and processing are provided in Appendix 4.

6.5. Serum and Plasma for Storage

Additional blood (plasma and serum) samples will be collected at each visit for long-term storage and possible future testing, including possible bioanalytical analysis (e.g., serum chemistry retest) and virologic analysis (e.g., HBV DNA retest, resistance surveillance). No human genetic testing will be performed. At the conclusion of this study, these samples may be retained in storage for a period of up to 15 years.

6.6. Resistance Surveillance

The objectives of the resistance surveillance are: (1) to identify mutations in the HBV polymerase gene from HBV subject isolates that are potentially associated with virological resistance to tenofovir DF, (2) to determine the correlation of the effects of these mutations to the clinical response to tenofovir DF therapy, (3) to determine whether these mutations alter antiviral susceptibility to tenofovir DF using in vitro HBV replication assays, (4) to evaluate the cross resistance profile of these mutations, and (5) to estimate the prevalence of specific known HBV drug resistance mutations at baseline.

Sequence analysis of the HBV polymerase using di-deoxy sequencing will be conducted at Baseline for all subjects, and attempted for all viremic subjects (HBV DNA ≥ 400 copies/mL) at Week 48, as well as at the Early Discontinuation Visit, if applicable. An additional analysis will be conducted at Baseline using the INNOLiPA assay to evaluate the prevalence of specific known HBV drug resistance mutations. Serum for storage will be collected at every visit for possible virologic analyses (see Section 6.5.).

6.7. Criteria for Discontinuation of Study Treatment

Study medication may be discontinued in the following instances:

- Intercurrent illness that would, in the judgment of the investigator, affect assessments of clinical status to a significant degree
- Unacceptable toxicity, as defined in the toxicity management section of the protocol, or toxicity that, in the judgment of the investigator, compromises the ability to continue study-specific procedures or is considered to not be in the subject's best interest
- Subject requests to discontinue for any reason
- Subject non-adherence
- Pregnancy during the study
- Discontinuation of the study at the request of Gilead Sciences, regulatory agency or an IRB/IEC

7. ADVERSE EVENTS AND TOXICITY MANAGEMENT

7.1. Adverse Events

An adverse event (AE) is any untoward medical occurrence in a clinical investigation subject administered a medicinal product and which does not necessarily have a causal relationship with this treatment. An AE can therefore be any unfavorable and unintended sign, symptom, or disease temporally associated with the use of a medicinal product, whether or not considered related to the medicinal product.

- AEs may also include the following: Pre-or post-treatment complications that occur as a result of protocol mandated procedures (e.g., invasive procedures such as venipuncture, biopsy) during or after screening (before the administration of study drug).
- Any pre-existing condition that increases in severity, or changes in nature during or as a consequence of the study drug phase of a human clinical trial, will also be considered an AE.
- Complications and terminations of pregnancy (see Section 7.7. for additional information)

All AEs that occur after the subject consents to participate in the study and throughout the duration of the study, including the follow-up off-study medication period should be recorded as an AE.

An AE does not include the following:

- Medical or surgical procedures (e.g., surgery, endoscopy, tooth extraction, transfusion) performed; the condition that leads to the procedure is an adverse event
- Pre-existing diseases or conditions or laboratory abnormalities present or detected before the screening visit that do not worsen
- Situations where an untoward medical occurrence has not occurred (e.g., hospitalization for elective surgery, social and/or convenience admissions)
- Overdose without clinical sequelae, unless the subject is hospitalized (see Section 7.3.1., *Overdose).*
- Any medical condition or clinically significant laboratory abnormality with an onset date before the consent form is signed and not related to a protocol-associated procedure is not an AE. It is considered to be pre-existing and should be documented on the medical history eCRF.
- Uncomplicated pregnancy
- An induced elective abortion to terminate a pregnancy without medical reason.

7.2. Assessment of Adverse Events

All AEs will be assessed by the investigator and recorded on the AE CRF page. The AE entry should indicate whether or not the AE was serious, the start date (AE onset), the stop

date (date of AE resolution), whether or not the AE was related to study drug or to a study procedure, the action taken with study drug due to the AE, and the severity of the AE.

The relationship to study drug therapy should be assessed using clinical judgment and the following definitions:

- **No**: Evidence exists that the adverse event has an etiology other than the study drug. For SAEs, an alternative causality must be provided (e.g., pre-existing condition, underlying disease, intercurrent illness, or concomitant medication).
- **Yes**: A temporal relationship exists between the AE onset and administration of the study drug that cannot be readily explained by the subject's clinical state or concomitant therapies. Furthermore, the AE appears with some degree of certainty to be related, based on the known therapeutic and pharmacologic actions or adverse event profile of the study drug. In case of cessation or reduction of the dose, the AE abates or resolves and reappears upon rechallenge.

It should be emphasized that ineffective treatment should not be considered as causally related in the context of adverse event reporting.

The relationship to study procedures (e.g., invasive procedures such as venipuncture or biopsy) should be assessed using the following definitions:

- **No:** Evidence exists that the adverse event has an etiology other than the study procedure.
- **Yes:** The adverse event occurred as a result of protocol-mandated procedures such as venipuncture, biopsy or diagnostic tests.

7.3. Serious Adverse Events

A **serious adverse event** (SAE) is defined as follows:

Any adverse drug experience occurring at any dose that results in any of the following outcomes:

- Death
- Life-threatening situation (subject is at **immediate** risk of death)
- In-patient hospitalization or prolongation of existing hospitalization (excluding those for study therapy or placement of an indwelling catheter, unless associated with other SAEs)
- Persistent or significant disability/incapacity
- Congenital anomaly/birth defect in the offspring of a subject who received study drug
- Other: medically significant events that may not be immediately life-threatening or result in death or hospitalization, but based upon appropriate medical and scientific judgment, may jeopardize the subject or may require medical or surgical intervention to prevent one of the outcomes listed above

Examples of such events are as follows:

- Intensive treatment in an emergency room or at home for allergic bronchospasm
- Blood dyscrasias or convulsions that do not result in hospitalization
- Development of drug dependency or drug abuse

For the purposes of this study, in addition to the above criteria, the following must be reported as an SAE:

- Serum ALT > 2 × baseline and > 10 × ULN, with or without associated symptoms.
- Confirmed ALT elevation (defined as 1-grade shift or 2 × previous value) associated with confirmed changes outside of the normal range in other laboratory parameters suggestive of worsening hepatic function (total bilirubin ≥ 2 mg/dL above baseline, abnormal PT ≥ 2 seconds or INR ≥ 0.5 over baseline, abnormal serum albumin ≥ 1 g/dL below baseline or elevated serum lactate levels (if available), defined as 2 × ULN per the Adult AIDS Clinical Trials Group (AACTG) guidelines).
- Any clinical manifestations of hepatic decompensation (variceal bleeding, hepatic encephalopathy, or worsening of ascites requiring diuretics or paracentesis).

Clarification of Serious Adverse Events

- Death is an outcome of an AE, and not an adverse event in itself. In reports of death due to “Disease Progression,” where no other information is provided, the death will be assumed to have resulted from progression of the disease being treated with the study drug(s).
- All deaths, regardless of cause or relationship, must be reported for subjects on study and for deaths occurring within 30 days of last study drug dose or within 30 days of last study evaluation, whichever is longer.
- The subject may not have been on study drug at the occurrence of the event. Dosing may have been given as treatment cycles or interrupted temporarily before the onset of the SAE, but may have contributed to the event.
- “Life-threatening” means that the subject was at immediate risk of death from the event as it occurred. This does not include an event that might have led to death if it had occurred with greater severity.
- Complications that occur during hospitalizations are AEs. If a complication prolongs the hospitalization, it is a SAE.
- “In-patient hospitalization” means the subject has been formally admitted to a hospital for medical reasons, for any length of time. This may or may not be overnight. It does not include presentation and care within an emergency department.
- The investigator should attempt to establish a diagnosis of the event on the basis of signs, symptoms and/or other clinical information. In such cases, the diagnosis should be documented as the AE and/or SAE and not the individual signs/symptoms.

A distinction should be drawn between seriousness and severity of AEs. An AE that is assessed as Grade 4 (potentially life-threatening) should not be confused with an SAE. Severity is a category utilized for rating the intensity of an event; and both AEs and SAEs can be assessed as Grade 4. An event is defined as “serious” when it meets one of the predefined outcomes described above in Section 7.3.

7.3.1. Overdose

Any accidental or intentional overdose (any increase in frequency or dosage of study medication that exceeds what is mandated by the protocol), misuse or abuse of blinded study medication or Gilead study drug, as well as any Gilead product taken as a concomitant medication, whether suspected or confirmed, and whether or not associated with an adverse experience, must be reported on the Overdose eCRF, and the CRO Safety Representative must be notified.

Overdose will be considered an SAE only if any of the seriousness criteria are met. Any clinical sequelae in association with the overdose should be reported as an AE (as outlined in Section 7.1.) or SAE (as outlined in Section 7.3.). Details of signs or symptoms, clinical management and outcome should be reported, if available.

7.4. Serious Adverse Event Reporting Requirements

7.4.1. All Serious Adverse Events

Gilead is required to expedite to worldwide regulatory authorities reports of Serious Adverse Events, Serious Adverse Drug Reactions or Suspected Unexpected Serious Adverse Reactions (SUSARs) in line with relevant legislation, including the European Commission Clinical Trials Directive (2001/20/EC); therefore, Gilead (or the CRO on the behalf of Gilead) must be notified immediately regarding the occurrence of any SAE or SADR that occurs after the subject consents to participate in the study, including SAEs/SADRs resulting from protocol-associated procedures as defined in relevant legislation including 2001/20/EC, performed from screening onwards. The procedures for reporting all SAEs, regardless of causal relationship, are as follows:

- Record the SAE on the AE CRF and complete the “Serious Adverse Event Report” form.
- Fax or email the SAE form to the attention of the CRO within 24 hours of the investigator’s knowledge of the event. Contact information is listed below.

Chiltern Safety Representative:	Name: Jennifer Headrick, Senior Pharmacovigilance Officer<br>Phone: 1-888-723-2445<br>**Fax: 1-888-726-8416**<br>**Email: Jennifer.Headrick@Chiltern.com**

- For fatal or life-threatening events, also fax copies of hospital case reports, autopsy report, and other documents when requested and applicable.

Gilead Sciences may request additional information from the investigator to ensure the timely completion of accurate safety reports.

The investigator must take all therapeutic measures necessary for resolution of the SAE. Any medications necessary for treatment of the SAE must be recorded onto the concomitant medication section of the subject's CRF.

Follow-up of adverse events will continue through the last day on study (including the follow-up off-study medication period of the study) and/or until the investigator and/or Gilead Sciences determine that the subject's condition is stable. Gilead Sciences may request that certain adverse events be followed until resolution.

7.4.2. Investigator and Sponsor Reporting Requirements for SAEs

An event may qualify for expedited reporting to worldwide regulatory authorities if it is a Serious Adverse Event, Serious Adverse Drug Reaction or Suspected Unexpected Serious Adverse Reaction (SUSAR) in line with relevant legislation, including the European Commission Clinical Trials Directive (2001/20/EC). Expectedness of SAEs will be determined by Gilead using the reference safety information specified in the US Package Insert. All investigators will receive a safety letter notifying them of relevant SUSAR reports. In accordance with the European Commission Directive 2001/20/EC, Gilead will notify the relevant Ethics Committees in concerned Member States of applicable SUSARs as individual notifications or through a periodic line listing.

The investigator should notify the IRB or IEC as soon as is practical, of serious events in writing where this is required by local regulatory authorities, and in accordance with the local institutional policy.

7.4.3. Post-Study Reporting Requirements

All deaths, regardless of cause or relationship, must be reported for subjects on study and for all deaths occurring within 30 days of last study drug dose.

Investigators are not obligated to actively seek out SAEs beyond the follow-up period for subjects. However, if the investigator learns of an AE or SAE occurring after the completion/termination visit and the event is deemed by the investigator to be probably or possible related to the use of study drugs, he/she should promptly document and report the event to Gilead Sciences.

7.5. Clinical Laboratory Abnormalities and Other Abnormal Assessments as Adverse Events or Serious Adverse Events

Laboratory abnormalities are usually not recorded as AEs or SAEs. However, laboratory abnormalities (e.g. clinical chemistry, hematology, urinalysis) independent of the underlying medical condition that require medical or surgical intervention or lead to study drug interruption or discontinuation must be recorded as an AE, as well as an SAE, if applicable. In addition, laboratory or other abnormal assessments (e.g., electrocardiogram, X-rays, vital signs) that are associated with signs and/or symptoms must be recorded as an AE or SAE if they meet the definition of an AE (or SAE) as described in Sections 7.1 and 7.3. If the laboratory abnormality is part of a syndrome, record the syndrome or diagnosis.

Severity should be recorded and graded according to the GSI Grading Scale for Severity of Adverse Events and Laboratory Abnormalities (Appendix 6). For adverse events associated with laboratory abnormalities, the event should be graded on the basis of the clinical severity in the context of the underlying conditions; this may or may not be in agreement with the grading of the laboratory abnormality.

7.6. Toxicity Management (Other Than On-Treatment ALT Flare, Post-Treatment Exacerbation of Hepatitis, or Serum Creatinine Elevation/Decreased Creatinine Clearance)

All clinical toxicities and/or abnormal laboratory findings should be investigated for etiology and graded according to the uniform guidelines detailed in Appendix 6. The Gilead Sciences Medical Monitor is available for consultation on all medical and toxicity-related issues and may be contacted as shown below:

Gilead Sciences Medical Monitor:	Name:	Elizabeth Fagan, MD
	Phone:	(650) 522-4286
	Cell Phone:	(301) 524-6032 (650) 504-6703
	Fax:	(919) 493-5925 (650) 522-5473
	E-mail:	elizabeth.fagan@gilead.com

- All clinical and clinically significant laboratory toxicities will be managed according to uniform guidelines detailed in Appendix 5.
- Grade 3 and 4 clinically significant laboratory abnormalities should be confirmed by repeat testing within 3 calendar days of receipt of results and before study drug discontinuation, unless such a delay is not consistent with good medical practice.
- Clinical events and clinically significant laboratory abnormalities will be graded according to the GSI Grading Scale for Severity of Adverse Events and Laboratory Abnormalities (Appendix 6).

- When restarting study drug following resolution of the adverse event, the study drug should be restarted at full dose or modified dose that is dependent upon discussion with the Gilead Sciences Medical Monitor.
- Any recurrence of the study drug-related Grade 3 or 4 clinical or clinically significant laboratory adverse event following dose interruption mandates permanent discontinuation of study drug.
- Any questions regarding toxicity management should be directed to the Gilead Sciences Medical Monitor.

7.6.1. Grades 1 and 2 Laboratory Abnormality or Clinical Event

- Continue study drug at the discretion of the investigator.

7.6.2. Grade 3 Laboratory Abnormality or Clinical Event

- For Grade 3 clinically significant laboratory abnormality or clinical event, study drug may be continued if the event is considered to be unrelated to study drug.
- For a Grade 3 clinical event, or clinically significant laboratory abnormality confirmed by repeat testing, that is considered to be related to study drug, study drug should be withheld until the toxicity returns to ≤ Grade 2.
- If a laboratory abnormality recurs to ≥ Grade 3 following rechallenge with study drug and is considered related to study drug, then study drug should be permanently discontinued and the subject managed according to local practice. Recurrence of laboratory abnormalities considered unrelated to study drug may not require permanent discontinuation.

7.6.3. Grade 4 Laboratory Abnormality or Clinical Event

- For a Grade 4 clinical event or clinically significant Grade 4 laboratory abnormality confirmed by repeat testing that is considered related to study drug, study drug should be permanently discontinued and the subject managed according to local practice. The subject should be followed as clinically indicated until the laboratory abnormality returns to baseline or is otherwise explained, whichever occurs first. A clinically significant Grade 4 laboratory abnormality that is not confirmed by repeat testing should be managed according to the algorithm for the new toxicity grade.

Study drug may be continued without dose interruption for a clinically non-significant Grade 4 laboratory abnormality (e.g., Grade 4 CPK after strenuous exercise, or triglyceride elevation that is nonfasting or that can be medically managed) or a clinical event considered unrelated to study drug.

7.6.4. Management of Elevated Serum Creatinine and Decreased Creatinine Clearance

- Serum creatinine values ≥ 0.5 mg/dL above baseline should be confirmed by repeat testing within 3 calendar days of receipt of results and before study drug discontinuation, unless such a delay is not consistent with good medical practice.
- For Grade 1 serum creatinine elevations ≥ 0.5 mg/dL above baseline, subjects may continue all study drug, but it is recommended that subjects be monitored weekly until the serum creatinine returns to the original baseline value or ≤ 0.3 mg/dL from baseline.
- All study drug should be permanently discontinued in the event that repeat testing of serum creatinine confirms a Grade 2 value. The subject should be followed weekly until the serum creatinine reaches within 0.3 mg/dL of the baseline value.
- Subjects whose calculated creatinine clearance decreases to < 50 mL/min should have this value confirmed within 72 hours (a 24-hour creatinine clearance can be measured in lieu of a calculated clearance). If the creatinine clearance is confirmed to be < 50 but ≥ 30 mL/min, the dosing interval for tenofovir DF should be adjusted to every 48 hours. If the creatinine clearance is confirmed to be < 30 mL/min, study drug should be discontinued.

7.6.5. On-Treatment ALT Flare and Post-Treatment Exacerbation of Hepatitis Management

For subjects with <u>known</u> bridging fibrosis or cirrhosis, study drug discontinuation with <u>treatment-free</u> follow-up is contraindicated due to the potential risk of exacerbation of hepatitis in the setting of low hepatic reserve which could lead to decompensation. Subjects with bridging fibrosis or cirrhosis should be placed on commercially available HBV therapy following study drug discontinuation.

On-Treatment ALT Flare and Post-Treatment Exacerbation of Hepatitis are defined as:

Serum ALT > 2 × baseline <u>and</u> > 10 × ULN, with or without associated symptoms OR

- Confirmed ALT elevation (defined as 1-grade shift or 2 × previous value) associated with confirmed changes outside of the normal range in other laboratory parameters suggestive of worsening hepatic function (total bilirubin ≥ 2 mg/dL above baseline, abnormal PT ≥ 2 seconds or INR ≥ 0.5 over baseline, abnormal serum albumin ≥ 1 g/dL below baseline or elevated serum lactate levels (if available), defined as 2 × ULN per the Adult AIDS Clinical Trials Group (AACTG) guidelines).

7.6.5.1. Management of ALT Flare in Subjects Receiving Study Medication

If laboratory results indicate (1) elevation of ALT > 2 × baseline and > 10 × ULN OR (2) abnormal laboratory parameters suggestive of worsening hepatic function (abnormal bilirubin ≥ 2mg/dL above baseline, abnormal PT ≥ 2 sec above baseline, INR ≥ 0.5 above baseline, abnormal albumin ≥ 1 g/dL decrease from baseline or elevated serum lactate

levels > 2 × ULN along with any ALT elevation (i.e., grade shift or 2 × previous value), the following is recommended:

- Schedule the subject to return to the clinic as soon as possible and ideally no later than one week after the initial labs were drawn. During the visit, perform a clinical assessment of the subject. The assessment should include a physical examination and evaluation of the subject's mental status.
- Draw blood samples, request lactate testing and send for confirmation of elevated serum transaminases (ALT/AST), total bilirubin and PT/INR, and albumin. [Note: If, in the investigator's judgment, the central laboratory cannot provide adequate turn around time, the confirmation test may also be performed at a local laboratory. However, the central laboratory results are considered definitive].

If the elevations are confirmed, request the central clinical laboratory to conduct reflex testing for serum HBV DNA, HBV serology (HBeAg, HBeAb, and HBsAg), HDV, HAV IgM, and HCV serology.

Based on the results of the confirmatory tests, the following treatment modifications are recommended:

Elevated Liver Enzymes, Normal Bilirubin, Normal PT/INT, Normal Albumin, Normal Lactate

If ALT and/or AST levels are elevated (i.e., > 2 × baseline and > 10 × ULN) but total bilirubin and PT/INR, albumin and lactate are normal, the subject may remain on study medication and should be monitored every week until ALT/AST return to normal or baseline levels. During monitoring:

- If ALT/AST levels decline within 4 weeks, the subject should remain on study and return to the clinic per protocol.
- If after 4 weeks of monitoring, ALT/AST values remain elevated (e.g., > 2 × baseline and > 10 × ULN) or have worsened, with bilirubin ≤ 2.5 × ULN, PT ≤ 1.5 × ULN, or abnormal albumin or lactate levels, the investigator should consult with the Gilead Medical Monitor.
- If ALT remains > 2 × baseline and > 10 × ULN and the bilirubin or PT values are confirmed at > 2.5 × ULN or > 1.5 × ULN, respectively, the investigator should consider discontinuing study medication and initiating alternative HBV therapy (see below). However, prior to initiating alternative therapy, medical management of the subject should be discussed with the Gilead Medical Monitor. (Note: Once a subject has started alternative therapy, s/he must be discontinued from the study.)

Elevated Liver Enzymes, Elevated Bilirubin and PT/INR (> Grade 2) Symptomatic Elevated Lactate (> 2 × ULN) or Asymptomatic Elevated Lactate (> 4 × ULN)

If ALT/AST values are elevated (i.e., > 2 × baseline and > 10 × ULN) and bilirubin or PT values are confirmed at > 2.5 × ULN or > 1.5 × ULN , respectively, or lactate levels are increased (symptomatic and > 2 × ULN or asymptomatic and > 4 × ULN) the investigator should consider discontinuing study medication and initiating alternative HBV treatment. The subject must be monitored weekly for as long as enzyme levels and bilirubin and PT/INR remain elevated or above baseline values. Refer to Appendix 8 for specific guidelines for the management of symptomatic and asymptomatic hyperlactatemia.

- If the ALT/AST levels return to the baseline level and/or Grade 2 or lower during the first 8 weeks of monitoring, study medication may be resumed.
- If the ALT/AST levels, bilirubin, PT/INR or lactate levels remain elevated up through Week 8 or deteriorate at any point, the investigator should consult with the Gilead Medical Monitor.

7.6.5.2. Management of Exacerbation of Hepatitis in Subjects Who Have Discontinued Study Medication

If laboratory results indicate (1) an ALT elevation > 2 × baseline and > 10 × ULN OR (2) abnormal laboratory parameters suggestive of worsening hepatic function (bilirubin ≥ 2 mg/dL above baseline, abnormal PT ≥ 2 secs above baseline, abnormal albumin ≥ 1 g/dL below baseline or elevated lactate levels > 2 × ULN) along with any ALT elevation (i.e., 1 grade shift or 2 × previous value) and the subject is on no post-study therapy for HBV, the following is recommended:

- Schedule the subject to return to the clinic as soon as possible and ideally no later than 1 week after the initial labs were drawn. During the visit, perform a clinical assessment of the subject. The assessment should include a physical examination and evaluation of the subject's mental status.
- Draw blood samples and request lactate testing and confirmation of elevated serum transaminases (ALT/AST), bilirubin, PT/INR, and albumin. [Note: If, in the investigator's judgment, the central lab cannot provide adequate turn around time, the confirmation test may also be performed at a local lab. However, the central lab results are considered definitive].
- If the elevations are confirmed (e.g., ALT > 2 × baseline and > 10 × ULN) OR (2) abnormal laboratory parameters suggestive of worsening hepatic function (abnormal bilirubin ≥ 2 mg/dL above baseline, abnormal PT ≥ 2 secs above baseline, abnormal albumin ≥ 1 g/dL below baseline, or elevated lactate levels > 2 × ULN) along with any ALT elevation (i.e., 1 grade shift or 2 × previous value), request the clinical laboratory to conduct reflex testing for serum HBV DNA, HBV serology (HBeAg, HBeAb, and HBsAg), HDV, HAV IgM and HCV. If serum HBV DNA is increasing, the investigator should consider immediate initiation of approved therapy.

- The subject should be followed until the abnormal ALT/AST values, bilirubin, PT/INR, albumin or lactate laboratory parameters return to normal or baseline up to a maximum of 6 months after the initial occurrence of the event. Refer to Appendix 8 for specific guidelines for the management of symptomatic and asymptomatic hyperlactatemia.

7.7. Risks for Women of Childbearing Potential or During Pregnancy

The risks of treatment with tenofovir DF during pregnancy have not been evaluated in pregnant women. Reproduction studies performed in rats and rabbits at doses up to 14–19 times the human dose based on body surface area comparisons revealed no evidence of impaired fertility or harm to the fetus due to tenofovir DF. There are, however, no adequate and well-controlled studies in pregnant women. Because animal reproduction studies are not always predictive of human response, tenofovir DF should be used during pregnancy only if clearly needed. Please refer to the latest version of the Investigator's Brochure for additional information.

Female subjects of childbearing potential must use barrier contraception in combination with other methods of contraception (e.g., oral or other hormonal contraceptives) or agree to abstain from heterosexual intercourse while participating in the study and for 30 days following the last dose of study medication. (A female subject of non-childbearing potential is menopausal ≥ 2 years, or has had a hysterectomy, bilateral oophorectomy, or medically-documented ovarian failure.) Male subjects in the study who are sexually active must use barrier contraception for the same period of time or agree to abstain from heterosexual intercourse. Females of childbearing potential must have a negative serum pregnancy test at screening. Lactating females must discontinue nursing prior to study drug administration.

Use of condoms should be encouraged for all participants, with or without other methods of contraceptives, because they have been proven to decrease the risk of contracting sexually transmitted diseases, including HBV, HCV, and HIV.

The subject must be instructed to discontinue all study drugs and inform the investigator **immediately** if she becomes pregnant during the study.

The investigator should report all pregnancies to the Medical Monitor within 24 hours of becoming aware of the pregnancy. The investigator should counsel the subject regarding the possible effects of prior study drug exposure on the fetus and the need to inform the study site of the outcome of the pregnancy.

The pregnancy itself is not considered an AE nor is an induced elective abortion to terminate a pregnancy without medical reasons.

However, an induced therapeutic abortion to terminate any pregnancy due to complications or other medical reasons will be recorded as an AE or an SAE. The underlying medical reason for this procedure should be recorded as the adverse event term.

A spontaneous abortion is always considered to be an SAE and will be reported as described in the Adverse and Serious Adverse Events section. Furthermore, any SAE occurring as an adverse pregnancy outcome post-study must be reported to the CRO by fax or email.

All pregnancies of female study subjects that occur during the study should be reported using the Pregnancy Report CRF page and the Gilead DSPH Pregnancy form and Pregnancy Outcome form.

Monitoring of the pregnancy in female study subjects should continue until the conclusion of the pregnancy. The outcome should be reported to Gilead DSPH using the Pregnancy Outcome form. If the end of the pregnancy occurs after the study has been completed, the outcome should be reported directly to Gilead DSPH.

8. STATISTICAL CONSIDERATIONS

8.1. Analysis Objectives

The primary objective of this study is:

- To evaluate the antiviral efficacy of tenofovir DF 300 mg once daily in Asian-American adults with chronic hepatitis B (CHB) infection

Secondary objectives are:

- To evaluate the safety and tolerability of tenofovir DF in Asian-American adults with CHB infection
- To evaluate the biochemical and serological responses to tenofovir DF in Asian-American adults with CHB infection
- To evaluate incidence of drug resistance mutations
- To evaluate change from baseline to Week 48 in FibroTest, a non-invasive biomarker of liver fibrosis

8.2. Primary Endpoint

The primary efficacy endpoint is HBV DNA < 400 copies/mL at Week 48.

8.3. Secondary Endpoints

For Week 48, secondary endpoints include:

- Safety/tolerability;
- ALT normal;
- Composite endpoint of HBV DNA < 400 copies/mL and ALT normal;
- Change from baseline in FibroTest value;
- HBeAg/HBsAg loss and seroconversion;
- HBV DNA < 169 copies/mL;
- Composite endpoint of HBV DNA < 400 copies/mL, ALT normal and HBeAg loss;
- Composite endpoint of HBV DNA < 400 copies/mL, ALT normal and HBe seroconversion;
- HBsAg loss and seroconversion;
- Development of drug resistance mutations.

8.4. Methods of Analysis

8.4.1. Analysis Sets

8.4.1.1. Efficacy

The primary analysis set for efficacy analysis will be defined as all subjects who received at least one dose of study drug. Subjects who withdraw after enrollment prior to receiving study drug will be excluded from the efficacy analysis set, as no data will be collected for these subjects. Subjects discontinuing therapy prior to Week 48 for non-administrative reasons will be considered failures at (and at all time after) the time of discontinuation of therapy for purposes of the Week 48 analysis. Subjects discontinuing therapy due to administrative reasons prior to Week 48 with plasma HBV DNA <400 copies/mL and no ongoing adverse event will be censored at the time of discontinuation.

8.4.1.2. Safety

The primary analysis set for safety analyses will include all subjects who received at least one dose of study drug. All data collected during the course of the study (on treatment and during treatment-free follow up) will be included in the safety summaries.

8.4.1.3. Pharmacokinetics

The PK analysis set will include all subjects who have evaluable pharmacokinetic data.

8.4.2. Data Handling Conventions

Missing data can have an impact upon the interpretation of the trial data. In general, values for missing data will not be imputed.

Data from non-completers (for non-administrative reasons) will be considered “failures” for the primary analysis of virological suppression at Week 48. Subjects discontinuing therapy due to administrative reasons prior to Week 48 with plasma HBV DNA < 400 copies/mL and no ongoing adverse event will be censored at the time of discontinuation.

Similarly, for the analysis of other secondary categorical efficacy endpoints, a subject will be considered a failure for these endpoints if the subject discontinues the trial for a non-administrative reason and has missed the visit at the time point of interest (non-completer equals failure approach). Sensitivity analyses will be performed if warranted. Subjects discontinuing therapy due to administrative reasons prior to Week 48 with plasma HBV DNA < 400 copies/mL and no ongoing adverse event will be censored at the time of discontinuation.

8.5. Demographic Data and Baseline Characteristics

Demographic and baseline measurements will be summarized using standard descriptive methods.

Demographic summaries will include gender and ethnic origin (e.g., Asian-Chinese, Asian-Indian, Asian-Korean, Asian-Vietamese, Asian-Japanese, Asian-Filipino, Asian-Cambodian, Asian-Pakistani, Asian-Laotian, Asian-Thai, Asian-Hmong, or Asian-Other).

Baseline data will include a summary of body weight, height, body mass index, $\log_{10}$ (HBV DNA) level, HBV serology, ALT/AST values, previous interferon exposure, and viral genotype.

8.6. Efficacy Analysis

8.6.1. Primary Analysis

The primary efficacy analysis will be conducted after the last subject reaches Week 48. The analysis will evaluate the proportion of subjects achieving the primary endpoint. Subjects discontinuing therapy due to administrative reasons prior to Week 48 with plasma HBV DNA < 400 copies/mL and no ongoing adverse event will be censored at the time of discontinuation.

8.6.2. Secondary Analyses

Secondary categorical endpoints will be summarized by number and percentage of subjects that meet the endpoint. For analyses of secondary efficacy endpoints, a subject will be considered a failure for these endpoints if the subject discontinues the trial for a non-administrative reason and has missed the visit at the time point of interest (non-completer equals failure approach). Subjects discontinuing therapy due to administrative reasons prior to Week 48 with plasma HBV DNA < 400 copies/mL and no ongoing adverse event will be censored at the time of discontinuation.

Week 48 FibroTest results will be analyzed as a non-invasive marker of hepatic fibrosis. The FibroTest results (continuous variable from 0 to 1.0) will be summarized using an 8-number summary (n, mean, standard deviation, median, Q1, Q3, min and max) at the Baseline and Week 48 Visits. In addition, the change from Baseline through Week 48 FibroTest value will be examined.

Subgroup analyses of efficacy endpoints may include analyses by the presence/absence of prior anti-HBV therapy.

8.7. Safety Analysis

All safety data collected on or after the date that study drug was first dispensed will be summarized. Data for the pretreatment and treatment-free follow-up periods will be included in listings.

The proportion of subjects with an AE leading to permanent discontinuation of study drug through Week 48 will be summarized.

8.7.1. Extent of Exposure

A subject's extent of exposure to study drug data will be generated from the study drug administration page of the CRF. Exposure data will be summarized.

8.7.2. Adverse Events

Clinical and laboratory adverse events will be coded using the Medical Dictionary for Regulatory Activities (MedDRA). System Organ Class (SOC), High Level Group Term (HLGT), High Level Term (HLT), Preferred Term, and Lower Level Term (LLT) will be attached to the clinical database. Severity of adverse events will be graded using the grading scale defined in Appendix 6.

Summaries (number and percentage of subjects) of treatment-emergent adverse events (by SOC, HLT and Preferred Term) will be provided as follows:

- all treatment-emergent adverse events,
- all related treatment-emergent adverse events,
- combined Grade 2, 3, and 4 treatment-emergent adverse events,
- combined Grade 3 and 4 treatment-emergent adverse events,
- combined Grade 2, 3, and 4 related treatment-emergent adverse events,
- combined Grade 3 and 4 related treatment-emergent adverse events,
- all adverse events that caused permanent discontinuation from study drug,
- all adverse events that caused permanent discontinuation from study,
- all adverse events that caused change in dose or temporary interruption of study drug,
- all serious adverse events, and
- all serious related adverse events.

Events will be summarized based on the date of onset for the event. A treatment-emergent adverse event will be defined as any adverse event that begins on or after the date of first dose of study drug. Events that occur prior to the first dose of study drug or after the last dose of study drug will be included in data listings.

8.7.3. Laboratory Evaluations

Selected laboratory data (using conventional units) will be summarized by the observed data and by the change from baseline across time.

Graded laboratory abnormalities will be defined using the grading scheme defined in Appendix 6. Grading of laboratory abnormalities for analysis purposes will be performed by the central laboratory.

Incidence of treatment-emergent laboratory abnormalities, defined as values that increase at least one toxicity grade from baseline at any time post baseline, will be summarized. If baseline data are missing, then any graded abnormality (i.e., at least a Grade 1) will be considered treatment-emergent.

Laboratory abnormalities that occur before the first dose of study drug or after the last dose of study drug will be included in a data listing.

8.8. Pharmacokinetic Analysis

Stored plasma samples from all subjects collected during each study visit may be utilized for tenofovir population pharmacokinetic analysis.

Plasma concentrations of tenofovir over time will be included in data listings.

8.9. Sample Size

Based on previous studies, a response rate of 84% is assumed for subjects receiving tenofovir DF.

The 95% confidence interval for this response rate with a sample size of 90 indicates that between 68 to 83 subjects are expected to achieve virologic response at Week 48.

9. RESPONSIBILITIES

9.1. Investigator Responsibilities

9.1.1. Good Clinical Practice

The investigator will ensure that this study is conducted in accordance with the principles of the "Declaration of Helsinki" (as amended in Edinburgh, Tokyo, Venice, Hong Kong, and South Africa), International Conference on Harmonisation (ICH) guidelines, or with the laws and regulations of the country in which the research is conducted, whichever affords the greater protection to the study subject. For studies conducted under a United States Investigational New Drug Application (IND), the investigator will ensure that the basic principles of "Good Clinical Practice," as outlined in 21 CFR 312, subpart D, "Responsibilities of Sponsors and Investigators," 21 CFR, part 50, 1998, and 21 CFR, part 56, 1998, are adhered to.

Since this is a "covered" clinical trial, the investigator will ensure that 21 CFR, Part 54, 1998, is adhered to; a "covered" clinical trial is any "study of a drug or device in humans submitted in a marketing application or reclassification petition subject to this part that the applicant or FDA relies on to establish that the product is effective (including studies that show equivalence to an effective product) or that make a significant contribution to the demonstration of safety." This requires that investigators and all sub-investigators must provide documentation of their financial interest or arrangements with Gilead, or proprietary interests in the drug being studied. This documentation must be provided before participation of the investigator and any subinvestigator. The investigator and sub-investigator agree to notify Gilead of any change reportable interests during the study and for one year following completion of the study. Study completion is defined as the date that the last subject has completed the protocol defined activities.

This study is also subject to and will be conducted in accordance with 21 CFR, part 320, 1993, "Retention of Bioavailability and Bioequivalence Testing Samples."

9.1.2. Institutional Review Board/Independent Ethics Committee Approval

This protocol and any accompanying material to be provided to the subject (such as advertisements, subject information sheets, or descriptions of the study used to obtain informed consent) will be submitted by the investigator to an IRB (for studies conducted in the United States) or IEC (for studies conducted outside of the United States). Approval from the IRB or IEC must be obtained **before** starting the study and should be documented in a letter to the investigator specifying the protocol number, protocol version, protocol date, documents reviewed, and date on which the committee met and granted the approval.

Any modifications made to the protocol after receipt of IRB or IEC approval must also be submitted to the IRB or IEC for approval before implementation.

9.1.3. Informed Consent

The investigator is responsible for obtaining written informed consent from each individual participating in this study after adequate explanation of the aims, methods, objectives, and potential hazards of the study and before undertaking any study-related procedures. The investigator must utilize an IRB- or IEC-approved consent form for documenting written informed consent. Each informed consent will be appropriately signed and dated by the subject or the subject's legally authorized representative and the person obtaining consent.

9.1.4. Confidentiality

The investigator must assure that subjects' anonymity will be strictly maintained and that their identities are protected from unauthorized parties. Only subject initials, date of birth and an identification code (i.e., not names) should be recorded on any form or biological sample submitted to the sponsor, IRB or IEC, or laboratory. The investigator must keep a screening log showing codes, names, and addresses for all subjects screened and for all subjects enrolled in the trial.

The investigator agrees that all information received from Gilead, including but not limited to the Investigator's Brochure, this protocol, CRFs, the investigational new drug, and any other study information, remain the sole and exclusive property of Gilead during the conduct of the study and thereafter. This information is not to be disclosed to any third party (except employees or agents directly involved in the conduct of the study or as required by law) without prior written consent from Gilead. The investigator further agrees to take all reasonable precautions to prevent the disclosure by any employee or agent of the study site to any third party or otherwise into the public domain.

9.1.5. Study Files and Retention of Records

The investigator must maintain adequate and accurate records to enable the conduct of the study to be fully documented and the study data to be subsequently verified. These documents should be classified into at least the following two categories: (1) investigator's study file, and (2) subject clinical source documents.

The investigator's study file will contain the protocol/amendments, CRF and query forms, IRB or IEC and governmental approval with correspondence, informed consent, drug records, staff curriculum vitae and authorization forms, and other appropriate documents and correspondence.

Subject clinical source documents (usually defined by the project in advance to record key activity/safety parameters independent of the CRFs) would include (although not be limited to) the following: subject hospital/clinic records, physician's and nurse's notes, appointment book, original laboratory reports, ECG, electroencephalogram, X-ray, pathology and special assessment reports, consultant letters, screening and enrollment log, etc.

All clinical study documents must be retained by the investigator until at least 2 years after the last approval of a marketing application in an ICH region (i.e., United States, Europe, or Japan) and until there are no pending or contemplated marketing applications in an ICH region; or, if no application is filed or if the application is not approved for such indication, until 2 years after the investigation is discontinued and regulatory authorities have been notified. Investigators may be required to retain documents longer if required by applicable regulatory requirements or an agreement with Gilead. The investigator must notify Gilead before destroying any clinical study records.

Should the investigator wish to assign the study records to another party or move them to another location, Gilead must be notified in advance.

If the investigator cannot guarantee this archiving requirement at the study site for any or all of the documents, special arrangements must be made between the investigator and Gilead to store these in sealed containers outside of the site so that they can be returned sealed to the investigator in case of a regulatory audit. When source documents are required for the continued care of the subject, appropriate copies should be made for storage outside of the site.

Biological samples at the conclusion of this study may be retained in storage by the Sponsor for a period up to 15 years for purposes of this study.

9.1.6. Case Report Forms

For each subject enrolled, a CRF must be completed and signed by the principal investigator or subinvestigator within a reasonable time period after data collection. This also applies to records for those subjects who fail to complete the study (even during a prerandomization screening period if a CRF was initiated). If a subject withdraws from the study, the reason must be noted on the CRF. If a subject is withdrawn from the study because of a treatment-limiting adverse event, thorough efforts should be made to clearly document the outcome.

9.1.7. Drug Accountability

The investigator or designee (i.e., pharmacist) is responsible for ensuring adequate accountability of all used and unused study drug. This includes acknowledgment of receipt of each shipment of study product (quantity and condition) and subject dispensing records and returned or destroyed study product. Dispensing records will document quantities received from Gilead and quantities dispensed to subjects, including lot number, date dispensed, subject identifier number, subject initials, and the initials of the person dispensing the medication.

At study initiation, the monitor will evaluate the site's standard operating procedure for study drug disposal/destruction in order to ensure that it complies with Gilead requirements. Drug may be returned or destroyed on an ongoing basis during the study if appropriate. At the end of the study, following final drug inventory reconciliation by the monitor, the study site will

dispose of and/or destroy all unused study drug supplies, including empty containers, according these procedures. If the site cannot meet Gilead's requirements for disposal, arrangements will be made between the site and Gilead or its representative for destruction or return of unused study drug supplies.

All drug supplies and associated documentation will be periodically reviewed and verified by the study monitor over the course of the study.

9.1.8. Inspections

The investigator should understand that source documents for this trial should be made available to appropriately qualified personnel from Gilead or its representatives, to IRBs or IECs, or to regulatory authority or health authority inspectors.

9.1.9. Protocol Compliance

The investigator is responsible for ensuring the study is conducted in accordance with the procedures and evaluations described in this protocol.

9.2. Sponsor Responsibilities

9.2.1. Protocol Modifications

Protocol modifications, except those intended to reduce immediate risk to study subjects, may be made only by Gilead. All protocol modifications must be submitted to the IRB or IEC in accordance with local requirements. Approval must be obtained before changes can be implemented.

9.2.2. Study Report and Publications

A clinical study report will be prepared and provided to the regulatory agency(ies). Gilead will ensure that the report meets the standards set out in the ICH Guideline for Structure and Content of Clinical Study Reports (ICH E3). Note that an abbreviated report may be prepared in certain cases.

After conclusion of the study and without prior written approval from Gilead, investigators in this study may communicate, orally present, or publish in scientific journals or other scholarly media ***only after the following conditions have been met:***

- the results of the study in their entirety have been publicly disclosed by or with the consent of Gilead in an abstract, manuscript, or presentation form; or
- the study has been completed at all study sites for at least 2 years.

No such communication, presentation, or publication will include Gilead's confidential information (see Section 9.1.4).

The investigator will submit any proposed publication or presentation along with the respective scientific journal or presentation forum at least 30 days before submission of the publication or presentation. The investigator will comply with Gilead's request to delete references to its confidential information (other than the study results) in any paper or presentation and agrees to withhold publication or presentation for an additional 60 days in order to obtain patent protection if deemed necessary.

9.3. Joint Investigator/Sponsor Responsibilities

9.3.1. Access to Information for Monitoring

In accordance with ICH Good Clinical Practice (ICH GCP) guidelines, the study monitor must have direct access to the investigator's source documentation in order to verify the data recorded in the CRFs for consistency.

The monitor is responsible for routine review of the CRFs at regular intervals throughout the study to verify adherence to the protocol and the completeness, consistency, and accuracy of the data being entered on them. The monitor should have access to any subject records needed to verify the entries on the CRFs. The investigator agrees to cooperate with the monitor to ensure that any problems detected in the course of these monitoring visits are resolved.

9.3.2. Access to Information for Auditing or Inspections

Representatives of regulatory authorities or of Gilead may conduct inspections or audits of the clinical study. If the investigator is notified of an inspection by a regulatory authority the investigator agrees to notify the Gilead Medical Monitor immediately. The investigator agrees to provide to representatives of a regulatory agency or Gilead access to records, facilities, and personnel for the effective conduct of any inspection or audit.

9.3.3. Study Discontinuation

Both the sponsor and the investigator reserve the right to terminate the study at any time. Should this be necessary, both parties will arrange discontinuation procedures and notify the appropriate regulatory authority(ies), IRBs, and IECs. In terminating the study, Gilead and the investigator will assure that adequate consideration is given to the protection of the subjects' interests.

10. REFERENCES

10 Heijtink RA, Kruining J, de Wilde GA, Balzarini J, De Clercq E, Schalm SW. Inhibitory effects of acyclic nucleoside phosphonates on human hepatitis B virus and duck hepatitis B virus infections in tissue culture. Antimicrob Agents Chemother 1994;38 (9):2180-2.

1131 Cherrington JM, Allen SJW, Bischofberger N, Chen MS. Kinetic interaction of the diphosphates of 9-(2-phosphonylmethoxyethyl)adenine and other anti-HIV active purine congeners with HIV reverse transcriptase and human DNA polymerases α, β, and γ. Antivir Chem Chemother 1995;6 (4):217-21.

1574 Robbins BL, Srinivas RV, Kim C, Bischofberger N, Fridland A. Anti-human immunodeficiency virus activity and cellular metabolism of a potential prodrug of the acyclic nucleoside phosphonate 9-*R*-(2-phosphonomethoxypropyl)adenine (PMPA), Bis(isopropyloxymethylcarbonyl) PMPA. Antimicrob Agents Chemother 1998;42 (3):612-7.

2331 World Health Organization Fact Sheet WHO/204 October 2000.

2516 Kramata P, Birkus G, Otmar M, Votruba I, Holy A. Structural features of acyclic nucleotide analogs conferring inhibitory effects on cellular replicative DNA polymerases. Collection Symposium Series (Holy A and Tocik Z, eds), Institute of Organic Chemistry and Biochemistry, Academy of Sciences of Czech Republic, Prague, Czech Republic 1996;1:188-91.

2820 Merican I, Guan R, Amarapuka D, Alexander MJ, Chutaputti A, Chien RN, et al. Chronic hepatitis B virus infection in Asian countries. J Gastroenterol Hepatol 2000;15 (12):1356-61.

3092 Poterucha JJ, Wiesner RH. Liver transplantation and hepatitis B. Ann Intern Med 1997;126 (8):805-7.

3273 Zuckerman AJ. Progress towards the comprehensive control of hepatitis B. Gut 1996;38 (2):S1.

3940 Nunez M, Perez-Olmeda M, Diaz B, Rios P, Gonzalez-Lahoz J, Soriano V. Activity of tenofovir on hepatitis B virus replication in HIV-co-infected patients failing or partially responding to lamivudine. AIDS 2002;16 (17):2352-4.

4121 Ristig MB, Crippin J, Aberg JA, Powderly WG, Lisker-Melman M, Kessels L, et al. Tenofovir disoproxil fumarate therapy for chronic hepatitis B in human immunodeficiency virus/hepatitis B virus-coinfected individuals for whom interferon-alpha and lamivudine therapy have failed. J Infect Dis 2002;186 (12):1844-7.

4199 Nelson M, Portsmouth S, Stebbing J, Atkins M, Barr A, Matthews G, et al. An open-label study of tenofovir in HIV-1 and Hepatitis B virus co-infected individuals. AIDS 2003;17 (1):F7-F10.

4376 Bruno R, Sacchi P, Zocchetti C, Ciappina V, Puoti M, Filice G. Rapid hepatitis B virus-DNA decay in co-infected HIV-hepatitis B virus 'e-minus' patients with YMDD mutations after 4 weeks of tenofovir therapy. AIDS 2003;17 (5):783-4.

4630 Benhamou Y, Tubiana R, Thibault V. Tenofovir disoproxil fumarate in patients with HIV and lamivudine-resistant hepatitis B virus. N Engl J Med 2003;348 (2):177-8.

6122 Piketty C, Benhamou Y. Efficacy of tenofovir disoproxil fumarate (TDF) in hepatitis B virus in HIV-coinfected patients: The TECOVIR study [abstract]. 11th Conference on Retroviruses and Opportunistic Infections; 2004 February 8-11; San Francisco, Calif, USA. Abstract P-15.

6556 Kuo A, Dienstag JL, Chung RT. Tenofovir disoproxil fumarate for the treatment of lamivudine-resistant hepatitis B. Clin Gastroenterol Hepatol 2004;2 (3):266-72.

6559 Kulig CC, Trotter JF, Everson GT. Treatment of lamivudine-resistant chronic hepatitis B with tenofovir [abstract]. Gastroenterology 2003;124 (Suppl 1):A-763. Abstract M2029.

6586 Dore GJ, Cooper DA, Pozniak AL, DeJesus E, Zhong L, Miller MD, et al. Efficacy of tenofovir disoproxil fumarate in antiretroviral therapy-naive and -experienced patients coinfected with HIV-1 and hepatitis B virus. J Infect Dis 2004;189 (7):1185-92.

6842 Nery J, Lau D, Nery C, Safdar K, Torres M, Neff GW, et al. Tenofovir and HBV Mutants After Liver Transplantation [AASLD abstract 342]. Gastroenterology 2003;124 (Suppl 1):A-710.

6843 Dore G, Cooper D, Pozniak A, Sayre J, Lu B, Miller M, et al. Anti-hepatitis B virus (HBV) activity in HBV/HIV co-infected patients treated with tenofovir DF (TDF) and lamivudine (LAM) versus LAM alone: 144-week follow-up [poster 3308]. XV International AIDS Conference; 2004 July 11-16; Bangkok, Thailand.

6849 Park J, Braun J, Kreiswirth S, Goldman D, Mullen M, Dieterich D. Co-infection with HIV and HBV: The Effect of Tenofovir Disoproxil Fumarate in Lamivudine and Famciclovir Experienced Patients [AASLD Abstract No. 1911]. Hepatology 2002;36 (4, Pt. 2):641A.

6850 Cecil BD. Tenofovir 300 mg Daily is Effective in Patients with Chronic HBV who Fail Lamivudine [AASLD Abstract No. 1868]. Hepatology 2002:630A.

6860 Marcelin AG, Tubiana R, Benhamou Y, Katlama C, Calvez V, Thibault V. Long-term Tenofovir Treatment of Lamivudine-resistant Chronic Hepatitis B in HIV Co-infected Patients [abstract 824]. 10th Conference on Retroviruses and Opportunistic Infections; 2003; Paris, France.

6868 Delaney WE, IV, Yang H, Sabogal A, Qi X, Miller M, Xiong S. Generation of novel stable cell lines for cross-resistance testing of anti-HBV compounds by real-time PCR in a 96 well format [oral presentation]. 17th International Conference on Antiviral Research; 2004 May 2-6; Tuscon, AZ, USA.

6870 Delaney WE, IV, Yang H, Miller MD, Gibbs CS, Xiong S. Combinations of adefovir with nucleoside analogs produce additive antiviral effects against hepatitis B virus in vitro. Antimicrob Agents Chemother 2004;48 (10):3702-10.

7060 Lada O, Benhamou Y, Cahour A, Katlama C, Poynard T, Thibault V. In vitro susceptibility of lamivudine-resistant hepatitis B virus to adefovir and tenofovir. Antivir Ther 2004;9 (3):353-63.

7176 Woolley IJ, Veitch AJ, Harangozo CS, Moyle M, Korman TM. Lichenoid drug erruption to tenofovir in an HIV/hepatitis B virus co-infected patient. AIDS 2004;18 (13):1857-8.

7208 Gilleece Y, Stebbing J, Clarke A, Fisher M, Bower M, Atkins M, et al. Tenofovir in the treatment of HBV in HIV-1 co-infected individuals [poster presentation]. XV International AIDS Conference; 2004 July 11-16; Bangkok, Thailand.

7209 Gomez J, Lopez JC, Cosin J, Miralles P, Berenguer J, Padilla B, et al. Tenofovir disoproxil fumarate (TDF) as treatment of chronic hepatitis B in HBV/HIV co-infected patients [abstract]. Presented at the XV International AIDS Conference; 2004 July 11-16; Bangkok, Thailand. Abstract MoPeB3338.

7210 Gozlan J, Lacombe K, Boelle EY, Beyer YJ, Charrois A, Carpentier S, et al. Activity of tenofovir disoproxil fumarate (TDF) on hepatitis B virus (HBV) in HIV-HBV co-infected individuals [poster]. Presented at the XV International AIDS Conference; 2004 July 11-16; Bangkok, Thailand. Poster MoPeB3334.

7211 Montessori V, Harris M, Sherlock C, Guillemi S, Toulson A, Chan K, et al. Tenofovir DF therapy for chronic hepatitis in HIV positive patients [abstract]. Presented at the XV International AIDS Conference; 2004 July 11-16; Bangkok, Thailand. Abstract B12685.

7213 van Bömmel F, Wünsche T, Mauss S, Reinke P, Bergk A, Schürmann D, et al. Comparison of adefovir and tenofovir in the treatment of lamivudine-resistant hepatitis B virus (HBV) infection. Hepatology 2004;40 (6):1421-5.

7459 Lucas JL, Carriero DC, Juriel A, Jaffe D, Dieterich DT. Effect of switching to tenofovir with emtricitabine or lamivudine in patients with chronic hepatitis B failing to respond to an adefovir-containing regimen [abstract]. 55th Annual Meeting of the American Association for the Study of Liver Diseases; 2004 October 29-November 2; Boston, Mass, USA. Abstract 1154.

7468 Schildgen O, Schewe CK, Vogel M, Däumer M, Kaiser R, Weitner L, et al. Successful therapy of hepatitis B with tenofovir in HIV-infected patients failing previous adefovir and lamivudine treatment. AIDS 2004;18 (17):2325-7.

7735 Neff GW, O'Brien CB, Nery J, Shire N, Montalbano M, Ruiz P, et al. Outcomes in liver transplant recipients with hepatitis B virus: resistance and recurrence patterns from a large transplant center over the last decade. Liver Transpl 2004;10 (11):1372-8.

7781 van Bömmel F, Mauss S, Wünsche T, Schürmann D, Wiedenmann B, Bergk A, et al. Frequency of HBeAg Loss and Long-term Antiviral Suppression Under Nucleos(t)ide Treatment in HBeAg Positive Chronic Hepatitis B (Abstract 532). 40th Annual Meeting of the European Association for the Study of the Liver; 2005 April 13-17; Paris, France.

7788 Qi X, Zhu Y, Curtis M, Yang H, Currie G, Miller MD, et al. In vitro cross-resistance analysis of the HBV polymerase mutation A181V [poster]. 40th Annual Meeting of the European Association for the Study of the Liver; 2005 April 13-17; Paris, France. Poster Number P-1295.

7790 Mauss S, Lutz T, van Boemmel F, Berger F, Berg T, Stoehr A, et al. Is Tenofovir Plus Lamivudine Superior to Tenofovir Monotherapy in Patients with HBV/HIV Coinfection? [abstract 516]. 40th Annual Meeting of the European Association for the Study of the Liver (EASL); 2005 April 13-17; Paris, France.

7792 Lacombe K, Gozlan J, Boelle P-Y, Serfaty L, Zoulim F, Valleron A-J, et al. Long-term hepatitis B virus dynamics in HIV–hepatitis B virus-co-infected patients treated with tenofovir disoproxil fumarate. AIDS 2005;19 (9):907-15.

7822 Bani-Sadr F, Palmer P, Scieux C, Molina JM. Ninety-six-week efficacy of combination therapy with lamivudine and tenofovir in patients coinfected with HIV-1 and wild-type hepatitis B virus. Clin Infect Dis 2004;39 (7):1062-4.

7833 Norris S, Taylor C, Muiesan P, Portmann BC, Knisely AS, Bowles M, et al. Outcomes of liver transplantation in HIV-infected individuals: the impact of HCV and HBV infection. Liver Transpl 2004;10 (10):1271-8.

7877 Neff GW, Nery J, Lau DTY, O'Brien CB, Duncan R, Shire NJ, et al. Tenofovir therapy for lamivudine resistance following liver transplantation. Ann Pharmacother 2004;38 (12):1999-2004.

8016 Brunelle M-N, Jacquard A-C, Pichoud C, Durantel D, Carrouée-Durantel S, Villeneuve J-P, et al. Susceptibility to Antivirals of a Human HBV Strain With Mutations Conferring Resistance to Both Lamivudine and Adefovir. Hepatology 2005;41 (6):1391-8.

8176 Menne S, Cote PJ, Korba BE, Butler SD, George AL, Tochkov IA, et al. Antiviral effect of oral administration of tenofovir disoproxil fumarate in woodchucks with chronic woodchuck hepatitis virus infection. Antimicrob Agents Chemother 2005;49 (7):2720-8.

8380 Sheldon J, Camino N, Rodes B, Bartholomeusz A, Kuiper M, Tacke F, et al. Selection of hepatitis B virus polymerase mutations in HIV-coinfected patients treated with tenofovir. Antivir Ther 2005;10 (6):727-34.

8381 Yang H, Qi X, Sabogal A, Miller M, Xiong S, Delaney WE, IV. Cross-resistance testing of next-generation nucleoside and nucleotide analogues against lamivudine-resistant HBV. Antivir Ther 2005;10 (5):625-33.

8455 van der Eijk AA, Hansen BE, Niesters HGM, Janssen HLA, van de Ende M, Schalm SW, et al. Viral dynamics during tenofovir therapy in patients infected with lamivudine-resistant hepatitis B virus mutants. J Viral Hepat 2005;12 (4):364-72.

8477 Schildgen O, Wend UC, Schewe CK, Vogel M, Daeumer M, Kaiser R, et al. Successful therapy of chronic hepatitis B with tenofovir in HBV-monoinfected and in HIV-coinfected patients failing previous adefovir and lamivudine treatment [abstract 527]. J Hepatol 2005;42 (Supplement 2):191.

8683 Tenney DJ, Levine SM, Rose RE, Walsh AW, Weinheimer SP, Discotto L, et al. Clinical emergence of entecavir-resistant hepatitis B virus requires additional substitutions in virus already resistant to Lamivudine. Antimicrob Agents Chemother 2004;48 (9):3498-507.

8805 Benhamou Y, Fleury H, Trimoulet P, Pellegrin I, Urbinelli R, Katlama C, et al. Anti-hepatitis B virus efficacy of tenofovir disoproxil fumarate in HIV-infected patients. Hepatology 2006;43 (3):548-55.

8806 Nunez M, Diaz-Pollan B, Camino N, Gonzalez-Lahoz J, Soriano V. Virological Outcome of Chronic Hepatitis B Virus (HBV) Infection in HIV-Coinfected Patients Receiving Anti-HBV Active Antiretroviral Therapy [Abstract V-1185]. 45th Interscience Conference on Antimicrobial Agents and Chemotherapy; 2005 December 16-19; Washington, DC.

8807 Trimoulet P, Thibault V, Schneider V, Pellegrin I, Bocket L, Schmitt MP, et al. Anti-hepatitis B Virus (HBV) Activity of Tenofovir Disoproxil Fumarate (TDF) in Human Immunodeficiency Virus (HIV) Coinfected Patients [Abstract V-783]. 43rd Interscience Conference on Antimicrobial Agents and Chemotherapy; 2003 September 14 - 17, 2003; Chicago, IL.

8809 Stephan C, Berger A, Carlebach A, Lutz T, Bickel M, Klauke S, et al. Impact of tenofovir-containing antiretroviral therapy on chronic hepatitis B in a cohort co-infected with human immunodeficiency virus. J Antimicrob Chemother 2005;56 (6):1087-93.

8875 van Bömmel F, Mauss S, Zöllner B, Schürmann D, Bergk A, Feucht H-H, et al. Long-term effect of tenofovir in the treatment of lamivudine-resistant hepatitis B virus infections in comparison to adefovir [Supplement 1 - abstract 184]. Hepatology 2005;42 (4):269A.

8876 Mauss S, Nelson M, Lutz T, Sheldon J, Bruno R, van Boemmel F, et al. First Line Combination Therapy of Chronic Hepatitis B With Tenofovir Plus Lamivudine Versus Sequential Therapy with Tenofovir Monotherapy After Lamivudine Failure [Supplement 1 - abstract 964]. Hepatology 2005;42 (4):574A.

8877 Klausen G, Moll A, Gölz J, Schleehauf D, Prziwara D, Nzimegne-Gölz S. Efficacy of Tenofovir Against HIB in HIV/HBV Coinfected Patients [Supplement 1 - abstract 977]. Hepatology 2005;42 (4):580A.

8879 Villeneuve JP, Willems B, Zoulim F. Efficacy of Tenofovir in Patients with Chronic Hepatitis B and Resistance or Sub-Optimal Response to Adefovir [Supplement 1 - abstract 997]. Hepatology 2005;42 (4):588A.

8880 Im GY, Uriel AJ, Carriero D, Park J, Jaffe DL, Dieterich DT. Comparison of Tenofovir Versus Adefovir Based Combination Therapy in Subjects with Chronic Hepatitis B [Supplement 1 - abstract 999]. Hepatology 2005;42 (4):589A.

8881 van Bömmel F, Möller B, Spengler U, Zöllner B, Sarrazin C, Hüppe D, et al. Tenofovir Rescue for Patients with Lamivudine Resistant HBV Infection with Suboptimal Virologic Response to Adefovir [Supplement 1 - abstract 1000]. Hepatology 2005;42 (4):589A.

8893 Nelson M, Bhagani S, Fisher M, Leen C, Brook G, Mandalia S, et al. A 48-week Study of Tenofovir or Lamivudine or a Combination of Tenofovir and Lamivudine for the Treatment of Chronic Hepatitis B in HIV/HBV-co-infected Individuals [poster 831]. 13th Conference on Retroviruses and Opportunistic Infections; 2006 February 5 - 8; Denver, CO.

9241 van Bommel F, Zollner B, Sarrazin C, Spengler U, Huppe D, Moller B, et al. Tenofovir for patients with lamivudine-resistant hepatitis B virus (HBV) infection and high HBV DNA level during adefovir therapy. Hepatology 2006;44 (2):318-25.

9266 Delaney WE, IV, Ray AS, Yang H, Qi X, Xiong S, Zhu Y, et al. Intracellular metabolism and in vitro activity of tenofovir against hepatitis B virus. Antimicrob Agents Chemother 2006;50 (7):2471-7.

10408 Peters MG, Andersen J, Lynch P, Liu T, Alston-Smith B, Brosgart CL, et al. Randomized controlled study of tenofovir and adefovir in chronic hepatitis B virus and HIV infection: ACTG A5127. Hepatology 2006;44 (5):1110-6.

10425 Villet S, Pichoud C, Villeneuve JP, Trepo C, Zoulim F. Selection of a multiple drug-resistant hepatitis B virus strain in a liver-transplanted patient. Gastroenterology 2006;131 (4):1253-61.

10426 Lacombe K, Ollivet A, Gozlan J, Durantel S, Tran N, Girard PM, et al. A novel hepatitis B virus mutation with resistance to adefovir but not to tenofovir in an HIV-hepatitis B virus-co-infected patient. AIDS 2006;20 (17):2229-31.

10466 Colonno RJ, Rose R, Baldick CJ, Levine S, Pokornowski K, Yu CF, et al. Entecavir resistance is rare in nucleoside naive patients with hepatitis B. Hepatology 2006;44 (6):1656-65.

10612 Jain MK, Comanor L, White C, Kipnis P, Elkin C, Leung K, et al. Treatment of hepatitis B with lamivudine and tenofovir in HIV/HBV-coinfected patients: factors associated with response. J Viral Hepat 2007;14 (3):176-82.

10665 Lok AS, McMahon BJ. Chronic hepatitis B. Hepatology 2007;45 (2):507-39.

10684 Chen CJ, Yang HI, Su J, Jen CL, You SL, Lu SN, et al. Risk of hepatocellular carcinoma across a biological gradient of serum hepatitis B virus DNA level. JAMA 2006;295 (1):65-73.

10690 Disease Burden from Hepatitis A, B, and C in the United States. www.cdc.gov/ncidod/diseases/hepatitis/resource/PDFs/disease_burden.pdf. Accessed April 12, 2007.

10804 Tenney DJ, Rose RE, Baldick CJ, Levine SM, Pokornowski KA, Walsh AW, et al. Two-year assessment of entecavir resistance in Lamivudine-refractory hepatitis B virus patients reveals different clinical outcomes depending on the resistance substitutions present. Antimicrob Agents Chemother 2007;51 (3):902-11.

10916 Brunelle MN, Lucifora J, Neyts J, Villet S, Holy A, Trepo C, et al. In Vitro Activity of 2,4-Diamino-6-[2-(Phosphonomethoxy)Ethoxy]-Pyrimidine against Multidrug-Resistant Hepatitis B Virus Mutants. Antimicrob Agents Chemother 2007;51 (6):2240-3.

10926 Qi X, Xiong S, Yang H, Miller M, Delaney WE, IV. In vitro susceptibility of adefovir-associated hepatitis B virus polymerase mutations to other antiviral agents. Antivir Ther 2007;12 (3):355-62.

10946 Villet S, Ollivet A, Pichoud C, Barraud L, Villeneuve JP, Trepo C, et al. Stepwise process for the development of entecavir resistance in a chronic hepatitis B virus infected patient. J Hepatol 2007;46 (3):531-8.

10952 Wright TL. Introduction to chronic hepatitis B infection. Am J Gastroenterol 2006;101:6.

11362 Heathcote EJ, Gane E, DeMan R, Lee S, Flisiak R, Manns MP, et al. A Randomized, Double-Blind Comparison of Tenofovir DF (TDF) Versus Adefovir Dipivoxil (ADV) for the Treatment of HBEAG-Positive Chronic Hepatitis B (CHB): Study GS-US-174-0103 [AASLD Abstracts]. Hepatology 2007;46 (4 Supp1):861A.

11363 Marcellin P, Buti M, Kraslev Z, Germanidis G, Kaita K, Kotzev I, et al. A Randomized, Double-Blind Comparison of Tenofovir DF (TDF) Versus Adefovir Dipivoxil (ADV) for the Treatment of HBEAG-Negative Chronic Hepatitis B (CHB): Study GS-US-174-0102 [AASLD Abstracts]. Hepatology 2007;46 (4 Suppl 1):290A-1A.

11915 van Bommel F, de Man RA, Erhardt A, Huppe D, Stein K, Buggisch P, et al. First multicenter evaluation of the efficacy of Tenofovir in nucleos(t)ide analog experienced patients with HBV monoinfection [oral presentation]. 58th Annual Meeting of the American Association for the Study of Liver Diseases (AASLD); 2007 November 2-6; Boston, MA.

12036 Nguyen TT, Taylor V, Chen Jr, MS, Bastani R, Maxwell AE, McPhee SJ. Hepatitis B awareness, knowledge, and screening among Asian Americans. J Cancer Educ 2007; 22(4):266-72.

12037 Poynard T, Morra R, Halfon P, Castera L, Ratziu V, Imbert-Bismet, F, et al. Meta-analyses of FibroTest diagnostic value in chronic liver disease. BMC Gastroenterology 2007;7:40.

12038 Lin SY, Chang ET, So SK. Why we should routinely screen Asian American adults for hepatitis B: a cross-sectional study of Asians in California. Hepatology 2007;46:1034-1040.

12039 Hutton DW, Tan D, So SK, Brandeau ML. Cost-effectiveness of screening and vaccinating Asian and Pacific Islander adults for hepatitis B. Ann Intern Med 2007;147(7):460-9.

12043 Hongbo L, Xiaohui L, Hong K, Wei W, Yong Z. Assessing routine and serum markers of liver fibrosis in CHB patients using parallel and serial interpretation. Clin Biochem 2007;40 (8):562-6.

11. APPENDICES

Appendix 1. Investigator Signature Page
Appendix 2. Study Procedures Table
Appendix 3. Clinical Laboratory Assessments
Appendix 4. Blood Sample Collection and Processing for Determination of Tenofovir Concentrations in Plasma
Appendix 5. Management of Clinical and Laboratory Adverse Events
Appendix 6. GSI Grading Scale for Severity of Adverse Events and Laboratory Abnormalities
Appendix 7. Summary of Anti-Viral Activity of Tenofovir DF in Chronic Hepatitis B Patients* or Chronic Hepatitis B Patients Co-infected with HIV**
Appendix 8. Lactic Acidosis Guidelines

Appendix 1. Investigator Signature Page

GILEAD SCIENCES, INC.
4 UNIVERSITY PLACE
4611 UNIVERSITY DRIVE DURHAM, NC 27707
STUDY ACKNOWLEDGEMENT

A Phase IV Study to Evaluate the Efficacy, Safety and Tolerability of Tenofovir DF in Asian-American Adults with Chronic Hepatitis B Infection

(Administrative Amendment 1, August 26, 2008)

This protocol has been approved by Gilead Sciences, Inc. The following signature documents this approval.

S. Diane Goodwin, PharmD	S. Diane Goodwin
Author	Signature

26 August 2008
Date

INVESTIGATOR STATEMENT

I have read the protocol, including all appendices, and I agree that it contains all necessary details for me and my staff to conduct this study as described. I will conduct this study as outlined herein and will make a reasonable effort to complete the study within the time designated.

I will provide all study personnel under my supervision copies of the protocol and access to all information provided by Gilead Sciences, Inc. I will discuss this material with them to ensure that they are fully informed about the drugs and the study.

Principal Investigator Name (Printed)	Signature
Date	Site Number

Appendix 2. Study Procedures Table

Study Procedures	Screening[a]	Baseline	Study Week							Early DC[b]	24-Wk Follow Up[c]
			4	8	16	24	32	40	48		
Written Informed Consent	X										
Medical History, HBV History, Treatment History	X	X[d]									
Complete Physical Examination	X	X				X			X	X	
Symptom-Directed Physical Examination			X	X	X		X	X			X
Vitals Signs, Height, Weight[e]	X	X	X	X	X	X	X	X	X	X	X
HIV-1, HCV, HDV, α-fetoprotein	X										
Hepatic ultrasound or CT scan[f]	X										
HBV DNA Levels (PCR-Based Assay)	X	X	X	X	X	X	X	X	X	X	X
HBV Serology[g]	X	X			X		X		X	X	X[h]
HBV Genotyping, Resistance Surveillance[i]		X							X	X	
HBV Drug Resistance Test (INNOLiPA)		X									
Hematology Profile	X	X	X	X	X	X	X	X	X	X	
Serum Chemistry and Liver Tests[j]	X	X	X	X	X	X	X	X	X	X	X
Prothrombin Time/INR[k]	X	X									X[k]
Urinalysis	X	X	X	X	X	X	X	X	X	X	
Pregnancy Test[l]	X	X	X	X	X	X	X	X	X	X	
Plasma for Tenofovir Concentration Determination		X	X	X	X	X	X	X	X	X	
Serum and Plasma for Storage[m]	X	X	X	X	X	X	X	X	X	X	X
FibroTest analytes: α_2-macroglobulin, Apolipoprotein A_1, Gamma-glutamyl transferase, Haptoglobin (Total bilirubin in Serum Chemistry/Liver Test panel)		X							X	X	
Concomitant Medications	X	X	X	X	X	X	X	X	X	X	X
Adverse Events	X	X	X	X	X	X	X	X	X	X	X

Appendix 2. Study Procedures Table (Continued)

Study Procedures	Screening[a]	Baseline	Study Week 4	8	16	24	32	40	48	Early DC[b]	24-Wk Follow Up[c]
Study Drug Dispensing/Return[n]		X	X	X	X	X	X	X	X	X	
Drug Accountability/Adherence Assessment			X	X	X	X	X	X	X	X	
Identify Mechanism for Post-Study HBV Treatment							X				

a Evaluations to be completed within 6 weeks (42 days) prior to Baseline Visit.

b The Early Study Drug Discontinuation (DC) Visit should be completed within 72 hours of last dose of study drug; subjects who discontinue study drug will be followed for 24 weeks off treatment or up to initiation of active treatment, whichever comes first.

c Subjects who receive at least one dose of study drug will be followed for 24 weeks off treatment or up to initiation of active treatment, whichever occurs first.

d Review of medical history and any changes since Screening Visit, including changes in concomitant medications.

e Vital signs = temperature, blood pressure, heart rate, respiratory rate; height will be measured at the Screening and Baseline Visits only.

f Subjects > 40 years of age with α-fetoprotein <50 ng/mL must undergo hepatic ultrasound or CT scan to rule out hepatocellular carcinoma during screening.

g HBeAg and HBsAg and, if negative, reflex HbeAb and HBsAb, respectively.

h During the 24-week follow-up period, HBV serology testing will only be performed at the Follow-Up Week 24 Visit.

i Determination of HBV viral genotype (A–H) will be performed at the Baseline Visit for all subjects. Sequencing analysis of the HBV polymerase using di-deoxy sequencing will be conducted at Baseline for all subjects, and attempted for all viremic subjects (HBV DNA ≥ 400 c/mL) at Week 48, as well as at the Early Discontinuation Visit, if applicable.

j Including estimated creatinine clearance, by the Cockcroft-Gault Equation.

k PT/INR will be performed at the Screening and Baseline Visits, then as a reflex test in subjects experiencing exacerbation of hepatitis thereafter.

l A serum β-HCG pregnancy test will be performed at Screening, Baseline, and Week 48, as well as the Early Study Drug Discontinuation Visit (if applicable). Urine pregnancy testing will be performed at all other study visits through the end of the study. A positive urine pregnancy test must be confirmed immediately with a serum pregnancy test.

m Blood (serum and plasma) collected for longterm storage and possible future testing (e.g., bioanalytical and virologic analysis).

n Initiation of study drugs must occur within 24 hours after the Baseline Visit. At every study visit, subjects will return unused study medication, and new study medication will be dispensed. After each subject has reached Week 48 (or at the Early Study Drug Discontinuation Visit), study medication will not be dispensed. Subjects who have received at least one dose of study drug and permanently discontinue study drug will be followed for 24 weeks off treatment or up to initiation of active treatment, whichever occurs first.

Appendix 3. Clinical Laboratory Assessments

**Hematology:**	Erythrocytes<br>Hemoglobin<br>Hematocrit<br>Platelets<br>Total leukocytes and differential (neutrophils, lymphocytes, monocytes, eosinophils, basophils)	**Liver Tests:**	Total bilirubin (reflex direct [conjugated] bilirubin)<br>AST<br>ALT<br>Alkaline phosphatase<br>Prothrombin time, INR
**Chemistry:**	Creatine phosphokinase<br>Lactic dehydrogenase<br>Creatinine and creatinine clearance<br>Albumin<br>Glucose<br>Serum amylase<br>Lipase (if serum amylase is ≥ 1.5 × ULN)<br>α-fetoprotein (Screening Visit)<br>Blood urea nitrogen (BUN)<br>Uric acid	**Urinalysis:**<br><br><br><br><br>**Urine Pregnancy Test:**	Protein<br>Blood<br>Glucose<br><br><br>Females of child-bearing potential (reflex serum β-HCG if positive)
**Electrolytes:**	Sodium<br>Potassium<br>Bicarbonate<br>Phosphorus<br>Calcium<br>Magnesium<br>Chloride	**HBV Serology:**	HBsAg, HBeAg (reflex HBsAb and HBeAb).
**Serum β-HCG Pregnancy Test:**	Females of child-bearing potential; at Screening, Baseline, Week 48 or Early Study Discontinuation Visits; reflex if urine test positive at all other study visits	**Virology:**	Plasma and serum for HBV DNA and serology<br>HIV, HDV and HCV (Screening Visit)<br>Viral genotyping (A–H), resistance surveillance by di-deoxy sequencing<br>INNOLiPA assay at Baseline Visit
**Labs for FibroTest:**	α_2-macroglobulin, Apolipoprotein A_1, Gamma-glutamyl transferase, Haptoglobin (Total bilirubin is included in Liver Tests)		
**Tenofovir DF Concentrations**:	Plasma collected at study visits	**Storage:**	Plasma and serum for storage

Appendix 4. Blood Sample Collection and Processing for Determination of Tenofovir Concentrations in Plasma

For all subjects, at baseline and all subsequent study visits, including any Early Study Drug Discontinuation Visits, plasma will be stored for determination of tenofovir concentrations in plasma. At the time each blood sample is collected, the following information will be recorded:

- The date and time of last dose taken
- Whether last dose was taken with or without food
- The date and time of blood draw following last dose taken

Draw approximately 3 mL of whole blood at each study visit, according to schedule in Appendix 2 (blood sample will not be collected for determination of tenofovir DF plasma concentrations at the screening or 24-week follow-up visits). Collect the blood in a 3-mL spray-dried K_2-EDTA-containing blood collection tube.

Invert the blood collection tube immediately after drawing the specimen. Invert the tube 8–10 times to allow mixing of the blood and the K_2-EDTA anticoagulant.

Centrifuge each blood specimen within 30 minutes of blood collection to separate the plasma in a refrigerated centrifuge (approximately 4^oC). The exact centrifuge settings will be determined by the type and model of centrifuge. A selection of at least 1500 X g for a minimum of 15 minutes should be employed. The blood sample should be kept on wet ice or refrigerated during the interval between obtaining the blood sample and centrifugation.

Transfer the separated plasma from each EDTA tube and divide between two, appropriately labeled 4 mL (3.6 mL fill) round-bottom storage tubes with externally-threaded screw caps (one tube serving as duplicate) provided in the PK kit. The storage tubes in the PK kit are pre-labeled. The labels are pre-printed with the study number (GS-US-174-0123), the collection time point, and either “Plasma A” or “Plasma B”. The tubes labeled with “Plasma A” are primary storage tubes and must be sent to the central laboratory immediately with other laboratory specimens, following completion of blood draws for all time points. The tubes labeled with “Plasma B” are the back-up storage tubes and must be stored in the site’s freezer at least until the central laboratory confirms receipt of the primary tubes (“Plasma A”). Study sites without freezers (-20^oC or -70^oC) may ship both primary “Plasma A” and back-up samples “Plasma B” to Covance on the same day, as directed by the sponsor. Both sets of tubes will also include a line to write in the subject number and the subject initials. It is very important to include the correct subject number and subject initials on each label so that the storage tubes will be processed correctly at the central laboratory.

Store the storage tubes containing the plasma samples in an upright position in a non-self-defrosting freezer with the temperature set to maintain -20^oC or lower until shipment to the central laboratory. The storage tubes should be placed in the freezer within 2 hours of blood collection. The storage tubes must be sent to the central laboratory on dry ice in appropriate shipping boxes.

Record all back-up storage tubes (labeled with "Plasma B") on the Back-Up Specimen Freezer Log provided to the site. The log includes the date of collection and confirmation of placement in the freezer, as well as the ship date to the central laboratory and tracking number. Note: All sites should ship back-up specimens, frozen on dry ice and in batches if possible, to the central laboratory as soon as receipt of first specimen ("Plasma A") has been confirmed. Sites can confirm the central laboratory receipt by tracking the shipment with the designated courier.

Plasma samples will be sent from the central laboratory to Gilead Sciences, Durham, North Carolina, USA for analysis upon request.

It is the responsibility of the Investigator to ensure that all samples for interstate or international transport are appropriately handled (packaged and shipped). Packaging and shipping shall be performed by an individual(s) that has/have documented training for safety, general awareness and function specific areas associated with shipment and handling of hazardous materials.

The samples must be clearly labeled for sample identification, inventoried and organized in sample boxes. The sample boxes should be placed into certified performance-tested Styrofoam shipping boxes with sufficient dry ice to ensure they are frozen upon receipt (minimum 2 days). Only certified performance-tested Styrofoam shipping boxes that meet the criteria set forth in the appropriate IATA packaging instructions are to be employed for the transport of samples from studies conducted by Gilead Sciences, Inc. The samples must be packaged and labeled in accordance with the appropriate IATA and DOT guidelines. Labels indicating that the shipment contains dry ice (UN 1845) shall be affixed to the outside of the shipping container. The weight of dry ice employed (in kilograms) must also be listed. Phone numbers for the shipper and consignee shall appear on the appropriate shipping labels. The shipper shall provide a 24-hour contact number in the event of damage to the shipment.

As of 01/01/07, specimens known or suspected of containing pathogens meeting the criteria for risk groups 2 or 3 may be transported as "Biological Substance, Category B" when they are transported for diagnostic or investigational purposes. Packing instruction 650 is appropriate for the transport of specimens containing pathogens belonging to risk Group 2 and 3. A UN 3373 label must be affixed to the outside of the shipping container in the diamond on point orientation. The UN 3373 number is required to be shown. As of 01/01/07, shipments of specimens previously labeled as Diagnostic Specimens will be required to be labeled as "Biological Substance, Category B". The new labeling ("Biological Substance, Category B") must now be used in lieu of the phrase "Diagnostic Specimens. All other elements of IATA Packing Instruction 650 remain unchanged. Specimens known or suspected of containing risk group 4 pathogens must be classified in Division 6.2 under UN 2814 or UN 2900, as appropriate, and transported according to the requirements for these substances. Packages containing infectious substances must be labeled with the appropriate Class 6 Infectious Substance label.

Failure to comply with these regulations can result in imprisonment and/or fines.

Appendix 5. Management of Clinical and Laboratory Adverse Events

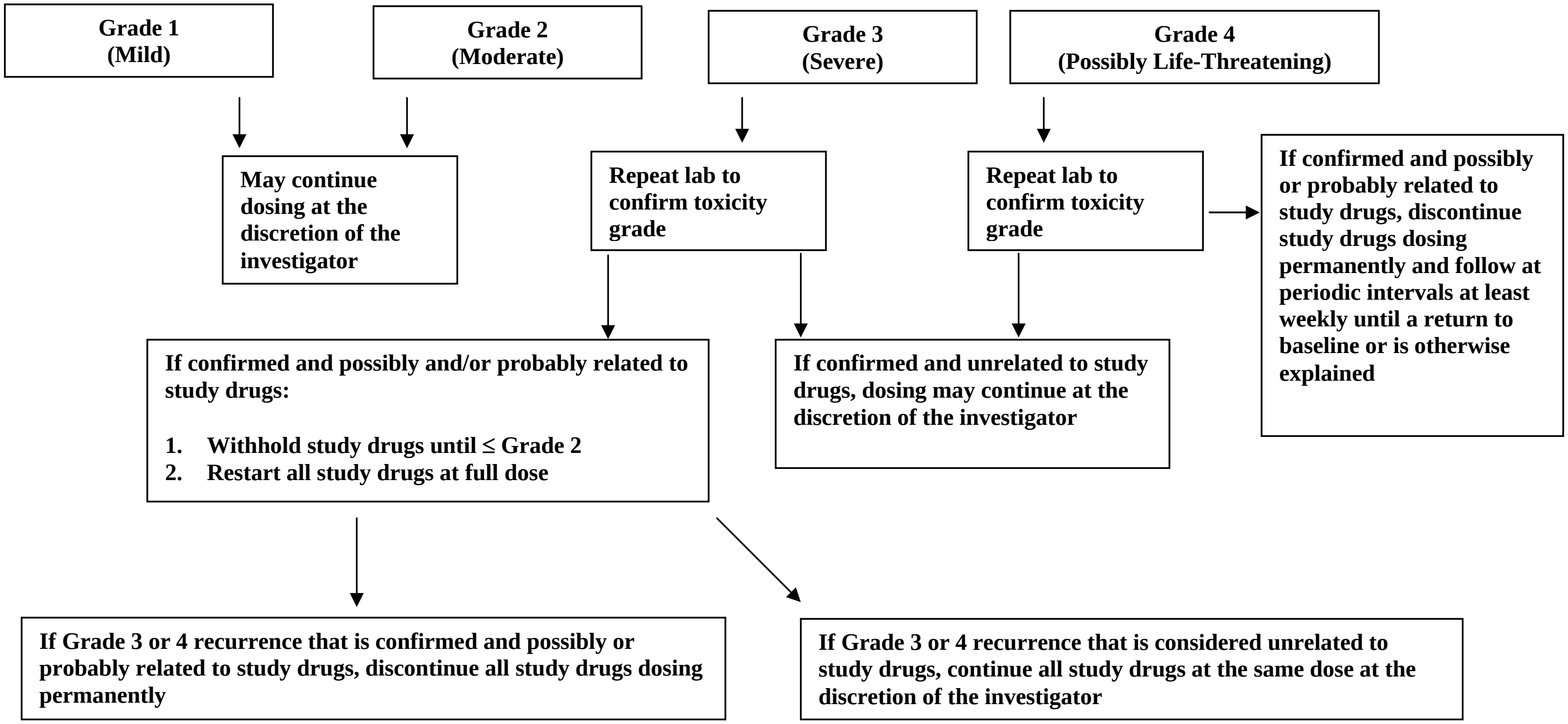

Appendix 6. GSI Grading Scale for Severity of Adverse Events and Laboratory Abnormalities

HEMATOLOGY

	Grade 1	Grade 2	Grade 3	Grade 4
Hemoglobin	8.0 – 9.4 g/dL<br>80 – 94 g/L	7.0 – < 8.0 g/dL<br>70 – < 80 g/L	6.5 – < 7.0 g/dL<br>65 – < 70 g/L	< 6.5 g/dL<br>< 65 g/L
Absolute Neutrophil Count (ANC)	1000 – 1500/mm^3<br>1.00 – 1.50 GI/L	750 – < 1000/mm^3<br>0.75 – < 1.00 GI/L	500 – < 750/mm^3<br>0.50 – < 0.75 GI/L	< 500/mm^3<br>< 0.50 GI/L
Platelets	75,000 – 100,000/mm^3<br>75.0 – 100.0 GI/L	50,000 – < 75,000/mm^3<br>50.0 – < 75.0 GI/L	25,000 – < 50,000/mm^3<br>25.0 – < 50.0 GI/L	< 25,000/mm^3<br>< 25.0 GI/L
WBCs	3000/mm^3 – < LLN<br>3.0 GI/L – < LLN	2000 – < 3000/mm^3<br>2.0 – < 3.0 GI/L	1000 – < 2000/mm^3<br>1.0 – < 2.0 GI/L	< 1000/ mm^3<br>< 1.0 GI/L
Hypofibrinogenemia	100 – 200 mg/dL<br>1.00 – 2.00 g/L	75 – < 100 mg/dL<br>0.75 – < 1.00 g/L	50 – < 75<br>0.50 – < 0.75 g/L	< 50 mg/dL<br>< 0.50 g/L
Hyperfibrinogenemia	400 – 600 mg/dL<br>4.0 – 6.0 g/L	> 600 mg/dL<br>> 6.0 g/L	—	—
Fibrin Split Product	20 – 40 microg/mL<br>20 – 40 mg/L	> 40 – 50 microg/mL<br>> 40 – 50 mg/L	> 50 – 60 microg/mL<br>> 50 – 60 mg/L	> 60 microg/mL<br>> 60 mg/L
Prothrombin Time (PT)	> 1.00 – 1.25 × ULN	> 1.25 – 1.50 × ULN	> 1.50 – 3.00 × ULN	> 3.00 × ULN
Activated Partial Thromboplastin (APPT)	> 1.00 – 1.66 × ULN	> 1.66 – 2.33 × ULN	> 2.33 – 3.00 × ULN	> 3.00 × ULN
Methemoglobin	5.0 – 10.0%	> 10.0 – 15.0%	> 15.0 – 20.0%	> 20.0%

Continued on following page

Appendix 6. GSI Grading Scale for Severity of Adverse Events and Laboratory Abnormalities (Continued)

HEMATOLOGY (Continued)

	Grade 1	Grade 2	Grade 3	Grade 4
Hyponatremia	130 mEq/L – < LLN<br>130 mmol/L – < LLN	123 – < 130 mEq/L<br>123 – < 130 mmol/L	116 – < 123 mEq/L<br>116 – < 123 mmol/L	< 116 mEq/L<br>< 116 mmol/L
Hypernatremia	> ULN – 150 mEq/L<br>> ULN – 150 mmol/L	> 150 – 157 mEq/L<br>> 150 – 157 mmol/L	> 157 – 165 mEq/L<br>> 157 – 165 mmol/L	> 165 mEq/L<br>> 165 mmol/L
Hypokalemia	3.0 mEq/L – < LLN<br>3.0 mmol/L – < LLN	2.5 – < 3.0 mEq/L<br>2.5 – < 3.0 mmol/L	2.0 – < 2.5 mEq/L<br>2.0 – < 2.5 mmol/L	< 2.0 mEq/L<br>< 2.0 mmol/L
Hyperkalemia	5.6 – 6.0 mEq/L<br>5.6 – 6.0 mmol/L	> 6.0 – 6.5 mEq/L<br>> 6.0 – 6.5 mmol/L	> 6.5 – 7.0 mEq/L<br>> 6.5 – 7.0 mmol/L	> 7.0 mEq/L<br>> 7.0 mmol/L
Hypoglycemia	55 – 64 mg/dL<br>3.03 – 3.57 mmol/L	40 – < 55 mg/dL<br>2.20 - < 3.03 mmol/L	30 – < 40 mg/dL<br>1.64 – < 2.20 mmol/L	< 30 mg/dL<br>< 1.64 mmol/L
Hyperglycemia (nonfasting and no prior diabetes)	> ULN – 160 mg/dL<br>> ULN – 8.90 mmol/L	> 160 - 250 mg/dL<br>> 8.90 – 13.90 mmol/L	> 250 – 500 mg/dL<br>> 13.90 – 27.77 mmol/L	> 500 mg/dL<br>> 27.77 mmol/L
Hypocalcemia<br>(corrected for albumin)	7.8 mg/dL – < LLN<br>1.94 mmol/L – < LLN	7.0 – < 7.8 mg/dL<br>1.74 – < 1.94 mmol/L	6.1 – < 7.0 mg/dL<br>1.52– < 1.74 mmol/L	< 6.1 mg/dL<br>< 1.52 mmol/L
Hypercalcemia<br>(corrected for albumin)	> ULN – 11.5 mg/dL<br>> ULN – 2.88 mmol/L	> 11.5 – 12.5 mg/dL<br>> 2.88 – 3.13 mmol/L	> 12.5 – 13.5 mg/dL<br>> 3.13 – 3.38 mmol/L	> 13.5 mg/dL<br>> 3.38 mmol/L
Hypocalcemia (ionized)	3.0 mg/dL – < LLN<br>0.74 mmol/L – < LLN	2.5 – < 3.0 mg/dL<br>0.62 - < 0.74 mmol/L	2.0 – < 2.5 mg/dL<br>0.49 - < 0.62 mmol/L	< 2.0 mg/dL<br>< 0.49 mmol/L

Continued on following page

Appendix 6. GSI Grading Scale for Severity of Adverse Events and Laboratory Abnormalities (Continued)

HEMATOLOGY (Continued)				
	**Grade 1**	**Grade 2**	**Grade 3**	**Grade 4**
Hypercalcemia (ionized)	> ULN – 6.0 mg/dL<br>> ULN – 1.51 mmol/L	> 6.0 – 6.5 mg/dL<br>> 1.51 – 1.63 mmol/L	> 6.5 – 7.0 mg/dL<br>> 1.63 – 1.76 mmol/L	> 7.0 mg/dL<br>> 1.76 mmol/L
Hypomagnesemia	1.2 mEq/L – < LLN<br>1.4 mg/dL – < LLN<br>0.58 mmol/L – < LLN	0.9 – < 1.2 mEq/L<br>1.0 - < 1.4 mg/dL<br>0.43 - < 0.58 mmol/L	0.6 – < 0.9 mEq/L<br>0.7 - < 1.0 mg/dL<br>0.28 - < 0.43 mmol/L	< 0.6 mEq/L<br>< 0.7 mg/dL<br>< 0.28 mmol/L
Hypophosphatemia	2.0 mg/dL – < LLN<br>0.63 mmol/L – < LLN	1.5 – < 2.0 mg/dL<br>0.47 - < 0.63 mmol/L	1.0 – < 1.5 mg/dL<br>0.31 - < 0.47 mmol/L	< 1.0 mg/dL<br>< 0.31 mmol/L
Hyperbilirubinemia	> 1.0 – 1.5 × ULN	> 1.5 – 2.5 × ULN	> 2.5 – 5.0 × ULN	> 5.0 × ULN
Blood Urea Nitrogen	1.25 – 2.50 × ULN	> 2.50 – 5.00 × ULN	> 5.00 – 10.00 × ULN	> 10.00 × ULN
Hyperuricemia	> ULN – 10.0 mg/dL<br>> ULN – 0.59 mmol/L	> 10.0 – 12.0 mg/dL<br>> 0.59 – 0.71 mmol/L	> 12.0 – 15.0 mg/dL<br>> 0.71 – 0.89 mmol/L	> 15.0 mg/dL<br>> 0.89 mmol/L
Hypouricemia	1.5 mg/dL – < LLN<br>0.09 mmol/L – < LLN	1.0 – < 1.5 mg/dL<br>0.06 – < 0.09 mmol/L	0.5 - < 1.0 mg/dL<br>0.03 – < 0.06 mmol/L	< 0.5 mg/dL<br>< 0.03 mmol/L
Creatinine	M: > 1.5 - 2.0 mg/dL<br>F: > 1.3 - 1.8 mg/dL<br>M: > 137 – 181 micromol/L<br>F: > 119 – 163 micromol/L	M: > 2.0 - 3.0 mg/dL<br>F: > 1.8 - 2.8 mg/dL<br>M: > 181 – 269 micromol/L<br>F: > 163 – 252 micromol/L	M: > 3.0 - 6.0 mg/dL<br>F: > 2.8 - 5.8 mg/dL<br>M: > 269 – 535 micromol/L<br>F: > 252 – 517 micromol/L	M: > 6.0 mg/dL<br>F: > 5.8 mg/dL<br>M: > 535 micromol/L<br>F: > 517 micromol/L

Continued on following page

Appendix 6. GSI Grading Scale for Severity of Adverse Events and Laboratory Abnormalities (Continued)

HEMATOLOGY (Continued)

	Grade 1	Grade 2	Grade 3	Grade 4
Bicarbonate	16.0 – < 17.0 mEq/L<br>16.0 – < 17.0 mmol/L	11.0 – < 16.0 mEq/L<br>11.0 – < 16.0 mmol/L	8.0 – < 11.0 mEq/L<br>8.0 – < 11.0 mmol/L	< 8.0 mEq/L<br>< 8.0 mmol/L
Triglycerides	—	400 – 750 mg/dL<br>4.52 – 8.48 mmol/L	> 750 – 1,200 mg/dL<br>> 8.48 – 13.56 mmol/L	> 1,200 mg/dL<br>> 13.56 mmol/L
Hypercholesterolemia	> ULN – 300 mg/dL<br>> ULN – 7.78 mmol/L	> 300 – 400 mg/dL<br>> 7.78 – 10.37 mmol/L	> 400 – 500 mg/dL<br>> 10.37 – 12.96 mmol/L	> 500 mg/dL<br>> 12.96 mmol/L
Creatine Kinase	3.0 – < 6.0 × ULN	6.0 – < 10.0 × ULN	10.0 – < 20.0 × ULN	≥ 20.0 × ULN

Continued on following page

Appendix 6.. GSI Grading Scale for Severity of Adverse Events and Laboratory Abnormalities (Continued)

ENZYMES				
	**Grade 1**	**Grade 2**	**Grade 3**	**Grade 4**
AST (SGOT)	1.25 – 2.50 × ULN	> 2.50 – 5.00 × ULN	> 5.00 – 10.00 × ULN	> 10.00 × ULN
ALT (SGPT)	1.25 – 2.50 × ULN	> 2.50 – 5.00 × ULN	> 5.00 – 10.00 × ULN	> 10.00 × ULN
GGT	1.25 – 2.50 × ULN	> 2.50 – 5.00 × ULN	> 5.00 – 10.00 × ULN	> 10.00 × ULN
Alkaline Phosphatase	1.25 – 2.50 × ULN	> 2.50 – 5.00 × ULN	> 5.00 – 10.00 × ULN	> 10.00 × ULN
Total Amylase	> 1.0 – 1.5 × ULN	> 1.5 – 2.0 × ULN	> 2.0 – 5.0 × ULN	> 5.0 × ULN
Lipase	> 1.0 – 1.5 × ULN	> 1.5 – 2.0 × ULN	> 2.0 – 5.0 × ULN	> 5.0 × ULN
Albumin	3.0 g/dL – < LLN<br>30 g/L – < LLN	2.0 – < 3.0 g/dL<br>20 – < 30 g/L	< 2.0 g/dL<br>< 20 g/L	—

URINALYSIS				
	**Grade 1**	**Grade 2**	**Grade 3**	**Grade 4**
Proteinuria (Dipstick)	1+	2–3+	4+	NA
Hematuria (Qualitative)	Microscopic only	Gross, no clots	Gross, with clots or red blood cell casts	Obstructive or required transfusion
Hematuria (Quantitative)	1–10 rbc/hpf	> 10 rbc/hpf	—	—
Glycosuria	1+	2+	3+	4+

Continued on following page

Appendix 6. GSI Grading Scale for Severity of Adverse Events and Laboratory Abnormalities (Continued)

CARDIOVASCULAR				
	**Grade 1**	**Grade 2**	**Grade 3**	**Grade 4**
Cardiac Rhythm		Asymptomatic, transient signs, no Rx required	Recurrent/ persistent; symptomatic Rx required	Unstable dysrhythmia; hospitalization and treatment required
Hypertension	Transient increase > 20 mm/Hg; no treatment	Recurrent, chronic increase > 20 mm/Hg; treatment required	Acute treatment required; outpatient treatment or hospitalization possible	End organ damage or hospitalization required
Hypotension	Transient orthostatic hypotension with heart rate increased by < 20 beat/min or decreased by < 10 mm Hg systolic BP, no treatment required	Symptoms due to orthostatic hypotension or BP decreased by < 20 mm Hg systolic; correctable with oral fluid treatment	Requires IV fluids; no hospitalization required	Mean arterial pressure < 60 mm/Hg or end organ damage or shock; requires hospitalization and vasopressor treatment
Pericarditis	Minimal effusion	Mild/moderate asymptomatic effusion, no treatment	Symptomatic effusion; pain; EKG changes	Tamponade; pericardiocentesis or surgery required
Hemorrhage, Blood Loss	Microscopic/occult	Mild, no transfusion	Gross blood loss; 1–2 units transfused	Massive blood loss; > 3 units transfused

Continued on following page

Appendix 6. GSI Grading Scale for Severity of Adverse Events and Laboratory Abnormalities (Continued)

RESPIRATORY

	Grade 1	Grade 2	Grade 3	Grade 4
Cough	Transient- no treatment	Persistent cough; treatment responsive	Paroxysmal cough; uncontrolled with treatment	—
Bronchospasm, Acute	Transient; no treatment; 70%–80% FEV1 of peak flow	Requires treatment; normalizes with bronchodilator; FEV1 50%–70% (of peak flow)	No normalization with bronchodilator; FEV1 25%–50% of peak flow; or retractions present	Cyanosis: FEV1 < 25% of peak flow or intubation necessary
Dyspnea	Dyspnea on exertion	Dyspnea with normal activity	Dyspnea at rest	Dyspnea requiring oxygen therapy

SKIN

	Grade 1	Grade 2	Grade 3	Grade 4
Mucocutaneous	Erythema; pruritus	Diffuse, maculo papular rash, dry desquamation	Vesiculation or moist desquamation or ulceration	Exfoliative dermatitis, mucous mem-brane involvement or erythema, multiforme or suspected Stevens-Johnson or necrosis requiring surgery
Local reaction (to parenteral injection)	Erythema or tenderness	Induration ≤ 10 mm or phlebitis or inflammation	Induration > 10 mm or ulceration	Necrosis of skin

Continued on following page

Appendix 6. GSI Grading Scale for Severity of Adverse Events and Laboratory Abnormalities (Continued)

GASTROINTESTINAL				
	**Grade 1**	**Grade 2**	**Grade 3**	**Grade 4**
Nausea	Mild or transient; maintains reasonable intake	Moderate discomfort; intake decreased significantly; some activity limited	No significant intake; requires IV fluids	Hospitalization required
Vomiting	1 episode in 24 hours	2–5 episodes in 24 hours	≥ 6 episodes in 24 hours or needing IV fluids	Physiologic consequences requiring hospitalization or requiring parenteral nutrition
Constipation	Requiring stool softener or dietary modification	Requiring laxatives	Obstipation requiring manual evacuation or enema	Obstruction or toxic megacolon
Diarrhea	Mild or transient; 3–4 loose stools/day or mild diarrhea last < 1 week	Moderate or persistent; 5–7 loose stools/day or diarrhea lasting > 1 week	> 7 loose stools/day or bloody diarrhea; or orthostatic hypotension or electrolyte imbalance or > 2L IV fluids required	Hypotensive shock or physiologic consequences requiring hospitalization
Oral Discomfort/ Dysphagia	Mild discomfort; no difficulty swallowing	Some limits on eating/drinking	Eating/talking very limited; unable to swallow solid foods	Unable to drink fluids; requires IV fluids

Continued on following page

Appendix 6. GSI Grading Scale for Severity of Adverse Events and Laboratory Abnormalities (Continued)

NEUROLOGICAL

	Grade 1	Grade 2	Grade 3	Grade 4
Neuro-Cerebellar	Slight incoordination dysdiadochokinesis	Intention tremor, dysmetria, slurred speech; nystagmus	Locomotor ataxia	Incapacitated
Psychiatric	Mild anxiety or depression	Moderate anxiety or depression; therapy required; change in normal routine	Severe mood changes requiring therapy; or suicidal ideation; or aggressive ideation	Acute psychosis requiring hospitalization; or suicidal gesture/attempt or hallucinations
Muscle Strength	Subjective weakness no objective symptoms/ signs	Mild objective signs/ symptoms no decrease in function	Objective weakness function limited	Paralysis
Paresthesia (burning, tingling, etc.)	Mild discomfort; no treatment required	Moderate discomfort; non-narcotic analgesia required	Severe discomfort; or narcotic analgesia required with symptomatic improvement	Incapacitating; or not responsive to narcotic analgesia
Neuro-sensory	Mild impairment in sensation (decreased sensation, e.g., vibratory, pinprick, hot/cold in great toes) in focal area or symmetrical distribution; or change in taste, smell, vision and/or hearing	Moderate impairment (mod decreased sensation, e.g., vibratory, pinprick, hot/cold to ankles) and/or joint position or mild impairment that is not symmetrical	Severe impairment (decreased or loss of sensation to knees or wrists) or loss of sensation of at least mod degree in multiple different body areas (i.e., upper and lower extremities)	Sensory loss involves limbs and trunk; paralysis; or seizures
Hepatic Encephalopathy	Trivial lack of awareness; euphoria or anxiety; shortened attention span; impaired performance of addition	Lethargy or apathy; minimal disorientation from time or place; inappropriate behavior; subtle personality change; impaired performance of subtraction	Somnolence to semi-stupor, but responsive to verbal stimuli; confusion; gross disorientation	Coma (unresponsive to verbal or noxious stimuli)

Continued on following page

Appendix 6. GSI Grading Scale for Severity of Adverse Events and Laboratory Abnormalities (Continued)

MUSCULOSKELETAL

	Grade 1	Grade 2	Grade 3	Grade 4
Arthralgia (joint pain)	Mild pain not interfering with function	Moderate pain, analgesics and/or pain interfering with function but not with activities of daily living	Severe pain; pain and/or analgesics interfering with activities of daily living	Disabling pain
Arthritis	Mild pain with inflammation, erythema or joint swelling – but not interfering with function	Moderate pain with inflammation, erythema or joint swelling – interfering with function, but not with activities of daily living	Severe pain with inflammation, erythema or joint swelling –and interfering with activities of daily living	Permanent and/or disabling joint destruction
Myalgia	Myalgia with no limitation of activity	Muscle tenderness (at other than injection site) or with moderate impairment of activity	Severe muscle tenderness with marked impairment of activity	Frank myonecrosis

Continued on following page

Appendix 6. GSI Grading Scale for Severity of Adverse Events and Laboratory Abnormalities (Continued)

SYSTEMIC				
	**Grade 1**	**Grade 2**	**Grade 3**	**Grade 4**
Allergic Reaction	Pruritus without rash	Localized urticaria	Generalized urticaria; angioedema	Anaphylaxis
Headache	Mild, no treatment required	Transient, moderate; treatment required	Severe; responds to initial narcotic therapy	Intractable; requires repeated narcotic therapy
Fever: oral	37.7–38.5 C or<br>99.9–101.3 F	38.6–39.5 C or<br>> 101.3–103.1 F	39.6–40.5 C or<br>> 103.1–104.9 F	> 40.5 C or<br>> 105 F
Fatigue	Normal activity reduced<br>< 48 hours	Normal activity decreased 25–50%<br>> 48 hours	Normal activity decreased<br>> 50% can't work	Unable to care for self
Other Toxicity Not in Table	Transient or mild discomfort, requiring no limitation or activity, no therapy	Mild-moderate impact on activity, requiring some assistance and medical intervention	Marked impact on activity, requiring some assistance and medical intervention	Complete disability, requiring significant assistance and medical intervention and/or hospitalization

Appendix 7. Summary of Anti-Viral Activity of Tenofovir DF in Chronic Hepatitis B Patients* or Chronic Hepatitis B Patients Co-infected with HIV**

Reference (no.)	N	Study Type	Patient Characteristics at Baseline	Baseline Serum HBV DNA (copies/mL)	TDF Therapy	Change in Serum HBV DNA from Baseline (copies/mL)	HBeAg and HBsAg Loss/Seroconversion
Cecil, 2002* {6850}	TDF: 5	Open-Label	5 pts with chronic hepatitis B (3 with cirrhosis), poor response to LAM (100–300 mg/d)	Not provided	TDF 300 mg/d [treatment duration not given]	–4.8 $\log_{10}$ (mean)	ND
Kulig, 2003* {6559}	TDF+LAM: 5	Open-label	6 pts with chronic hepatitis B and YMDD mutations, treated with LAM 75–150 mg/d × 1–4 y	8.29 $\log_{10}$ (mean, D/C pt censored)	TDF 150 mg/d added to LAM 75–150 mg/d, treated 2–8 mo.	–3.0 $\log_{10}$ (mean, one D/C pt censored)	ND
Kuo, 2004* {6556}	TDF+LAM: 9	Open--label	9 pts with chronic hepatitis B and HBeAg^{+} & anti-HBe negative; treated with LAM median 36 mo, mean 44.9 mo; 4 pts with YMDD mutations	8.28 $\log_{10}$ (mean)	TDF 300 mg/d × 12 mo (range: 6–16 mo) added to LAM regimen	–4.7 $\log_{10}$ (mean)	HBeAg seroconversion or loss in 3/9 pts

Continued on following page

Appendix 7. Summary of Anti-Viral Activity of Tenofovir DF in Chronic Hepatitis B Patients* or Chronic Hepatitis B Patients Co-infected with HIV** (Continued)

Reference (no.)	N	Study Type	Patient Characteristics at Baseline	Baseline Serum HBV DNA (copies/mL)	TDF Therapy	Change in Serum HBV DNA from Baseline (copies/mL)	HBeAg and HBsAg Loss/Seroconversion;
Nery, 2003* {6842}	8	Open-label	8 liver transplant recipients, developed LAM resistance after a median 26 mo treatment with 3TC (range: 10–85 mo)	8.0 $\log_{10}$ (mean)	TDF 300 mg/d for 1–66 mo after LAM resistance developed & continued a median of 4 mo (range :2–12 mo)	–3.0 $\log_{10}$ (mean)	ND
Lucas, 2004* {7459}	TDF+FTC: 7<br>TDF+LAM: 1	Open-label	8 chronic hepatitis B patients with tx failure or suboptimal response on ADV	Median (range): 610,000 copies/mL (19,100–>500,000,000)	TDF + FTC (n = 7)<br>OR<br>TDF + LAM (n = 1)	Median (range): –3.0 (2–6) $\log_{10}$ copies/mL after a median of 6.5 mos.	0/7 patients with positive HBeAg seroconverted
Villeneuve, 2005* {8879}	TDF: 4<br>TDF+LAM: 2	Open-label	HBV mono-infected, 2 pts LAM^R and ADV^R w/N236T, 4 pts LAM^R& suboptimal response to ADV	LAM^R & ADV^R: mean 7.98 $\log_{10}$<br>LAM^R: mean 7.33 $\log_{10}$	Six patients: 2 patients were treated with TDF 300 mg in addition to LAM 100 mg and 4 patients were treated with TDF for up to 7 months	TDF+LAM: –3.82 $\log_{10}$ after 6–7 months<br>TDF > 1log drop after 1 month and > 2 $\log_{10}$ drop after 3 months	ND

Continued on following page

Appendix 7. Summary of Anti-Viral Activity of Tenofovir DF in Chronic Hepatitis B Patients* or Chronic Hepatitis B Patients Co-infected with HIV** (Continued)

Reference (no.)	N	Study Type	Patient Characteristics at Baseline	Baseline Serum HBV DNA (copies/mL)	TDF Therapy	Change in Serum HBV DNA from Baseline (copies/mL)	HBeAg and HBsAg Loss/Seroconversion;
Im, 2005* {8880}	TDF+LAM: 8<br>TDF+FTC: 5<br>ADV+FTC: 4<br>ADV+LAM:13	Retrospective Analysis	HBV mono-infected, 20/30 $HBeAg^+$ and LAM or ADV experienced; patients had to have received combination therapy for at least 6 months	5.14 log_{10} (n = 29)	Patients received either TDF in combination with LAM (n = 8) or FTC (n =5) or ADV in combination with LAM (n = 13) or FTC (n = 4);median time on therapy 14 mo	TDF: 38% < 2.2 log_{10} (LOD) at 6 mo & 80% < LOD at 12 mo<br>ADV:52% < LOD at 6 mo & 71% < LOD at 12 mo	2 patients seroconverted to anti-HBe
van Bommel, 2005* {8881}	TDF: 14	Observational study	HBV mono-infected, LAM^R (4 patients with YMDD) and ADV suboptimal responders (HBV DNA decline < $3log_{10}$ or HBV DNA > 10^6 copies/mL in the absence of genotypic resistance)	Mean $6.6log_{10}$	14 patients received TDF300 mg for a period of 6 to 14 months	At 6 months mean decrease in HBV DNA of $3.9log_{10}$	13/14 patients < LOD at 4 months<br>2 patients lost HBeAg

Continued on following page

Appendix 7. Summary of Anti-Viral Activity of Tenofovir DF in Chronic Hepatitis B Patients* or Chronic Hepatitis B Patients Co-infected with HIV** (Continued)

Reference (no.)	N	Study Type	Patient Characteristics at Baseline	Baseline Serum HBV DNA (copies/mL)	TDF Therapy	Change in Serum HBV DNA from Baseline (copies/mL)	HBeAg and HBsAg Loss/Seroconversion
Neff, 2004* {7877}	TDF+LAM: 8	Retrospective Chart evaluation	TDF added to LAM[R] patients post-liver transplantation; 7 HBV monoinfected and 1 HBV/HIV co-infected;8 HBeAg+ prior to LAM	Range: 163 to > 5 × 10^8 copies/mL by PCR or HBV DNA hybridization	TDF 300 mg was added to the LAM regimen in 8 patients 1–66 months after LAM resistance diagnosed and continued for 14–26 mo	7/8 patients undetectable by PCR or DNA hybridization techniques	ND
Neff, 2004* {7735}	TDF+LAM: 5	Retrospective Chart evaluation	TDF added to LAM[R] patients 1–25 months post liver transplantation; 1 patient HBV/HIV co-infected and 4 patients HBV monoinfected.	ND	5 Patients with HBV recurrence post transplantation received TDF 300 mg in addition to LAM 150 mg	2 patients were undetectable and 3 patients ≤ 106 copies/mL after 12–16 mo treatment	3/4 patients seroconverted to anti-HBe;

Continued on following page

Appendix 7. Summary of Anti-Viral Activity of Tenofovir DF in Chronic Hepatitis B Patients* or Chronic Hepatitis B Patients Co-infected with HIV** (Continued)

Reference (no.)	N	Study Type	Patient Characteristics at Baseline	Baseline Serum HBV DNA (copies/mL)	TDF Therapy	Change in Serum HBV DNA from Baseline (copies/mL)	HBeAg and HBsAg Loss/Seroconversion
Dore, 2004** {6586},{6843}	TDF+LAM: 5<br>LAM: 6	Double-blind, controlled (Gilead Sciences Study 903)	600 ART-naive HIV−1+ pts; 23 of 599 pts HBsAg+, 11 of whom had HBV ≥ 6 $\log_{10}$ copies/mL and samples for analysis; none of the 11 pts had YDMM mutations, 10 of whom were HBeAg+; TDF arm, n = 5; d4T arm, n = 6	For 10 of the 11 HBsAg+pts, HBV DNA 10^8—10^9 copies/mL (mean)<br>TDF arm: HBV DNA 8.3 $\log_{10}$<br>d4T arm: HBV DNA 8.9 $\log_{10}$	TDF arm: TDF 300 mg/d × 144 wk in combination with 3TC & EFV<br><br>d4T arm: d4T/3TC/EFV	TDF arm: HBV DNA −4.5 $\log_{10}$ (mean) at wk 144, 4 of 5 pts with HBV < LOD at wk 48<br>d4T arm: HBV DNA −1.9 $\log_{10}$ (mean) at wk 144, 1 of 6 pts with HBV < LOD at wk 48	TDF arm: 2 of 4 pts had HBeAg seroconversion<br>d4T arm: 1 of 6 pts had HBeAg seroconversion
Dore, 2004, Cheng, 2002** {6586}	TDF: 10<br>PLC: 2	Double-blind, placebo-controlled intensification study (Gilead Sciences Study 907	552 treatment-experienced HIV−1+ pts; of 539 pts screened, 23 were HBsAg+, 12 of whom had HBV > 6 $\log_{10}$ and samples for analysis, all 12 were HBeAg+, 7 of whom had 3TC resistance mutations (6 in arm 1, 1 in arm 2)	TDF Arm: HBV 8.6 $\log_{10}$ (mean)<br>Placebo Arm: HBV 8.1 $\log_{10}$ (mean)	TDF Arm: TDF 300 mg/d × 48 w k added to prior regimen<br>Placebo Arm: placebo added to prior regimen to wk 24, cross-over to TDF through wk 48	TDF Arm : −4.9 $\log_{10}$ at wk 24, −4.5 $\log_{10}$ at wk 48, (mean), similar ↓ for pts with wild-type or 3TC resistant virus at wk 24<br>Placebo Arm: +1.2 $\log_{10}$ at wk 24	TDF Arm: HBeAg seroconversion in 1 pt, 1 pt lost HBeAg but remained anti-HBe negative; ALT normalized in 2 of 8 pts with ↑ ALT at BL; 3 SAEs

Continued on following page

Appendix 7. Summary of Anti-Viral Activity of Tenofovir DF in Chronic Hepatitis B Patients* or Chronic Hepatitis B Patients Co-infected with HIV** (Continued)

Reference (no.)	N	Study Type	Patient Characteristics at Baseline	Baseline Serum HBV DNA (copies/mL)	TDF Therapy	Change in Serum HBV DNA from Baseline (copies/mL)	HBeAg and HBsAg Loss/Seroconversion
Picketty, 2004** {6122}	TDF: 30<br>TDF+LAM: 85	Open-label	Retrospective analysis of 115 pts (109 male) in TECOVIR study; 95 pts HBV DNA +, 74 of whom were HBeAg+	8.09 log_{10} (median)	TDF 300 mg/d × 8 mo (median) added to prior regimen	HBV –3.94 log_{10} (median) after a median of 9 mo; –4.39 log_{10} for pts HBeAg+ at BL	7 pts became HBeAg–, 3 of whom seroconverted to anti-HBe+
Benhamou 2006** {8805}	TDF: 14<br>TDF+LAM: 52	Retrospective Chart evaluation	Subset of the HBV/HIV Co-infected Tecovir cohort with baseline serum HBV DNA > 2.3log_{10} copies/mL (LAM^R); 42 patients with YMDD mutations; 54 HBeAg positive and 11 HBeAg negative	8.17log_{10} HBeAg+<br>4.83 log_{10} HBeAg-	65 patients were identified as having received TDF for at least 6 months and 52 patients were also receiving LAM; median follow-up period was 12 mo	HBeAg Positive: -4.56 log10 copies/mL and 16/54 patients with HBV DNA < LOD;<br>HBeAg Negative: -2.53 log10copies/mL and 9/11 patients with HBV DNA < LOD;	HBeAg Positive:<br>4 patients lost HBeAg and 2 patients seroconverted to anti-HBe

Continued on following page

Appendix 7. Summary of Anti-Viral Activity of Tenofovir DF in Chronic Hepatitis B Patients* or Chronic Hepatitis B Patients Co-infected with HIV** (Continued)

Reference (no.)	N	Study Type	Patient Characteristics at Baseline	Baseline Serum HBV DNA (copies/mL)	TDF Therapy	Change in Serum HBV DNA from Baseline (copies/mL)	HBeAg and HBsAg Loss/Seroconversion
van Bommel, 2004** {7213}	TDF: 25<br>TDF+LAM: 10<br>ADV: 18	Active-control	53 pts with LAM resistance mutations;<br>TDF arm: n = 35, 21 pts HIV-1+/HBV+, 5 pts immunosuppressed HBV after kidney transplant, 9 pt chronic HBV; 31 pts HBeAg+, all pts D/C LAM except 10 HIV–1+/HBV+ pts.<br>ADV arm: n = 18, all pts with chronic HBV; all pts D/C LAM , 16 pts HBeAg+	TDF arm: 9.4 $\log_{10}$<br>ADV arm: 8.5 $\log_{10}$<br>(mean)	18 pts treated with TDF 300 mg/d × for 72–130 wks; 18 pts treated with ADV 10 mg/d × for 60–80 wks;	TDF arm: –5.2 $\log_{10}$ at wk 24 (mean; 100% of pts HBV < 400 copies/mL at wk 48, –5.5 $\log_{10}$ at wk 48<br><br>ADV arm: –2.6 $\log_{10}$ at wk 24 (mean), 44% of pts HBV DNA < 400 copies/mL at Week 48, –2.8 $\log_{10}$ at wk 48	TDF arm: 5 pts had HBsAg loss, 11/31 pts had HBeAg loss<br><br>ADV arm: 1/16 pts had HBsAg loss, 3/16 pts had HBeAg loss
Peters, 2006** {10408}	TDF: 27<br>ADV: 25	Randomized, Double-blind	52 HBV/HIV co-infected patients receiving stable HIV tx ; serum HBV DNA ≥ 100,000 copies/mL; 94% LAMR, 86% HBeAg+	TDF arm: mean 9.45 $\log_{10}$<br>ADV arm: mean 8.85 $\log_{10}$	52 patients total:<br>27 patients TDF 300 mg + placebo OR<br>25 patients ADV 10 mg + placebo<br>For up to 96 weeks	Mean $DAVG_{48}$:<br>TDF arm: -4.44 $\log_{10}$<br>ADV arm: -3.21 $\log_{10}$	11 patients (6 TDF, 5 ADV) with ALT flares but no decompensation; 4 D/C in each group, including 2 deaths (HCC on ADV, unknown cause on TDF)

Continued on following page

Appendix 7. Summary of Anti-Viral Activity of Tenofovir DF in Chronic Hepatitis B Patients* or Chronic Hepatitis B Patients Co-infected with HIV** (Continued)

Reference (no.)	N	Study Type	Patient Characteristics at Baseline	Baseline Serum HBV DNA (copies/mL)	TDF Therapy	Change in Serum HBV DNA from Baseline (copies/mL)	HBeAg and HBsAg Loss/Seroconversion
Gilleece, 2004** {7208}	TDF: 9<br>TDF+LAM: 30	Open-label	39 pts (38 males); all HBeAg+; at BL, 30 pt were treated with LAM for a median 71 wks; 18/32 pts with YMDD mutation; median ALT 62 mmol/L	HBV 7.84 $\log_{10}$ (median)	TDF 300 mg/d × 96 wk was part of or added to prior regimen	HBV –3.8 $\log_{10}$ (median); at wk 24, which was at the LOD; median HBV remained < LOD through wk 96	6 pts (3 with LAM resistance mutations at baseline) seroconverted to anti-HBe+; 9 pts became HBeAg– wk 24–72
Jain, 2007** {10612}	LAM: 15<br>TDF+LAM: 10<br>LAM, followed by TDF+LAM: 20	Open-label; retrospective	45 HBV/HIV co-infected patients; 78% of patients harbored genotype A virus	detectable HBV DNA @ baseline or year 1	1) 3TC 150 mg BID (n = 15)<br>2) 3TC + TDF 300 mg QD (n = 10)<br>3) 3TC for ≥ 1 yr followed by 3TC + TDF (n = 20)	After one year (average):<br>1) –2.6 $\log_{10}$<br>2) –4.5 $\log_{10}$<br>3) –2.7 $\log_{10}$	1) 29/0%<br>2) 40%/20%<br>3) 19%/19%

Continued on following page

Appendix 7. Summary of Anti-Viral Activity of Tenofovir DF in Chronic Hepatitis B Patients* or Chronic Hepatitis B Patients Co-infected with HIV** (Continued)

Reference (no.)	N	Study Type	Patient Characteristics at Baseline	Baseline Serum HBV DNA (copies/mL)	TDF Therapy	Change in Serum HBV DNA from Baseline (copies/mL)	HBeAg and HBsAg Loss/Seroconversion
Gozlan, 2004** {7210}	TDF:12<br>TDF+LAM: 16	Open-label	28 pts; 25 pts treated with LAM for a median 55 mo; 24 pts HBeAg+; 16/22 pts with LAM resistance mutation; ALT median of 125 nmol/mL	7.3 $\log_{10}$ HBV (median)	TDF 300 mg/d × 610 d (median); 16 pts continued LAM	HBV –4.9 $\log_{10}$ (mean) at 12 mo; 18 pts with HBV < LOD (200 copies/mL) at follow-up	4 pts with HBe seroconversion at follow-up
Park, 2002** {6849}	TDF+LAM: 21	Open-label	21 male pts treated with LAM (median 36 mo) & famciclovir (median 18 mo), all pts HBeAg +	HBV 8.6 $\log_{10}$ (median)	TDF 300 mg/d × 24 wk added to prior regimen	HBV –4.3 $\log_{10}$ (median ↓ vs. BL)	1 pt seroconverted to anti-HBe+

Continued on following page

Appendix 7. Summary of Anti-Viral Activity of Tenofovir DF in Chronic Hepatitis B Patients* or Chronic Hepatitis B Patients Co-infected with HIV** (Continued)

Reference (no.)	N	Study Type	Patient Characteristics at Baseline	Baseline Serum HBV DNA (copies/mL)	TDF Therapy	Change in Serum HBV DNA from Baseline (copies/mL)	HBeAg and HBsAg Loss/Seroconversion
Nelson, 2003** {4199}	TDF: 5<br>TDF+LAM: 15	Open-label	20 male pts; 15 pts treated with LAM for a median of 138 wk; 11/15 pts with HBV resistance mutations (10 with YMDD, 1 with YIDD); ALT 96 IU/mL (median)	HBV 1.815×10^8 GEq/mL (median)	TDF 300 mg/d × 52 wk added to prior regimen	HBV –4 $\log_{10}$ (median); ↓ HBV initially faster in pts with HBV mutations than without; 13 pts HBV < LOD at wk 52	2 pts seroconverted at wk 24 & 5 pts at wk 52, 3 of whom had HBV resistance mutations
Gomez, 2004** {7209}	TDF: 2<br>TDF+LAM:14	Open-label	16 males with active HBV infection, 14 treated with LAM at BL, HBV+, HBsAg+ ≥ 6 mo	> 1×10^6 HBV (median)	TDF 300 mg/d × 24 wk added to prior regimen; median follow-up 10 mo (range 4–8 mo)	HBV < LOD (200 copies/mL) at 12 mo	ND
Montessori, 2004** {7211}	TDF: 1<br>TDF+LAM:15		16 pts, $HBsAg^+$ & $HBeAg^+$, 15 pts treated with LAM (150 mg BID); 9 pts with LAM resistance mutations	HBV 7.75 $\log_{10}$ (median)	TDF 300 mg/d × 12 wk added to prior regimen	–3.17 $\log_{10}$ (median); 5/16 pts with HBV < LOD at 12 wk	ND

Continued on following page

Appendix 7. Summary of Anti-Viral Activity of Tenofovir DF in Chronic Hepatitis B Patients* or Chronic Hepatitis B Patients Co-infected with HIV** (Continued)

Reference (no.)	N	Study Type	Patient Characteristics at Baseline	Baseline Serum HBV DNA (copies/mL)	TDF Therapy	Change in Serum HBV DNA from Baseline (copies/mL)	HBeAg and HBsAg Loss/Seroconversion
Benhamou, 2003** {4630}	TDF+LAM:12	Open-label	12 male pts, regimen included LAM (150 mg BID); pts HBV+ for median 30 mo, 10 pts with HBV resistance mutations	HBV 7.4 $\log_{10}$ (mean)	TDF 300 mg/d × 24 wk added to prior regimen	–3.83 $\log_{10}$ (mean) at wk 24	no change in HBeAg or seroconversion to anti-HBe
Nùñez, 2002** {3940}	TDF+LAM:12	Open-label	12 pts (11 males), treated with LAM for ≥ 24 wk (median exposure 84 wk); 7/11 pts with LAM resistance mutations; all pts HBV+, 9 pts HBeAg +	HBV 6.54 $\log_{10}$ (mean)	TDF 300 mg/d × 24 wk added to prior regimen	HBV –3.78 $\log_{10}$ (median); 7 pts had HBV < LOD	1 pt lost HBeAg– at wk 12, and cleared HBsAg at wk 24
Marcellin, 2003** {6860}	TDF+LAM:10	Open-label	10 pts, all treated with LAM, 9 pts HBeAg+ and with LAM resistance mutations	not clearly stated	TDF 300 mg/d × 12 mo added to prior regimen	–4.55 $\log_{10}$ (median); at 12 mo; 3 pts with HBV DNA < LOD (200 copies/mL)	no HBeAg loss or seroconversion

Continued on following page

Appendix 7. Summary of Anti-Viral Activity of Tenofovir DF in Chronic Hepatitis B Patients* or Chronic Hepatitis B Patients Co-infected with HIV** (Continued)

Reference (no.)	N	Study Type	Patient Characteristics at Baseline	Baseline Serum HBV DNA (copies/mL)	TDF Therapy	Change in Serum HBV DNA from Baseline (copies/mL)	HBeAg and HBsAg Loss/Seroconversion
Ristig, 2002** {4121}	TDF: 1<br>TDF+LAM/FTC: 5	Open-label	6 pt; treated with 3TC or FTC for median of 38 mo, all pts had detectable HBsAg & HBeAg; 4 pts had cirrhosis; ALT 53 U/L (median)	7.95 $\log_{10}$ (median)	TDF 300 mg/d × up to 24 wk (median) added to prior regimen	-3.1 $\log_{10}$ (median) at Week 12 and –4.35 $\log_{10}$ (median) at Week 24, 2 pts < LOD at Week 24;	no pt developed new anti-HBe
Bruno, 2003** {4376}	TDF+LAM: 5	Open-label	5 males with YMDD mutation; treated with 3TC (150 mg BID) for 42–65 mo.; all 5 pts HBeAg– & anti-HBe+	5.1 $\log_{10}$ (mean)	TDF 300 mg/d × 4 wk added to prior regimen	HBV < LOD at wk 4; HBV –2.42 $\log_{10}$ (mean)	ND
Schildgen, 2004** {7468}	TDF: 3	Open-label	3 HBV/HIV co-infected patients (HBeAg+) with genotype A virus and the same 3TC resistance mutations (L180M, YVDD)	Approx. 106–107	TDF replaced ADV; regimens not mentioned; duration of TDF treatment ≥ 6 months	At least -3 $\log_{10}$ in each patient	ND

Continued on following page

Appendix 7. Summary of Anti-Viral Activity of Tenofovir DF in Chronic Hepatitis B Patients* or Chronic Hepatitis B Patients Co-infected with HIV** (Continued)

Reference (no.)	N	Study Type	Patient Characteristics at Baseline	Baseline Serum HBV DNA (copies/mL)	TDF Therapy	Change in Serum HBV DNA from Baseline (copies/mL)	HBeAg and HBsAg Loss/Seroconversion
Norris, 2004** {7833}	TDF+LAM: 2	Retrospective cohort of liver transplantation patients	2 HBV/HIV co-infected patients undergoing liver transplant	HBV DNA negative prior to liver transplant	2 HBV/HIV patients received TDF + LAM + HBIG post liver transplant; 1 pt received LAM+TDF pre transplant	Not available however no patient experienced HBV recurrence	ND
Mauss, 2005** {7790}	TDF:24<br>TDF+LAM: 8	Open-label	32 HBeAg positive HBV/HIV Co-infected; 24 Lam[R] patients	7.69 $\log_{10}$ (combo)<br>8.27 $\log_{10}$ (mono)	8 naïve patients to TDF+LAM received combination therapy and compared to 24 LAM[R] patients who received TDF monotherapy for 12 mo	TDF+LAM: -4.35 log10 ; 6/8 < LOD<br>TDF: -5.27 log10; 19/24 < LOD<br>(at 1 year)	TDF+LAM: 1/8 patients with HBeAg loss and 0/8 patients with HBsAg loss<br>TDF: 6/24 patients with HBeAg loss and 2/24 with HBsAg loss
Schildgen, 2005 ** {8477}	TDF: 6	Observational	3 HBV/HIV Co-infected and 3 HBV mono-infected patients	patients with no virologic response to ADV after 6 months of treatment	LAM[R] HBV/HIV co-infected patients with a suboptimal response to ADV were treated with TDF	significant drop in viral load was observed (no specifics provided)	ND

Continued on following page

Appendix 7. Summary of Anti-Viral Activity of Tenofovir DF in Chronic Hepatitis B Patients* or Chronic Hepatitis B Patients Co-infected with HIV** (Continued)

Reference (no.)	N	Study Type	Patient Characteristics at Baseline	Baseline Serum HBV DNA (copies/mL)	TDF Therapy	Change in Serum HBV DNA from Baseline (copies/mL)	HBeAg and HBsAg Loss/Seroconversion
van Bommel 2005 {7781}	TDF: 38<br>ADV: 28<br>LAM: 116	Retrospective Analysis	34/38 HBeAg+ of whom 24 were HBV/HIV co-infected received TDF; 28 received ADV (24 HBeAg+) and 116 received LAM (58 HBeAg+); Patients treated with TDF or ADV were LAM^R	All patients > 2 × 10^6 copies/mL at baseline	182 HBV infected patients received either TDF (38 patients), ADV (28 patients) or lamivudine (116 patients) for a mean duration of 19 to 26 months;	At mo 12: 92% (TDF), 43% (ADV) and 56% (LAM) < LOD<br>At mo 24: 100% (TDF), 64% (ADV), 66% (LAM) < LOD	41% (TDF), 17% (ADV) and 33% (LAM) lost HBeAg and 8% (TDF), 3.6% (ADV) and 3% (LAM) lost HBsAg.
Mauss, 2005 ** {8876}	TDF: 46<br>TDF+LAM: 23	Open-label	69 HBV/HIV Co-infected, LAM^R	TDF+LAM:7.77 $\log_{10}$<br>TDF: 8.08 $\log_{10}$	23 patients received TDF + LAM and 46 LAM^R patients received TDF only for a median of 24 mo	Both the TDF+LAM and TDF regimens had a median HBV DNA <3$\log_{10}$ at 12 and 24 months;<br>sustained HBV DNA < LOD in 19/23 (TDF+LAM) and 38/46 (TDF)	HBeAg loss in 7/22 (TDF+LAM) and 11/44 (TDF)<br>4% in either arm with HBsAg loss
Klaussen, 2005** {8877}	TDF: 21	Retrospective Analysis	HBV/HIV Co-infected, 10 LAM^R (≥ 2 $\log_{10}$ increase in HBV DNA while receiving LAM)	Mean 1.7 × 10^{12} IE/mL (n = 21)<br>1.2 × 10^{12}IE/mL (LAM^R; n = 10)	21 patients were treated with TDF for a mean of 27 mo	230 IE/mL at 24 months (n = 15)<br>[8 pts with LAM^R 386 IE/mL at 24 mo]	ND

Continued on following page

Appendix 7. Summary of Anti-Viral Activity of Tenofovir DF in Chronic Hepatitis B Patients* or Chronic Hepatitis B Patients Co-infected with HIV** (Continued)

Reference (no.)	N	Study Type	Patient Characteristics at Baseline	Baseline Serum HBV DNA (copies/mL)	TDF Therapy	Change in Serum HBV DNA from Baseline (copies/mL)	HBeAg and HBsAg Loss/Seroconversion
van Bommel**, 2005 {8875}	TDF: 35<br>ADV: 53	Retrospective evaluation	TDF:32/35 HBeAg positive; 24/35 HBV/HIV co-infected; all LAM[R] with YMDD<br>ADV:49/53 HBeAg positive;32 LAM[R] with YMDD	All patients > 20 × 10^5 copies/mL at baseline (mean/median not provided)	A total of 88 patients were treated with either TDF 300 mg (n = 35) or ADV 10 mg (53) for a mean duration of 33 and 24 months	At mo 12, 94% (TDF) and 32% (ADV) were < 400 copies/mL (LOD).<br>At mo 18 and 24 100% of the TDF patients were < LOD; 35% and 49% of the ADV patients were < LOD	After a mean duration of 15 mos HBeAg loss was observed in 51% (TDF) and 21% (ADV) of the patients. HBsAg loss was observed in 12% (TDF) and 6% (ADV) of the patients after a mean of 20 and 19 months.
Lacombe 2005** {7792}	TDF: 12<br>TDF+LAM: 16	Open-Label Prospective study	HBV/HIV co-infected; 24/28 HBeAg positive and 4 patients also co-infected with HCV	Mean 7.48 log_{10}	12 patients were treated with TDF and 16 treated with a combination of TDF+LAM for a median duration of 517 days	Mean -4.6log_{10}<br>21/28 < 200 copies/mL (mean 71 weeks)	4 patients seroconverted to anti-HBe

Continued on following page

Appendix 7. Summary of Anti-Viral Activity of Tenofovir DF in Chronic Hepatitis B Patients* or Chronic Hepatitis B Patients Co-infected with HIV** (Continued)

Reference (no.)	N	Study Type	Patient Characteristics at Baseline	Baseline Serum HBV DNA (copies/mL)	TDF Therapy	Change in Serum HBV DNA from Baseline (copies/mL)	HBeAg and HBsAg Loss/Seroconversion
van der Eijk 2005** {8455}	TDF+LAM: 11	Open-Label Prospective viral dynamic study	6 HBV mono-infected and 5 HBV/HIV co-infected; all LAM[R] and 9 patients with YMDD; 10 HBeAg positive	8.62 log_{10}	11 patients added TDF to a LAM regimen after a median 176 weeks exposure to LAM	24 weeks: mean HBV DNA log decline of 4.95 log_{10} for 10/11 patients;<br>5/10 < 1000 copies/ml (LOD)	1/10 patient lost HBeAg
Sheldon, 2005** {8380}	TDF+LAM: 43	Retrospective genotypic evaluation	HBV/HIV Co-infected patients with detectable HBV DNA ; 35 patients HBeAg+	Mean 4.6 log_{10}	43 patients with detectable HBV DNA after being treated for at least 6 months with TDF + LAM (mean 11.2 months TDF and 35.3 months LAM) were genotyped	ND	ND
Woolley, 2004** {7176}	TDF+LAM: 1	Case Report	HBV/HIV Co-infected patient	2060 pg/mL	1 patient was treated with TDF in combination with LAM	Reduction in HBV DNA to 1.5 pg/mL;	ND

Continued on following page

Appendix 7. Summary of Anti-Viral Activity of Tenofovir DF in Chronic Hepatitis B Patients* or Chronic Hepatitis B Patients Co-infected with HIV** (Continued)

Reference (no.)	N	Study Type	Patient Characteristics at Baseline	Baseline Serum HBV DNA (copies/mL)	TDF Therapy	Change in Serum HBV DNA from Baseline (copies/mL)	HBeAg and HBsAg Loss/Seroconversion
Stephan, 2005** {8809}	TDF: 14<br>TDF+LAM: 17	Retrospective Cohort evaluation	HBV/HIV Co-infected Frankfurt HIV cohort; 24 patients were LAM experienced (15/20 patients with baseline HBV sequence had YMDD mutation)	20/31 patients with high replicative HBV, i.e., > 6 log_{10} at baseline (2 pts HBeAg neg) and 11 patients < 6 log_{10} at baseline	31 patients treated for at least 48 weeks with TDF	High replicative group: median decline of 5.37 log_{10} at 48 weeks; 14/20 < 200 copies/mL (LOD)<br>Low replicative group: 11/11 < LOD at 48 weeks	HBeAg loss in 2 patients and 1 patient became HBsAg negative
Bani-Sadr 2004** {7822}	TDF+LAM: 6	Prospective Pilot study	HBV/HIV Co-Infected patients; 1 patient also HCV infected and 1 patient although positive for HBV DNA (3.43 log_{10} copies/mL) was negative for HBsAg	4.45 log_{10}	6 patients treated with a combination of TDF + LAM for at least 96 weeks	5/6 < 200 copies/mL (LOD) at wk 48; 6/6 < LOD at wk 96	2/3 patients lost HBeAg and 1 pt seroconverted to anti-HBe by Week 48<br>3/5 patients lost HBsAg with one patient seroconverting to anti-HBs by wk 48

Continued on following page

Appendix 7. Summary of Anti-Viral Activity of Tenofovir DF in Chronic Hepatitis B Patients* or Chronic Hepatitis B Patients Co-infected with HIV** (Continued)

Reference (no.)	N	Study Type	Patient Characteristics at Baseline	Baseline Serum HBV DNA (copies/mL)	TDF Therapy	Change in Serum HBV DNA from Baseline (copies/mL)	HBeAg and HBsAg Loss/Seroconversion
Trimoulet 2003** {8807}	TDF: 4<br>TDF+LAM: 80	Retrospective Analysis	HBV/HIV Co-infected patients; 71 patients with detectable HBV DNA at baseline (69 of these patients on LAM)	7.6 log_{10} (for pts with detectable HBV DNA	84 patients were identified as having received TDF for a median of 9 mo; 80 pts received TDF+LAM and 4 pts received TDF	3.6 log_{10} decline from baseline in HBV DNA<br>After 10 mo 37% < LOD (200 copies/mL)	ND
van Bommel 2006* {9241}	TDF: 20	Retrospective analysis	HBV mono-infected patients; 19/20 HBeAg+; 6/20 LAM^R with genotypic mutations; suboptimal response to ADV	Mean 6.59 log_{10} copies/mL	20 patients identified as HBsAg+ and having received TDF for a median of 12 months (range 3–24 months)	Median decrease in HBV DNA was 3.8 log_{10} copies/mL; 19/20 pts < 400 copies/mL	16/20 pts with normal ALT; 4 patients lost HBeAg;1 patient seroconverted to anti-HBs
Nunez 2005** {8806}	TDF: 46<br>LAM: 29	Retrospective analysis	HBV/HIV co-infected; 39 HBeAg+ and 40 HBeAg-	ND	79 patients were identified as HBsAg+ and having received TDF (58%) or LAM (37%); median time on trt was 39 and 52 mo, respectively	82% HBeAg- pts and 58% HBeAg+ pts < LOD at the end of follow-up	10/36 patients with HBeAg loss and 10/75 patients with HBsAg loss

Continued on following page

Appendix 7. Summary of Anti-Viral Activity of Tenofovir DF in Chronic Hepatitis B Patients* or Chronic Hepatitis B Patients Co-infected with HIV (Continued)**

Reference (no.)	N	Study Type	Patient Characteristics at Baseline	Baseline Serum HBV DNA (copies/mL)	TDF Therapy	Change in Serum HBV DNA from Baseline (copies/mL)	HBeAg and HBsAg Loss/Seroconversion
Nelson 2006**<br>{8893}	TDF: 22<br>TDF+LAM: 17<br>LAM: 20	Multicenter, open-label, randomized	HBV/HIV co-infected; 32 LAM experienced and 27 LAM naive	LAM experienced:<br>12 TDF: 7.7 log_{10}<br>9 LAM: 8.2 log_{10}<br>11 TDF+LAM: 7.7log_{10}<br>LAM naïve:<br>10 TDF: 8.2 log_{10}<br>11LAM: 7.5 log_{10}<br>6 TDF+LAM: 8.2log_{10}	59 patients randomized to either TDF, LAM or TDF+LAM	Median change in HBV DNA (DAVG 24weeks)<br>LAM experienced:<br>TDF: -3.41 log_{10}<br>LAM: -0.82 log_{10}<br>TDF+LAM: -3.93log_{10}<br>LAM naïve:<br>TDF: -4.66 log_{10}<br>LAM: -3.31 log_{10}<br>TDF+LAM: -5.03log_{10}<br><br>At 24 weeks proportion < LOD(400co/mL):<br>LAM experienced:<br>TDF: 2/12<br>LAM:0/9<br>TDF+LAM:4/11<br>LAM naïve:<br>TDF:4/10<br>LAM:4/11<br>TDF+LAM:3/6	ND

Continued on following page

Appendix 7. Summary of Anti-Viral Activity of Tenofovir DF in Chronic Hepatitis B Patients* or Chronic Hepatitis B Patients Co-infected with HIV** (Continued)

Reference (no.)	N	Study Type	Patient Characteristics at Baseline	Baseline Serum HBV DNA (copies/mL)	TDF Therapy	Change in Serum HBV DNA from Baseline (copies/mL)	HBeAg and HBsAg Loss/Seroconversion
van Bommel 2007* {11915}	TDF: 108	Multicenter retrospective cohort analysis	HBV mono-infected;<br>HBV DNA > 10^4 copies/mL<br>Prior treatment experience:<br>LAM: 16<br>ADV: 7<br>Sequential LAM and ADV: 58<br>Add-on combination with LAM+ADV: 18<br>ETV: 2<br>Sequential LAM and ETV: 1<br>Treatment naïve: 6	$6.6 \pm 1.4\ log_{10}$	≥ 6 months of TDF therapy	Median change in HBV DNA:<br>6 months: - 3.6 log_{10}<br>12 months: - 4.0 log_{10}<br>18 months: - 4.1 log_{10}<br><br>Proportion < 400 copies/mL:<br>12 months: 84/93 (90%)	12 months:<br>HbeAg seroconversion: 12/68 (18%)<br>HbsAg loss: 1/93 (1.1%)

Abbreviations: HBV, hepatitis B virus; 3TC, lamivudine; BID, twice-daily; TDF, tenofovir DF; D/C, discontinued; AE adverse event; pt, patient; BL, baseline; ALT, alanine aminotransferase, CREAT, serum creatinine; ETV, entecavir; HBsAg, hepatitis B surface antigen; HBeAg, hepatitis B e antigen; anti-HBs, antibody to HBsAg; anti-HBe, antibody to HBeAg; ALT, alanine aminotransferase; ULN upper limit of normal; AFP, alpha-fetoprotein; LOD, limit of detection

Appendix 8. Lactic Acidosis Guidelines

Lactic acidosis and severe hepatomegaly with steatosis, including fatal cases, have been reported with the use of nucleoside analogues alone or in combination with other antiretrovirals. A majority of these cases have been in women. Obesity and prolonged nucleoside exposure may be risk factors; however, cases have also been reported in subjects with no known risk factors.

Guidelines for management of symptomatic hyperlactatemia and asymptomatic hyperlactatemia are outlined in Section A and B below and are derived from the AIDS Clinical Trials Group (ACTG) Lactic Acidosis Guidelines. Section C outlines venous lactate collection techniques.

Section A. Symptomatic Hyperlactatemia

Symptomatic hyperlactatemia is defined as a clinical suspicion of hyperlactatemia characterized by new, otherwise unexplained and persistent (≥ 2 weeks) occurrence of 1 or more of the following symptoms:

–Nausea and vomiting
–Abdominal pain or gastric discomfort
–Abdominal distention
–Increased LFTs
–Unexplained fatigue
–Dyspnea

AND

Venous lactate level greater than twice the upper normal limit (ULN) confirmed by repeat venous lactate analysis within 1 week and, if persistently elevated, arterial lactate with blood gas analysis.

If the repeat venous lactate is elevated confirmation with an arterial lactate specimen and arterial blood gas (pH, PO_2, PCO_2, bicarbonate, oxygen saturation) should be performed within 48 hours. If the arterial specimen contains lactate at a level more than two times the upper limit of normal, the patient should be discontinued from the study and alternative therapy instituted. Subjects should be monitored weekly until signs and symptoms resolve. Hyperlactatemia should be followed until levels return to below two times the ULN and the patient.

An elevated anion gap in a patient with metabolic acidosis suggests the diagnosis of lactic acidosis. It can be suspected when the sum of cations minus the sum of anions $[(Na^+ + K^+) - (Cl^- + HCO_3^-)]$ exceeds 18 mEq/L (18 mmol/L) in the absence of other causes of increased anion gap such as renal failure, salicylate ingestion or other poisoning, or significant ketonemia (e.g., diabetic ketoacidosis, alcohol).

Management of symptomatic subjects with lactate levels of 1 to 2 times the ULN is left to the discretion of the Investigator. As some of the symptoms are sufficiently vague (e.g., fatigue) to be present in everyone, serial repeat testing is encouraged with plans to modify the regimen if the lactate level rises to greater than two times the ULN as outlined above.

Section B. Asymptomatic Hyperlactatemia

In ASYMPTOMATIC subjects, lactic acidosis will be defined as hyperlactatemia greater than four times the ULN. Any patient with a lactate level more than two times the ULN but less than or equal to four times the ULN, should be questioned closely for symptoms (described above) and have a repeat venous sample obtained in 1 week, and, if confirmed, subsequently at monthly intervals.

If the patient fulfills the definition for ASYMPTOMATIC hyperlactatemia, repeat venous lactate should be obtained within a week with confirmation of a more than 4-fold venous elevation in lactate by arterial lactate measurement and arterial blood gas (pH, PO_2, PCO_2, bicarbonate, oxygen saturation) within 48 hours. If confirmed, the patient should be discontinued from the study and alternative therapy instituted. Hyperlactatemia should be followed until levels return to below two times the ULN.

Section C. Specimen Collection

Venous lactate levels are highly dependent on collection techniques. It is therefore recommended that the instructions below be followed closely. High lactate levels should be repeated for verification. If carefully collected, venous lactate level is equivalent to an arterial collection in most clinical situations. If it is not possible to collect the specimen without hand clenching or prolonged tourniquet time, an arterial lactate should be considered, as this will help exclude falsely elevated lactate levels.

1. Have subject sit, relaxed for 5 minutes prior to venipuncture.
2. Instruct subject to not clench the fist before or during the procedure and to relax the hand as much as possible.
3. If possible, do not use a tourniquet. If a tourniquet is necessary, then apply tourniquet lightly and draw lactate first before the other samples with the tourniquet still in place.
4. Collect the blood in a chilled gray-top (sodium fluoride-potassium oxalate) tube.
5. Place the specimen immediately on ice and send to the laboratory for immediate processing, preferably within 30 minutes of collection.
6. If random lactate is elevated, then repeat as above with the following additional patient instructions: no alcohol within 24 hours, no exercise within 8 hours, and no food or drink except water within 4 hours of the draw.
